# Architecture and energy transfer of coccolithophore photosystem I with a huge light-harvesting antenna system

**DOI:** 10.1126/sciadv.aea4965

**Published:** 2025-12-19

**Authors:** Xiao-Meng Sun, Kang Li, Fei-Yu He, Hao-Jie Wang, Hai-Long Chen, Quan Wen, He-Yuan Liu, Fang Zhao, Xiu-Lan Chen, Yu-Xiang Weng, Jun Gao, Lu-Ning Liu, Yu-Zhong Zhang, Long-Sheng Zhao

**Affiliations:** ^1^ State Key Laboratory of Microbial Technology, Shandong University, Qingdao 266237, China.; ^2^ MOE Key Laboratory of Evolution and Marine Biodiversity, Frontiers Science Center for Deep Ocean Multispheres and Earth System & College of Marine Life Sciences, Ocean University of China, Qingdao 266003, China.; ^3^ Laboratory for Marine Biology and Biotechnology, Qingdao Marine Science and Technology Center & Laoshan Laboratory, Qingdao 266237, China.; ^4^ Laboratory of Soft Matter Physics, Institute of Physics, Chinese Academy of Sciences, Beijing 100190, China.; ^5^ Hubei Key Laboratory of Agricultural Bioinformatics, College of Informatics, Huazhong Agricultural University, Wuhan 430070, China.; ^6^ Institute of Systems, Molecular and Integrative Biology, University of Liverpool, Liverpool L69 7ZB, UK.; ^7^ Marine Biotechnology Research Center, State Key Laboratory of Microbial Technology, Shandong University, Qingdao 266237, China.

## Abstract

Coccolithophores play important roles in global biogeochemical cycles and climate change and are characterized by their cell surfaces covered with calcium carbonate coccoliths. Their capacity to thrive in dynamic marine ecosystems is underpinned by efficient photosynthetic adaptation. Here, we elucidated the structure of a photosystem I-light-harvesting complex I (PSI-LHCI) supercomplex from coccolithophore *Emiliania huxleyi* by cryo–electron microscopy, ultrafast spectroscopy, and computational simulations. The PSI core is encircled by 35 LHCIs that are organized in six layers with repetitive, fiber-like arrangements, forming a giant PSI-LHCI supercomplex. Increased levels of chlorophylls c and carotenoids in LHCIs enhance the absorption of blue/green light. Time-resolved spectral analysis and computational simulations both revealed that the large number of pigments forms an extensive pigment network, ensuring efficient energy transfer from peripheral LHCIs to the PSI core. Our study provides insights into the assembly and energy transfer of coccolithophore PSI-LHCI and delineates the evolutionary variation of red-lineage PSI-LHCIs.

## Teaser

The structure of coccolithophore photosystem I with a giant antenna system and intricate energy transfer network.

## INTRODUCTION

Photosystem I (PSI) and photosystem II (PSII) are key membrane protein supercomplexes that capture and convert light energy into chemical energy (*1*, *2*). These multisubunit pigment-protein photosynthetic supercomplexes are composed of two parts: a core complex and a peripheral antenna system that increases the absorption cross section of the photosystems. The core complexes are relatively conserved in oxygenic photoautotrophs, whereas the organization, dimension, protein composition, and pigment association of the antenna systems vary in different organisms (*1*, *2*). In photosynthetic eukaryotes, the PSI antenna system is composed of membrane-embedded light-harvesting complexes I (LHCIs). The structures of various PSI-LHCI supercomplexes have been determined, revealing that the number and arrangement of LHCIs differ substantially among species (*3*). In the green lineage, the green algal PSI core can bind 6, 8, 9, or 10 LHCIs (*4*–*8*), and 4 LHCIs are associated with the plant PSI core (*9*–*14*). In the red lineage, 3, 5, or 8 LHCIs were identified in red algal PSI-LHCIs (*15*–*17*); 11 or 14 LHCIs are associated with the cryptophyte PSI core (*18*, *19*), and the dinoflagellate PSI core has 13, 15, or 18 LHCIs (*20*, *21*); 22 LHCIs were identified in PSI-LHCI from the coccolith-lacking haptophyte (*22*); and 13 LHCIs were identified in PSI-LHCI from Xanthophyceae (*23*), and the diatom PSI core comprises 24 LHCIs, representing a huge PSI-LHCI photosynthetic supercomplex (*24*). Moreover, under high-light conditions, only five LHCIs are bound to the diatom PSI core (*25*, *26*). Such variations in LHCIs suggest the evolutionary process and adaptation of oxygenic photosynthesis to distinct environmental conditions of specific ecological niches.

Coccolithophores, characterized by coccoliths on the cell surface, are unicellular eukaryotic phytoplankton within the Haptophyta lineage. They are widely distributed in the upper layers of both coastal and oceanic waters, usually inhabiting at depths of 0 to 150 m, and can proliferate rapidly, often forming massive blooms that encompass vast oceanic regions (*27*). Coccolithophores emerged in Earth’s oceans ~209 million years ago (Ma), far later than their noncalcifying haptophyte relatives without coccoliths (~1200 Ma) (*28*, *29*). These organisms play a crucial role in global biogeochemical carbon and sulfur cycles closely linked with climate change (*30*). Coccolithophores perform two carbon-pumping mechanisms: photosynthetic carbon fixation and coccolith formation (*27*), playing a substantial role in the natural removal of excess atmospheric CO_2_. Coccolithophores can enhance photosynthesis under low-light conditions. Their unique ability to scatter light could concentrate and amplify the limited available light, thereby increasing light availability within cells (*27*, *31*). Coccolithophores also serve as a natural sunshade, shielding the cells from harmful ultraviolet radiation and mitigating photodamage to the cells in high-light environments (*32*). In addition, coccolithophores are notable for their diverse pigment composition, further augmenting their ability to acclimate to fluctuating light regimes (*33*). Collectively, these intrinsic photoacclimatory mechanisms are vital for optimizing photosynthetic performance, contributing to the ubiquity and prevalence of coccolithophores in marine ecosystems. Advancing our understanding of these processes will require detailed evaluation of the mechanisms governing the structure, light capture, and energy transfer/dissipation of their photosynthetic apparatus.

Here, we determined the structure of the PSI-LHCI supercomplex from *Emiliania huxleyi*, a typical and most abundant coccolithophore species in the modern oceans (*34*, *35*), using single-particle cryo–electron microscopy (cryo-EM). The LHCIs of *E. huxleyi* are identified as fucoxanthin (Fx)-chlorophyll (Chl) a/c–binding proteins (FCPIs) (*36*). Accordingly, *E. huxleyi* PSI-LHCI was designated as PSI-EFCPI (“E” stands for *E. huxleyi*). Structural analysis revealed that PSI-EFCPI is composed of a monomeric PSI core surrounded by 35 EFCPIs, constituting a giant PSI-LHCI supercomplex compared to those characterized in other species. The peripheral EFCPIs exhibit a highly ordered arrangement, facilitating the expansion of the large antenna system. By integrating ultrafast spectroscopy and computational simulations, our study provides insights into protein assembly and energy transfer within the coccolithophore PSI-LHCI, as well as the evolution of red-lineage PSI-LHCI supercomplexes.

## RESULTS AND DISCUSSION

### Overall architecture

**Fig. 1.**
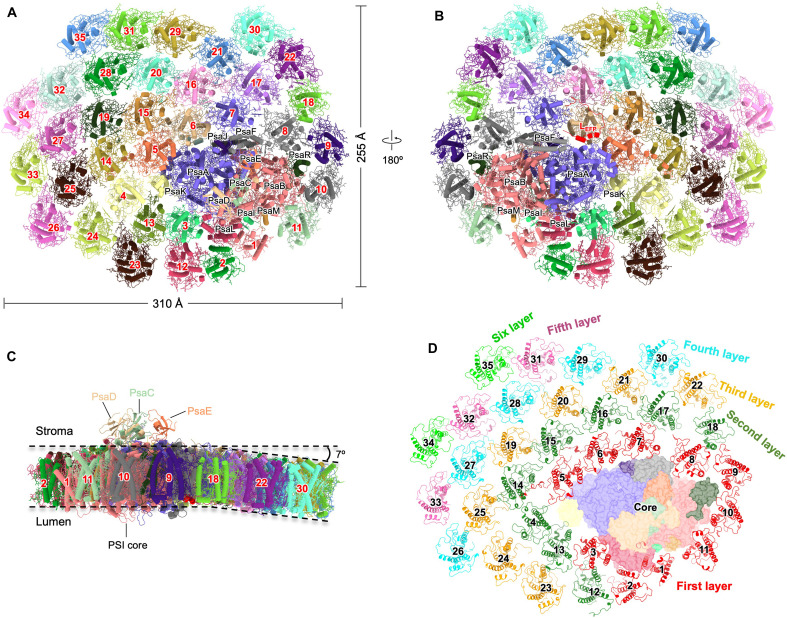
Overall structure of the coccolithophore PSI-EFCPI supercomplex. (**A**) PSI-EFCPI supercomplex viewed from the stromal side. The core subunits are labeled by their names. Numbers 1 to 35 represent EFCPI-1 to EFCPI-35, respectively. (**B**) PSI-EFCPI supercomplex viewed from the lumenal side. The linker protein “L_EFP_” was labeled. (**C**) PSI-EFCPI supercomplex viewed from the EFCPI-9/10/11/18 side. The tilt angle of EFCPIs is indicated. (**D**) Six layers of EFCPIs in the PSI-EFCPI supercomplex.

The PSI-EFCPI supercomplex was purified from *E. huxleyi* and characterized by chromatography, absorption spectroscopy, electrophoresis, mass spectrometry, and pigment composition (fig. S1). The structure was determined at an overall resolution of 3.1 Å using single-particle cryo-EM (fig. S2 and table S1). The overall fan-shaped structure consists of 48 subunits and 790 cofactors with dimensions of 310 Å by 255 Å by 100 Å and a total molecular mass of 1.54 MDa (Fig. 1). The PSI core is surrounded by 35 EFCPI subunits, exhibiting a six-layer arrangement, and a linker protein, L_EFP_ (linker of the EFCPI-PSI core), which forms a large PSI-LHCI supercomplex (Fig. 1). EFCPIs at PsaJ-PsaF were deflected 7° to the lumenal side relative to the PSI core viewed from the EFCPI-9/10/11/18 side (Fig. 1C). In addition, various types of pigments were identified in the PSI-EFCPI by high-performance liquid chromatography (HPLC) (fig. S1E). We assigned 387 Chl a, 112 Chl c, 109 fucoxanthin (Fx), 42 19′-hexanoyloxyfucoxanthin (hFx), 73 diadinoxanthin (Ddx), 3 gyroxanthin-diester (GyrE), 9 Chl c_2_-monogalactosyldiacylglycerol (Chl c_2_-MGDG), and 20 β-carotene (β-Car) in the PSI-EFCPI structure (fig. S3 and table S2). The PsaA/B/C/D/E/F/I/J/L/M/R subunits in the coccolithophore PSI core are conserved among red-lineage PSI-LHCIs (fig. S4A). PsaR is exclusive to red-lineage PSIs, showing structural variabilities in loop regions (fig. S4B) (*4*–*14*). The coccolithophore PSI core contains the PsaK subunit but lacks the PsaO subunit, differing from red algal and cryptophyte PSI cores, where both PsaK and PsaO are present (fig. S4A) (*15*–*19*). In contrast, diatoms and dinoflagellates lack both subunits in their PSI structures (*20*, *21*, *24*). Chl a and a lipid molecule SQDG associate with the specific AB loop of coccolithophore PsaK and locate at the interface between PsaK and EFCPIs, potentially facilitating energy transfer and EFCPI binding (fig. S4, C and D).

A recent study on *E. huxleyi* PSI-LHCI (referred to as Eh-PSI-FCPI) (*37*) revealed similar structural characteristics to those of PSI-EFCPI described in this study (fig. S4, E and F). Compared to Eh-PSI-FCPI, three peripheral LHCIs (Eh-FCPI-14/17/19) were absent in our PSI-EFCPI, which may result from the different growth conditions of cells. Moreover, the peaks corresponding to carotenoids (Cars) 4-keto-19′-hexanoyloxyfucoxanthin (khFx) and GyrE were not detected in our HPLC chromatogram (fig. S1E). khFx is a minor Car in *E. huxleyi* and shares similar structure, absorption spectra, and elution time to hFx. Therefore, the elution peak of khFx may overlap with that of hFx in HPLC analysis, consistent with previous findings (*38*). GyrE was previously detected in a stationary-phase stock culture of *E. huxleyi* (*38*), but it was absent in our HPLC analysis, suggesting that the PSI-EFCPI complexes from exponentially growing cells under our culture conditions contained only trace amounts of GyrE. In GyrE, the esterified tail lies close to the head lacking the ester group, distinguishing its structure from that of hFx. This unique feature allowed identification of corresponding GyrE densities in the map, leading to the assignment of three GyrE molecules.

Furthermore, differences in pigment ratios between our PSI-EFCPI and the reported Eh-PSI-FCPI were observed, which may be attributed to three factors: (i) variations in cell growth conditions, as pigment ratios in *E. huxleyi* can change markedly under different conditions (*33*, *39*); (ii) structural similarity among certain pigments, which, combined with the limited resolution, prevents accurate identification in some areas; (iii) insufficient map resolution in peripheral regions, making precise pigment assignment difficult.

### Structural features of EFCPIs

**Fig. 2.**
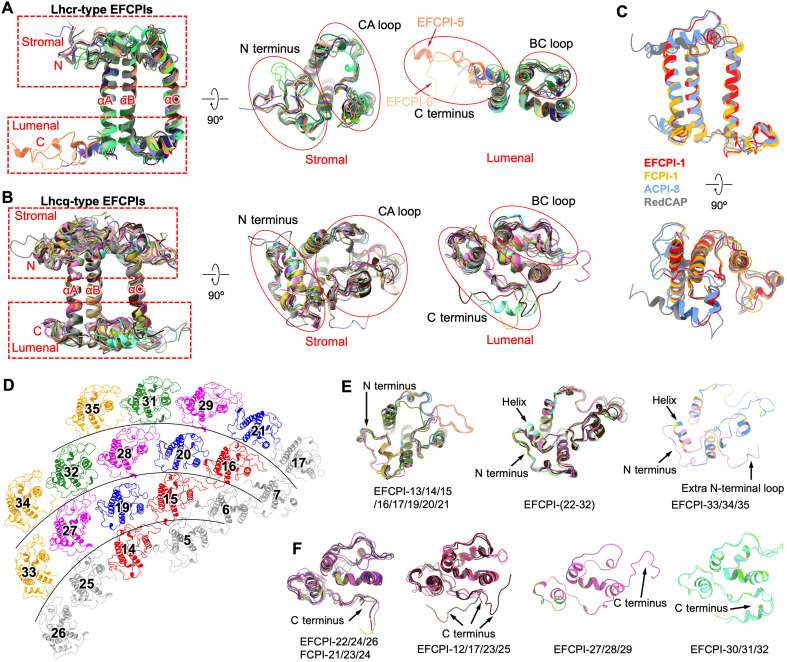
Structures of coccolithophore EFCPIs. (**A** and **B**) Structures of Lhcr-type EFCPIs (A) and Lhcq-type EFCPIs (B). The N terminus, C terminus, and three transmembrane helices were labeled as N, C, αA, αB, and αC, respectively. N-terminal loops, C-terminal loops, BC loops between αB and αC, and CA loops between αC and αA are indicated. (**C**) Structural comparison of RedCAP-type EFCPI-1 with its counterparts in red algae PSI-LHCR (RedCAP) (PDB: 7Y5E), cryptophyte PSI-ACPI (ACPI-8) (PDB: 7Y7B), and diatom PSI-FCPI (FCPI-1) (PDB: 6LY5). (**D**) Structures and locations of EFCPIs with identical sequences. EFCPIs in the same color share the same sequence, except for the gray ones. (**E**) Structure features of N-terminal loops of Lhcq-type EFCPIs. (**F**) Structure features of C-terminal loops of Lhcq-type EFCPIs.

The structures of EFCPI-1 to EFCPI-22 and L_EFP_ were nearly identical to their counterparts in PSI-iFCPI (“i” stands for *Isochrysis galbana*) from coccolith-lacking haptophytes, whereas the extra EFCPI-23 to EFCPI-35 exhibited specific structural features (figs. S5 to S7) (*22*). This indicates that EFCPIs have a close genetic relationship with coccolith-lacking haptophyte iFCPIs. The extra EFCPIs may stem from the evolutionary adaptation of PSI-EFCPI after calcification of coccolithophores. L_EFP_ positions at the lumenal surface of EFCPI-6 and is associated with one Chl a, in line with L_iFP_ (fig. S6A). EFCPIs can be divided into the Lhcr (EFCPI-2/5/6/7/9/10/11/18), Lhcq (EFCPI-8/12–17/19–35), Lhcf (EFCPI-4), CgLhcr9 (“Cg” stands for *Chaetoceros gracilis*) homologs (EFCPI-3), and RedCAP (EFCPI-1) subfamilies, consistent with those of diatom FCPIs, suggesting a close evolutionary relationship (fig. S8) (*24*). All EFCPIs have three transmembrane helices, termed αA, αB, and αC (Fig. 2 and fig. S5), similar to previously reported LHCs from plants and algae (*4*–*21*, *24*). αA and αB are highly conserved in their sequences and structures, whereas the length and tilt direction of αC are variable (Fig. 2, A and B, and figs. S5 and S9). In addition, the terminal loops and loops between helices exhibit various lengths and structural features.

RedCAP-type EFCPI-1 and Lhcr-type EFCPI-5/6/7/9/10/11 share similar structures to their counterparts in red algal PSI-LHCR (light-harvesting complex in red algae), cryptophyte PSI-ACPI (alloxanthin-Chl a/c–binding protein), and diatom PSI-FCPI, indicating that the antenna structures that directly bind to the PSI core are relatively conserved among red-lineage algae (Fig. 2C and fig. S6B) (*15*–*21*, *24*). Specifically, extended C-terminal loops were found in EFCPI-5/6, and an additional helix was identified at the C terminus of EFCPI-7, resembling FCPI-5 (Fig. 2A and fig. S6B). These specific terminal domains interact with peripheral EFCPIs (see the next section), stabilizing the huge antenna system. Counterparts of EFCPI-2 and EFCPI-3 were found in both the diatom PSI-FCPI and dinoflagellate PSI-AcpPCI (Chls a/c-peridinin protein complex) (*20*, *21*, *24*). Unlike EFCPI-2, diatom FCPI-2 and dinoflagellate AcpPCI-2 belong to the Lhcq and Lhcf subfamilies, respectively (fig. S8). They exhibit different structural features, facilitating the binding of adjacent antennae with distinct orientations and structures (fig. S6C). In contrast, EFCPI-3, FCPI-3, and AcpPCI-3 belonged to the same subfamily and shared similar structures, indicating their close evolutionary relationship (fig. S6D). EFCPI-18 exhibited a specific N-terminal loop compared to other Lhcr-type EFCPIs (fig. S6E). Lhcf-type EFCPI-4 displays an extended BC loop and CA loop and a specific N-terminal loop that differs from Lhcr-type EFCPIs, resembling diatom Lhcf-type FCPIs (fig. S6F) (*24*, *40*).

Lhcq-type EFCPIs share similar structures and have specific structural features in the terminal loops, BC loops, and CA loops compared with Lhcr-type EFCPIs, resembling diatom Lhcq-type FCPIs (Fig. 2B) (*24*, *40*). Intriguingly, many Lhcq-type EFCPIs, such as EFCPI-14/15/16, EFCPI-19/20/21, EFCPI-27/28/29, EFCPI-31/32, and EFCPI-33/34/35, have identical sequences and structures (Fig. 2D and fig. S9). Three conformations of the N-terminal loops of Lhcq-type EFCPIs were identified (Fig. 2E). EFCPI-13/14/15/16/17/19/20/21 display similar N-terminal loops. EFCPI-(22–32) have identical N-terminal loops that contain a helix at the end. In addition, the N-terminal loops of EFCPI-34/35 have not only a helix but also an extra loop domain that is not found in diatom Lhcq-type FCPIs (Fig. 2E) (*24*, *40*). The extra loop domain forms interactions with EFCPIs of inner layers (see the next section), playing a critical role in the association of EFCPI-34/35 at the outmost layer. EFCPI-12/17/22–32 have extended C-terminal loops compared to other Lhcq-type EFCPIs (Fig. 2F and fig. S9). Extended C-terminal loops were also identified in the Lhcq-type diatom FCPI-21/23/24 (*24*, *40*), whereas the extended C-terminal loops of the Lhcq-type EFCPIs exhibit more structural conformations to accommodate distinct binding positions (Fig. 2F). The BC and CA loops of the Lhcq-type EFCPIs are longer than those of the Lhcr-type EFCPIs (Fig. 2, A and B, and fig. S7A). Although the CA loops of Lhcq-type EFCPIs exhibit similarities, structural differences were observed among them (fig. S7A), the same as the BC loops (fig. S7B). Compared to other Lhcq-type EFCPIs, some of the CA loops of EFCPI-12/17/19/20/21 are missing (fig. S7A). Likewise, a small portion of the CA loops of EFCPI-19/20/21 is absent compared with those of EFCPI-12/17. Among the other Lhcq-type EFCPIs, the CA loops of EFCPI-14/15/16, CA loops of EFCPI-23/33/34/35, and CA loops of EFCPI-(24–32) display different structural features (fig. S7A). Furthermore, the BC loops of EFCPI-14/15/16, EFCPI-22, EFCPI-23/24, EFCPI-25/26/27/28/29, EFCPI-12/13/19/20/21/30/31/32, and EFCPI-33/34/35 have their own structural characteristics (fig. S7B). The loops of Lhcq-type EFCPI-8 differ structurally from those of other Lhcq-type EFCPIs and have unique structural characteristics compared with the diatom analog FCPI-8, as evidenced by the specific arrangement of neighboring EFCPIs (fig. S7C). In contrast to the PSI-iFCPI complex from haptophytes without coccoliths, the extra Lhcq-type EFCPIs exhibit many structural features exclusive to the coccolithophore PSI-EFCPI, including the extended N-terminal loops of EFCPI-33/34/35, specific C-terminal loops of EFCPI-(23–32), and unique BC loops of EFCPI-(23–29). These loop structures are critical for constructing the huge antenna system of coccolithophore PSI-EFCPI, indicating the evolutionary modification of LHCIs that accompanied the ecological adaptation of coccolithophores following the emergence of calcification.

### Arrangement of EFCPIs

EFCPI-1 to EFCPI-22 share an arrangement pattern similar to that of iFCPI-1 to iFCPI-22 (fig. S10A). However, compared with iFCPIs, a shift occurred on most of the EFCPIs, and the arrangement of antennas in PSI-EFCPI bent toward the lumen (figs. S10A and S11). The conformational heterogeneity of PSI-EFCPI was analyzed, showing no notable tilt angle change of the antenna system, suggesting that the tilt of the antenna system is not a continuous conformational change (fig. S10, B to D, and movie S1). In addition, EFCPI-23 to EFCPI-35 are assembled and attached to the EFCPI-12–14/19–22 side (fig. S10). The additional Eh-FCPI-14/1719 identified in the recently reported coccolithophore Eh-PSI-EFCPI associated with the counterparts of EFCPI-12/23/24, respectively (fig. S4F). Intriguingly, the counterpart of EFCPI-24 is shifted relative to EFCPI-24, which may facilitate the binding of the extra Eh-FCPIs. The locations of EFCPI-1/5/6/7/8/9/10/11 in the innermost layer are in agreement with those of their counterparts in other red-lineage PSI-LHCIs (fig. S12, A to D) (*15*–*21*, *24*). The LHCIs binding at the PsaK-PsaO-PsaL side show diversified organizations among red-lineage PSI-LHCIs as a result of the presence and absence of PsaK and PsaO subunits (fig. S12E). PsaO is absent in the coccolithophore, diatom, and dinoflagellate PSI cores, and its position is occupied by EFCPI-3 in coccolithophores, FCPI-3 in diatoms, and AcpPCI-3 in dinoflagellates (fig. S12E). The three LHCIs share conserved structures and locations. The coccolithophore PSI core contains PsaK subunits, which are missing in diatom and dinoflagellate PSI cores, resulting in variations in the arrangement of EFCPI-4/13 relative to their counterparts in diatoms and dinoflagellates (fig. S12E). EFCPI-4 exhibits a ~180° rotation relative to FCPI-4, forming a centrosymmetric structure with adjacent EFCPI-13 (fig. S12F).

The EFCPIs in the second to sixth layers belong to the Lhcq subfamily, except for EFCPI-4/18 (fig. S8). Most EFCPIs, with identical sequences and structures, are located in the same layer (Fig. 1D). The structural similarities of the Lhcq-type EFCPIs facilitate the regular arrangement of the EFCPIs in the second to sixth layers, forming a repetitive fiber-like structure (Fig. 2D and fig. S12A). The unique structural features observed in the coccolithophore PSI-EFCPI facilitate continuous expansion of its antenna complex, resulting in the formation of the most extensive antenna system currently identified among all known photosynthetic organisms.

The distinct N-terminal loops of Lhcq-type EFCPIs play critical roles in the assembly of peripheral EFCPIs, facilitating interactions with EFCPIs in both the same layer and adjacent layers on the stromal side (fig. S13A). EFCPIs in the second to sixth layers, except EFCPI-13/18, interact with the CA loops of EFCPIs in the inner adjacent layers and with the CA loops and αC helix of EFCPIs in the outer adjacent layers via their N-terminal loops (fig. S13A). The EFCPI-13_second layer_ associates with the N-terminal loop of the EFCPI-3_first layer_. The EFCPI-18_second layer_ associates with the first layer through its CA loop. The long N-terminal loops of the EFCPI-34/35_sixth layer_ also form interactions with the CA loops of the EFCPIs_fourth layer_ (fig. S13A). The interactions between neighboring EFCPIs in the second layer and EFCPI-23/24/25_third layer_ are mediated by their N-terminal loops and CA loops, whereas other EFCPIs lack direct interactions with neighboring EFCPIs in the same layer because of large gaps between them (fig. S13A). Furthermore, the extended C-terminal loops of EFCPI-5/6/7_first layer_ and the linker protein L_EFP_ facilitated the association of EFCPI-14/15/16 at the lumenal side (fig. S13B).

In addition, the specific C-terminal loops and BC loops of the Lhcq-type EFCPIs on the lumenal side also play essential roles in the assembly of the peripheral antennae (fig. S13B). In the second layer, the extended BC loops of EFCPI-4/12/13 and the extended C-terminal loops of EFCPI-12/17 participate in the assembly of EFCPI-4/12/13/17 (fig. S13B). In the third layer, the extended BC loops of EFCPI-19/20/21, the αC helices of EFCPI-19/20/21/23/25, and the extended C-terminal loops of EFCPI-25 form interactions with the C-terminal loops of the EFCPIs_second layer_, and the extended C-terminal loops of EFCPI-24 interact with the extended BC loop of the EFCPI-4_second layer_ (fig. S13B). In the fourth layer, the extended C-terminal loops of EFCPI-27/28/29 and the αC helices of EFCPI-26/30 facilitate the binding of the EFCPIs_fourth layer_ via interactions with the C-terminal loops of the EFCPIs_third layer_ (fig. S13B). In the fifth layer, the extended C-terminal loops of EFCPI-31/32 are associated with the extended C-terminal loops and BC loops of EFCPI-27/28_fourth layer_ (fig. S13B). In the sixth layer, the extended BC loops of EFCPI-34/35 interact with the C-terminal loops of EFCPIs in the fifth layer (fig. S13B).

Overall, the structural similarity and extended loops of Lhcq-type EFCPIs, as well as the linker protein L_EFP_ (fig. S6A), provide the structural basis for the ordered organization of EFCPIs and are essential for the construction of the giant antenna system in coccolithophore PSI-LHCI. Coccolithophore PSI-LHCI contains 13 additional Lhcq-type LHCIs than coccolith-lacking haptophyte PSI-LHCI. These extra LHCIs feature longer and more diverse terminal loops than Lhcq-type LHCIs of the coccolith-lacking haptophyte (Fig. 2), facilitating the expansion of coccolithophore antennae. In contrast, diatom PSI-LHCI contains 24 LHCIs, including 11 of the Lhcq type, and the LHCI arrangement in diatom PSI-LHCI is irregular and distinct from that of coccolithophore PSI-LHCI (fig. S12C). Collectively, these findings reveal a distinct strategy of antenna system expansion in coccolithophore PSI-LHCI.

The difference in the number of peripheral LHCIs between coccolith-lacking haptophyte PSI-LHCI and coccolithophore PSI-LHCI is unlikely caused by the purification process, given that both *E. huxleyi* and coccolith-lacking haptophyte *I. galbana* were cultured under identical conditions and their PSI-LHCIs were purified using the same procedures (*22*). Whether haptophyte PSI is capable of binding more LHCIs under specific conditions, such as very low light intensity, remains to be further investigated.

In addition, more sequences of Lhcq-type EFCPIs were identified (fig. S13C), implying that coccolithophore PSI may be capable of associating with a larger number of LHCIs, and the current structure might be an intermediate structure of an even larger assembly. The recent study showed that coccolithophore Eh-PSI-FCPI can bind up to 38 FCPIs (*37*), which may result from different culturing conditions.

### Pigment arrangement in EFCPIs

The EFCPI antenna system contains substantial amounts of pigments, including 291 Chl a, 121 Chl c, and 224 Car molecules (table S2). On average, each EFCPI contains 3.46 Chl c and 6.4 Cars, more than those of coccolith-lacking haptophyte iFCPIs (2.82 Chl c and 4.64 Cars), cryptophyte ACPIs (1.35 Chl c and 5.07 Cars), and diatom FCPIs (1.42 Chl c and 5.54 Cars) (*18*, *22*, *24*). These suggest efficient blue/green light absorption by EFCPIs, corresponding to the enhanced absorption of PSI-EFCPI at 450 to 550 nm (fig. S1C). A total of 92.6% of the Chl c molecules are located in the Lhcq- and Lhcf-type EFCPIs, each containing four to seven Chl c molecules (fig. S14A and table S2).

**Fig. 3.**
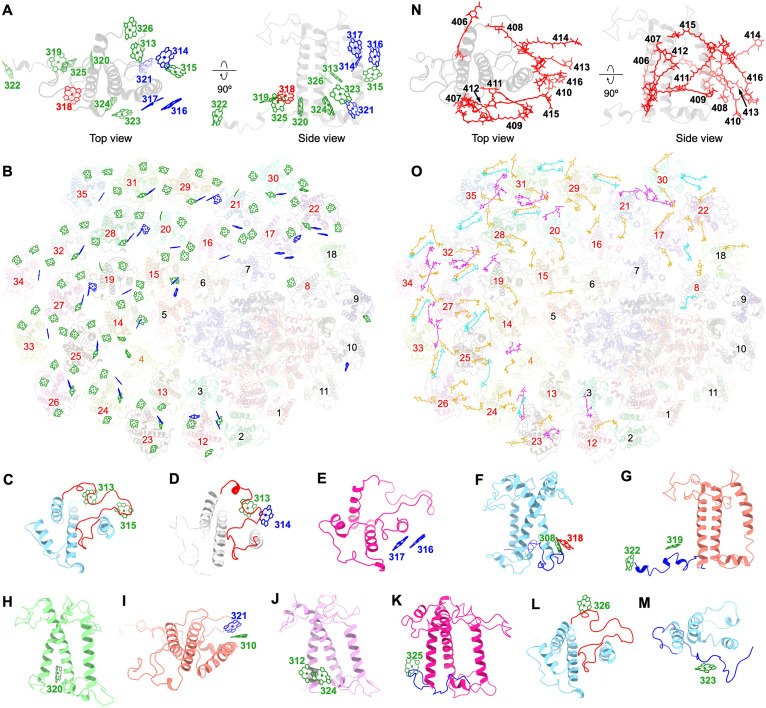
Pigment-binding sites in EFCPIs. (**A**) Numbering of the 14 specific Chl sites in EFCPIs with a side view and top view. Green Chls indicate Chl a. Blue Chls indicate Chl c. Red Chls are mix sites for Chls a and c. (**B**) Arrangement of specific Chls in EFCPIs. Green and blue Chls are Chls a and c, respectively. Numbers 1 to 35 represent EFCPI-1 to EFCPI-35, respectively (black: Lhcr-type EFCPIs; orange: Lhcf-type EFCPIs; red: Lhcq-type EFCPIs). (**C** to **M**) Locations of Chls 313 to 326 in EFCPIs. (**N**) Numbering of the 11 specific Car sites in EFCPIs with a side view and top view. (**O**) Arrangement of specific Cars in EFCPIs. Orange, purple, and cyan Cars are Fx, hFx, and Ddx, respectively. Numbers 1 to 35 represent EFCPI-1 to EFCPI-35 (black: Lhcr-type EFCPIs; orange: Lhcf-type EFCPIs; red: Lhcq-type EFCPIs).

There are 26 Chl-binding sites in EFCPIs, 12 of which are conserved Chl-binding sites (301 to 312) found in red-lineage LHCIs (fig. S14B and table S3) (*15*–*21*, *24*), and 14 Chl-binding sites (313 to 326) are distributed differently among the EFCPIs (Fig. 3, A and B, and table S3). Ten (313 to 318/320/323/325/326) of these 14 sites are located in the Lhcq-type EFCPIs, and 7 (316/319/320/322/324 to 326) are unique to EFCPIs. The Chl c_2_-MGDG molecules, which function in transporting Chl c_2_ from the MGDG-rich chloroplast envelope membrane to LHCs (*41*, *42*), are widely distributed in haptophytes. They were found in EFCPI-3/8/14/15/16/22 at the Chl 310 site and in EFCPI-19/20/21 at the Chl 318 site (fig. S15A). These Chl c_2_-MGDG molecules are presumed to be involved in light harvesting and contribute to the assembly of EFCPIs (fig. S15B) (*43*).

The Chl a–binding sites 313 and 315 were identified within the extended CA loops of Lhcq-type EFCPIs (Fig. 3C). However, EFCPI-8/12/17/18/19/20/21 had shorter CA loops than other Lhcq-type EFCPIs, leading to the absence of sites 313/315 in EFCPI-8/18 and 315 in EFCPI-12/17/19/20/21 (fig. S7A and table S3). In addition, the CA loops of EFCPI-12/17 contain an extra domain for Chl c314 binding (Fig. 3D). Chl c316 associates with αC of EFCPI-12/17/22/24/27/28/29, forming a pair with Chl c317 (Fig. 3E). Chl c317 and Chl 318 are present in most Lhcq-type EFCPIs (table S3). Chl 318 binds to the specific C-terminal loops of Lhcq-type EFCPIs and is near Chl 308 (Fig. 3F). Chl a319 is associated with the extended C-terminal loop of EFCPI-5/6, and Chl a322 is located at the C terminus of EFCPI-5 (Fig. 3G). Chl a320, Chl 321, Chl a324, Chl a325, and Chl a326 bind to the αB of EFCPI-19/20/21 (Fig. 3H), BC loops of EFCPI-5/9/10 (Fig. 3I), αA of EFCPI-4 (Fig. 3J), C-terminal loops of EFCPI-22 (Fig. 3K), and CA loops of EFCPI-25 (Fig. 3L), respectively. Chl c321 and Chl a324 are near Chl 310 and Chl a312, respectively (Fig. 3, I and J). Chl a323 associates with the extended C-terminal loops of the Lhcq-type EFCPIs that are close to the EFCPIs in the inner layers (Fig. 3M). These 14 Chl-binding sites are all located at the interfaces between EFCPIs, suggesting their potential roles in promoting excitation energy transfer (EET) between adjacent EFCPIs (Fig. 3B).

Nonconserved Chl-binding sites 313/314/315/317/318/323 were also present in diatom Lhcq-type FCPIs but absent in Lhcq-type LHCIs of dinoflagellate and yellow-green alga *Tribonema minus*. Only one Lhcq-type LHCI was identified in dinoflagellate PSI-LHCI and *T. minus* PSI-LHCI, and their loop structures differ substantially from Lhcq-type EFCPIs, leading to the absence of structural features for the binding of nonconserved pigments in Lhcq-type EFCPIs (fig. S15C) (*20*, *23*). Chl-binding sites 313/314/315/317/318/323 in Lhcq-type EFCPIs share similar structural features to those in diatom Lhcq-type FCPIs (fig. S15D). By contrast, the binding of Chls 316/320/325/326 is distinctive to Lhcq-type EFCPIs. In EFCPI, Chl 316 is coordinated by a His residue, substituted by Leu in diatom FCPI, which likely accounts for the absence of Chl 316 (fig. S15D). A Car is adjacent to Chl 320 in EFCPI, and its counterpart in diatom FCPI is shifted to occupy the Chl 320 position (fig. S15E). The loop conformation for Chl 325 binding in EFCPI varied in diatom FCPI, where the LEU residue sterically clashes with Chl 325 (fig. S15F). Chl 326 binding is further facilitated by the surrounding EFCPIs and pigments in EFCPI, which are absent in diatom FCPIs (fig. S15G).

A large number of Cars were identified in the 35 EFCPIs, including 109 Fx, 42 hFx, 73 Ddx, and 3 GyrE (fig. S14C and table S2). The major Cars in the Lhcr-type EFCPIs are Ddx, whereas those in the Lhcq-type EFCPIs are Fx. Fx plays a critical role in quenching excess light energy (*44*), indicating the enhanced energy-quenching capacity of Lhcq-type EFCPIs. The xanthophyll cycle Cars Ddx and diatoxanthin (Dtx) were identified in PSI-EFCPI using HPLC (fig. S1E), suggesting the existence of a Ddx-Dtx cycle for energy dissipation (*45*, *46*). Compared to Fx, hFx has a hexanoyloxy tail and exists only in Lhcq-type EFCPIs (fig. S3 and table S3). The conjugated keto group introduces an intramolecular charge transfer (ICT) state for Fx, facilitating energy transfer between Fx and Chl (*36*, *47*). However, the hexanoyloxy tail decreases the effect of polarity on the excited-state dynamics of hFx, thereby suppressing its ICT state. This potential slows down Car-Chl energy transfer. Under high-light conditions, Fx is substituted by hFx in *E. huxleyi* (*48*), suggesting that hFx may promote photoprotection under high-light conditions by reducing energy transfer from Cars to Chls.

All Cars are located in 16 Car-binding sites of EFCPIs, among which five (401 to 405) are conserved in red-lineage LHCIs (fig. S14D) (*15*–*21*, *24*) and five (408/409/410/415/416) are unique to EFCPIs (Fig. 3N). The 409/413 to 416 sites were not found in iFCPIs from coccolith-lacking haptophytes, as these sites were identified in the EFCPIs whose counterparts were absent in PSI-iFCPI (table S3). The conformational shift of Chl 321 relative to Chl 310 led to a swing in the lumenal part of Car 402 in EFCPI-5/9/10 (Fig. 3I and fig. S14D). The specific C-terminal loops of Lhcq-type EFCPIs and the shift of Chl 318 relative to Chl 308 led to the swing of the lumenal part of Car 405 (Fig. 3F and fig. S14D). Among the 406 to 416 sites, Car 407 and Car 408 are exclusive to EFCPI-4 and EFCPI-5, respectively; other sites only exist in Lhcq-type EFCPIs, except Car 411, which is also found in Lhcf-type EFCPI-4 (table S3). Consequently, more Cars are situated in Lhcq-type EFCPIs than in Lhcr-type EFCPIs. The 406 to 416 sites position at the interfaces between EFCPIs and interact closely with Chls, which may facilitate intersubunit energy transfer and quenching (Fig. 3O).

Nonconserved Car-binding sites 406/411/412/413/414 in Lhcq-type EFCPIs are also found in diatom Lhcq-type FCPIs, and their structural features are conserved in Lhcq-type EFCPIs and diatom Lhcq-type FCPIs (fig. S15H). By contrast, the Car-binding sites 409/410/415/416 are unique to Lhcq-type EFCPIs. The extended C-terminal loop facilitating the binding of Car 409 is absent in diatom Lhcq-type FCPIs (fig. S15I). The extended loops in diatom Lhcq-type FCPIs sterically hinder Car 410 and prevent its binding (fig. S15J). The extended N-terminal loop facilitating the binding of Car 415/416 is absent in diatom Lhcq-type FCPIs (fig. S15K). Therefore, the structural features for the binding of Car 409/410/415/416 are unique to Lhcq-type EFCPIs.

Among the 12 conserved Chl-binding sites and 5 conserved Car-binding sites, Chls 301/308/311/312 and Car 404 are absent in the vast majority of Lhcq-type EFCPIs (table S3). These pigments are located on the same side of the EFCPI, and their functions are compensated by Chls 316/317/318/323 and Cars 411/412 on the same side of the Lhcq-type EFCPIs (Fig. 3). The absence of Chl 301 and 308 is due to structural changes in the N-terminal and C-terminal loops of the Lhcq-type EFCPIs, respectively (Fig. 2, A and B).

### EET within PSI-EFCPI

**Fig. 4.**
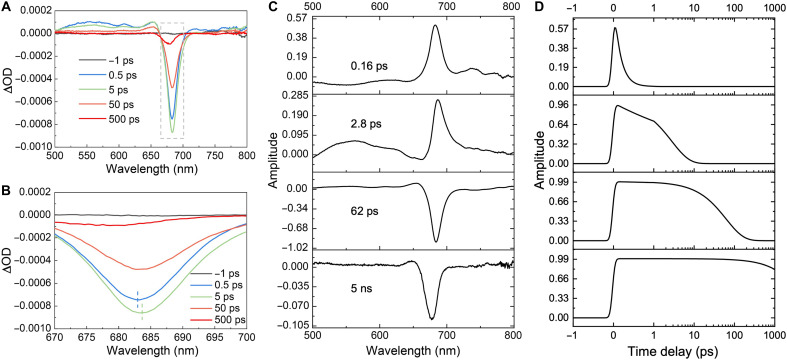
Ultrafast absorption and fluorescence spectroscopic analysis of PSI-EFCPI. (**A**) Femtosecond time-resolved TA spectra. The excitation wavelength was set at 445 nm. Five time-resolved spectra at −1, 0.5, 5, 50, and 500 ps are shown. The boxed area is shown in (B). ΔOD, change in optical density. (**B**) Magnified view of the boxed area in (A). The peaks of the 0.5- and 5-ps spectra are indicated. (**C**) TA decay–associated spectra of PSI-EFCPI. Positive values represent the growth of the corresponding component, whereas negative values indicate decay processes. (**D**) Dynamics of the four corresponding TA decay–associated components of PSI-EFCPI.

The large PSI-EFCPI supercomplex provides a framework for a large and sophisticated pigment network. To study EET within PSI-EFCPI, we measured time-resolved transient absorption (TA) spectra and fluorescence spectra of PSI-EFCPI and obtained decay-associated (DA) spectra by global analysis (Fig. 4). After ultrafast excitation, a pronounced negative peak rapidly emerged near 684 nm in the TA spectra, originating from the superposition of ground-state bleaching (GSB) and stimulated emission (SE) signals associated with the Q_y_ band of Chl a (Fig. 4A). Simultaneously, a broad positive signal appeared in the 500- to 650-nm region, which was primarily attributed to the excited-state absorption of Chl a. Within a few picoseconds, the amplitude of the GSB or SE signals rapidly increased to its maximum around 685 nm, accompanied by a red shift of ~1 nm in the central peak position (Fig. 4B). Subsequently, the overall signal intensity gradually decayed over tens of picoseconds. Ultimately, only a spectral component with a lifetime of approximately a few nanoseconds remained, and the central peak of the negative signal blue-shifted to ~677 nm.

Four main DA spectral components and the corresponding dynamics were obtained from the TA spectra (Fig. 4, C and D). We mainly focused on the GSB and SE signal region (660 to 710 nm) in the DA spectra. In the fastest time component (~0.16 ps), a predominantly positive signal was observed near 680 nm. This process involves relaxation from the Soret band to the Q_y_ band in Chl a as well as a possible energy transfer from Chl c/Car to Chl a. Consistently, a previous study on diatom Fx-Chl a/c–binding proteins indicated that energy transfer from Chl c/Fx to Chl a occurred at hundreds of femtoseconds (*49*). In addition, the signal near 680 nm exhibits a growth process with a time constant of ~2.8 ps. Compared to the first process, the DAS peak showed a slight red shift in the central wavelength, became negative in the region below 670 nm, and displayed more pronounced positive signals above 700 nm. This corresponds to the excited-state energy transfer from the most inner layer of EFCPIs to the PSI core. Notably, similar time constant around 2.8 ps was also observed in cryptophyte and haptophyte PSI-LHCI supercomplexes with considerably different sizes of peripheral antenna systems, supporting the above assignment. Then, a dominant energy decay process with a time constant of ~62 ps was recorded, which indicates the energy transfer from EFCPIs to the PSI core. Intriguingly, the measured time constant of 62 ps is greater than that of the corresponding energy transfer in cryptophyte PSI-LHCI (~53 ps, minimal size) (fig. S16), owing to the enlarged antenna system of PSI-EFCPI. Moreover, this time constant is similar to that of coccolith-lacking haptophyte PSI-iFCPI (~63 ps, intermediate size), suggesting that the larger antenna system with extra EFCPI-(23–35) in PSI-EFCPI maintains efficient EET relative to haptophyte PSI-iFCPI. The 5-ns component with a blue-shifted negative peak below 680 nm primarily originated from uncoupled Chl a molecules that cannot transfer excitation energy to other pigments because the time constant is close to the lifetime of the S_1_ state of Chl a (Fig. 4C) (*50*). The proposed energy transfer pathways within PSI-EFCPI based on the analysis of TA spectra were also validated by time-resolved fluorescence measurements (fig. S16G). The 642-nm peak in the first fluorescence DA (FDA) spectrum of the cryptophyte PSI-LHCI is notably weaker than that of coccolithophore PSI-EFCPI, likely due to the large number Chl c in PSI-EFCPI (fig. S16H).

**Fig. 5.**
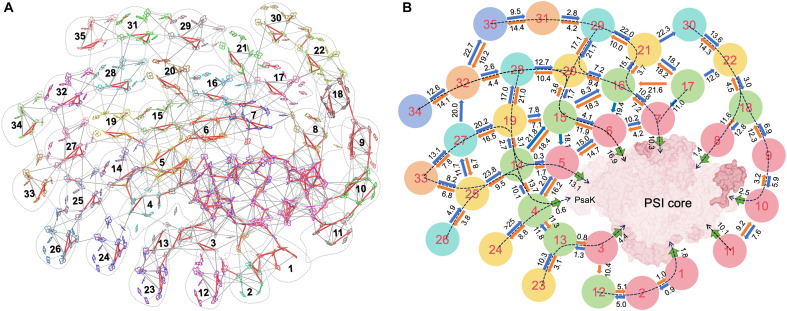
Pivotal EET pathways in PSI-EFCPI. (**A**) Top view of the map of interpigment EET rates. The color bars correspond to rates faster than 1 ps (red), in the range of 1 to 10 ps (thick white), and in the range of 10 to 20 ps (thin black). Rates slower than 20 ps are omitted from the analysis. (**B**) EET rates labeled in picoseconds between EFCPIs (thick blue and orange arrows) and from EFCPIs to the PSI core (thick green arrows). Rates longer than 25 ps are omitted from the analysis. The relatively efficient EET pathways from the EFCPIs to the PSI core are indicated by dashed arrows. The EET pathways can be divided into two groups with similar rates, as they traverse EFCPI-14, EFCPI-18, EFCPI-20, EFCPI-28, and EFCPI-29. After passing through EFCPI-19 and EFCPI-21, the EET routes shifted to adjacent EFCPI fibers, establishing more efficient pathways. Most of the outer EFCPIs transfer energy to the PSI core through EFCPI-5/6/7, indicating their critical role in EET within PSI-EFCPI. In addition, EFCPI-1/2/3/8/9/10 also mediated EET from the outer EFCPIs to the PSI core.

Furthermore, we used our structural data on pigment arrangement to model the energy transfer within PSI-EFCPI. We used the Förster theory to computationally simulate the EET rates (time constants) between all Chl pairs (Fig. 5A) (*51*) and the generalized Förster theory, an extension of the classical Förster theory (Fig. 5B) (*52*), to assess the EET rates between EFCPIs as well as between EFCPIs and the PSI core. In accordance with the time-resolved spectral analysis (Fig. 4), quantum simulation revealed efficient EET within PSI-EFCPI. The simulated time constants of EET between adjacent EFCPI layers as well as between the innermost EFCPI layer and the PSI core range from a few picoseconds to more than 20 ps (Fig. 5, A and B). The time constants of EET from EFCPI-1/3/4/8/10 are relatively fast, with an average time of 2.14 ps, which may be responsible for the 2.8-ps component in DA spectra (Fig. 4C). EET between neighboring EFCPIs within the third to sixth layers appeared less efficient because of the large gap between them. Consequently, the main EET routes from the outermost EFCPIs to the PSI core predominantly follow the EFCPI fibers (Fig. 5B). The energy equilibrium process takes place during energy transfer along the EFCPI fibers to the PSI core. The average time constant of the EET routes from the EFCPIs in fifth and sixth layers to the PSI core is 62.5 ps, which fits perfectly with time-resolved spectral analysis (Fig. 4C, fig. S17A, and table S4). The uniform structure and regular arrangement of EFCPIs contribute to a more structured EET network, facilitating efficient EET from the peripheral EFCPIs toward the PSI core.

The EET from the innermost layer to the PSI core is mainly mediated by Chl 305/306 pairs on the stromal side and Chls 311/312 on the lumenal side (fig. S17, B and C), which is in agreement with other red-lineage PSI-LHCIs (*15*–*21*, *24*). Because of the specific orientation of EFCPI-1/2/3, Chl 309_EFCPI-1_, Chl 304_EFCPI-2_, and Chls 302/303/304/309_EFCPI-3_ are involved in EET to the PSI core. These Chls at the interface of the EFCPI and PSI core may be responsible for the 688-nm peak in the first FDA spectrum (fig. S16D). The stromal EET between the second and first layers is mainly mediated by Chls 305/306/307 in the second layer and Chls 302/303/304 in the first layer (fig. S17B). Owing to the specific orientation of EFCPI-3/4/12/13, diverse EET routes were established. In addition, the specific Chl 319_EFCPI-5_, Chls 313/314/315_EFCPI-12_, and Chls 315/317_EFCPI-14/15/16_ facilitate energy transfer by forming extra EET pathways. Chls 305/306/307/313/317 of EFCPI-19/20/21_third layer_ and Chls 313/315/318 of EFCPI-14/15/16/17_second layer_ constitute numerous stromal EET pathways (fig. S17B). Unlike EFCPI-19/20/21, EFCPI-22/23/24/25_third layer_ primarily transferred energy to EFCPIs_second layer_ via their Chl 315. Efficient EET pathways from Chl 305_EFCPI-23/25_ to Chl 318_EFCPI-13/14_ were also identified. Chls 305/306/315 of the EFCPIs in the fourth to sixth layers play an important role in mediating stromal EET (fig. S17B). In addition, Chls 313/318/320/326 are also involved in EET. On the lumenal side, fewer EET routes were found from the sixth to first layers, which are mainly mediated by Chls 309/318/319/320/323 and Chl 201 of L_EFP_ (fig. S17C). Chl 323_EFCPI-31/32_ in the fifth layer also forms EET with Chl 320_EFCPI-19/20_ in the third layer. Intriguingly, the majority of lumenal EET occurs between the EFCPI fibers. Moreover, efficient EET was also identified between adjacent EFCPIs within the first and second layers (fig. S18). The EET between EFCPI-4 and EFCPI-14 is facilitated by Chl 322 binding at the extended C-terminal loop of EFCPI-5.

Overall, we dissected the progress of EET from peripheral EFCPIs to the PSI core on the basis of the results of the ultrafast spectroscopic measurements and computational simulation with Förster theory; both the experimental and theoretical results consistently revealed a slower antenna-PSI energy transfer within coccolithophore PSI-EFCPI compared to cryptophyte PSI-LHCI because of the enlarged antenna system. Our analysis also suggests that the EET of PSI-EFCPI on the stromal side is more efficient than that on the lumenal side. The specific Chls 313/315/317/318/319/320/322/323/324/326, Chl 203_PsaK_, and Chl 201 of L_EFP_ play critical roles in mediating the EET from outermost EFCPIs to the PSI core. The emergence of these Chls is compatible with the unique arrangement of the antenna system in coccolithophore PSI-EFCPI, facilitating EET from the giant antenna system to the PSI core.

### Insights into the evolution of red lineage PSI-LHCIs

**Fig. 6.**
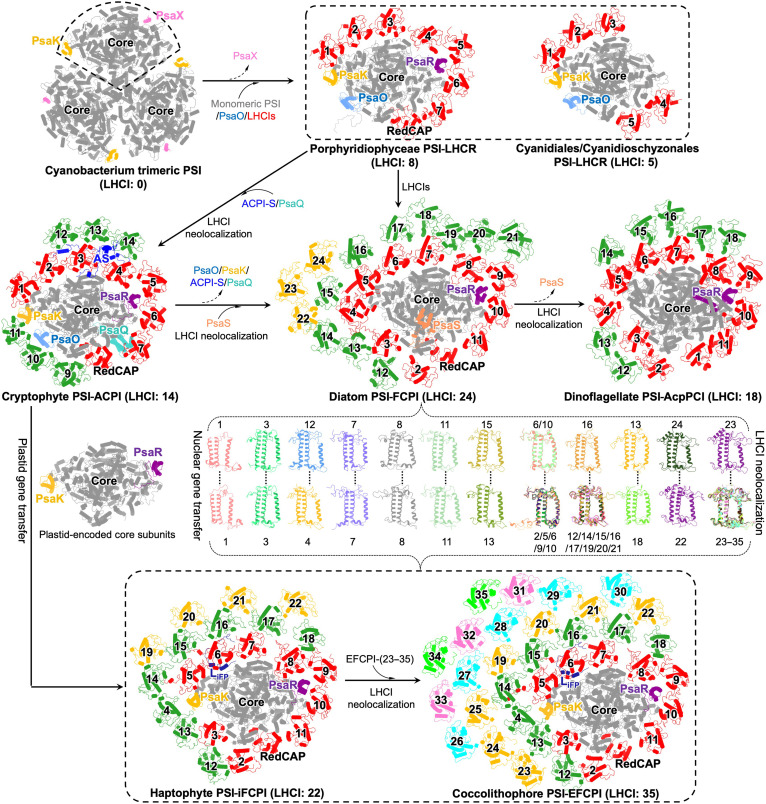
Estimated evolutionary trajectory of red-lineage PSI-LHCI supercomplexes. PDB codes of PSI structures: trimeric PSI of cyanobacterium *Synechococcus elongatus* (1JB0); PSI-LHCI of red alga *Cyanidium caldarium* (8WEY); PSI-LHCR of red alga *Porphyridium purpureum* (7Y5E); PSI-ACPI of cryptophyte *Chroomonas placoidea* (7Y7B); PSI-FCPI of diatom *C. gracilis* (6LY5); PSI-iFCPI of haptophyte *I. galbana* (8Z11). The numbers represent the quantity of LHCIs. Coccolithophore EFCPIs and diatom FCPIs in same clade share similar structures (brace), suggesting their close genetic relationship. The coccolithophore PSI core shares a similar structure to the cryptophyte PSI core, especially the presence of PsaK subunits. “neolocalization” represents an evolutionary phenomenon defined as structural relocalization and modifications of LHCs (*17*, *58*).

Red-lineage algae, such as cryptophytes, haptophytes, ochrophytes (including diatoms), and dinoflagellates, originate from red algae via multiple endosymbiosis. These algae are speculated to be derived from single red alga–derived secondary endosymbiosis, and the red plastids spread across the red-lineage tree through a series of endosymbiosis (*53*–*57*). The monomeric PSI core of red algae was derived from the trimeric PSI of cyanobacteria through primary endosymbiosis. The monomeric PSI core makes it possible to bind to the PsaO subunit, which mediates the interaction between PSI and PSII. The red algal PSI core associates with LHCIs that are absent in cyanobacteria, forming two types of PSI-LHCI supercomplexes: the PSI core with five LHCIs in Cyanidiales/Cyanidioschyzonales and eight LHCIs in Porphyridiophyceae (Fig. 6) (*15*–*17*). Porphyridiophyceae PSI-LHCI contains an additional PsaR subunit that mediates the association of three extra LHCIs. In addition, a specific LHCI, RedCAP, was identified in Porphyridiophyceae PSI-LHCI, whereas it had been substituted by Lhcr-type LHCI in Cyanidiales/Cyanidioschyzonales PSI-LHCI (Fig. 6). PsaR and RedCAP were also found in haptophyte, cryptophyte, and diatom PSI-LHCIs. Moreover, the arrangement of LHCIs in Porphyridiophyceae PSI-LHCI is in accordance with that of their counterparts in cryptophyte, haptophyte, diatom, and dinoflagellate PSI-LHCIs (*15*, *18*, *21*, *24*). These structural similarities indicate that red-lineage PSI-LHCIs were derived from Porphyridiophyceae PSI-LHCI.

Cryptophytes acquire plastids from red algae through secondary endosymbiosis. During endosymbiosis, red algal phycobilisomes degenerated to phycobiliproteins in cryptophytes (*18*, *54*, *58*–*60*). Cryptophyte PSI-ACPI has a larger LHCI system than red algal PSI-LHCI. Six additional LHCIs are associated with cryptophyte PSI in the form of trimers via the mediation of PsaK/O and the linker protein ACPI-S. Except for RedCAP, cryptophyte LHCIs belong to the Lhcr subfamily, consistent with red algal LHCIs (*18*). Cryptophyte LHCIs exhibit similar structures and organization to red algal LHCIs. These structural similarities suggest that cryptophyte PSI-LHCI originated from red algal PSI-LHCI (Fig. 6).

Diatom PSI-FCPI and dinoflagellate PSI-AcpPCI lost the PsaK/O subunits and incorporated more LHCIs compared with cryptophyte PSI-LHCI. Distinct from cryptophyte LHCIs, diatom and dinoflagellate LHCIs have diversified into the Lhcr, Lhcq, and Lhcf subfamilies. Ochrophytes (including diatoms) engulfed cryptophytes via tertiary endosymbiosis, as suggested by nuclear genome analysis (*57*). Phylogenetic analysis showed that except for FCPI-11, Lhcr-type LHCIs of diatoms and cryptophytes have a close genetic relationship (*58*). FCPI-11 is in the same group as red algal Lhcr2, and they share the same structures and binding positions. These findings suggest that diatom PSI-LHCI lost the PsaK/O subunits from cryptophytes and that their LHCIs were derived from cryptophytes and red algae (Fig. 6). The position of PsaO is occupied by AcpPCI-3 in dinoflagellate PSI-AcpPCI and FCPI-3 in diatom PSI-FCPI. AcpPCI-3 and FCPI-3 have similar structures and orientations (*20*, *21*, *24*, *40*). In addition, the arrangement of AcpPCIs is similar to that of their counterparts in diatoms (Fig. 6). Previous studies have suggested that the dinoflagellate peridinin plastid originated from ochrophytes (*61*–*63*). The structural features of dinoflagellate PSI-AcpPCI support the derivation of dinoflagellate PSI-LHCI from ochrophyte PSI-LHCI.

Ochrophytes are engulfed by haptophytes during serial endosymbiosis in red-lineage algae, as suggested by nuclear genome analysis (*57*). However, plastid gene analysis supports cryptophyte-haptophyte monophyly in plastid phylogenies, implying that haptophytes may have incorporated ancestral cryptophyte plastids (*53*). The plastid gene *rpl36* was replaced by a bacterial homolog in both cryptophytes and haptophytes. Thus, it has been proposed that plastids were transferred from the cryptophyte to haptophyte after the *rpl36* replacement (*64*, *65*). Structural analysis of haptophyte PSI-EFCPI from coccolithophores provides advanced knowledge of the evolution of red-lineage photosynthesis.

It is worth noting that coccolithophore PSI-EFCPI has the plastid-encoded PsaK subunit in the PSI core, which is present in the cryptophyte PSI core and absent in the diatom PSI core, supporting the notion that haptophytes acquired plastids from cryptophytes. Thus, the plastid-encoded PSI core of haptophytes may be derived from the cryptophyte PSI core. The light-harvesting complex genes are nuclear encoded, and three LHCI subfamilies, Lhcr, Lhcq, and Lhcf, have been identified in coccolithophore PSI-EFCPI. The diversification and structure of coccolithophore EFCPIs are similar to those of diatom FCPIs (fig. S19). Furthermore, the Lhcr subfamily of haptophyte and diatom LHCIs cluster into an ochrophyte/haptophyte–specific group, suggesting their close evolutionary relationship (*58*). Phylogenetic analysis revealed a close evolutionary relationship between EFCPIs and FCPIs, and EFCPIs and FCPIs within the same clade shared similar structures (figs. S8 and S19). Specifically, EFCPI-3 and FCPI-3 shared similar structures and binding positions, substituting the nuclear-encoded PsaO subunit in the coccolithophore and diatom PSI cores, respectively (fig. S12E). EFCPI-1/6/7/8/10/11 share similar structures with their diatom counterparts, EFCPI-2/5/9 closely resemble FCPI-6/10, EFCPI-12/14/15/16/17/19/20/21 show structural similarities to FCPI-16, EFCPI-(22–35) exhibit similar structures to FCPI-21/23/24, and EFCPI-4/13/18 have structural features akin to FCPI-12/15/13, respectively (fig. S19B). These observations support the hypothesis that nuclear gene transfer occurred from ochrophytes to haptophytes and that haptophyte LHCIs were derived from ochrophyte LHCIs (Fig. 6). Together, our findings, along with previous genome analysis, suggest that the coccolithophore PSI core and LHCIs may have distinct origins, in agreement with recent structural studies of PSI-LHCI from coccolith-lacking haptophytes (*22*).

Coccolithophores originated from the calcification of haptophytes (*28*, *29*). Coccolithophore PSI-EFCPI inherited the structural features of PSI-LHCI from coccolith-lacking haptophytes, whereas it has 13 extra LHCIs and a distinct pigment composition. The extra EFCPI-(23–35) locate in the same clade as EFCPI-22 and share similar structures with EFCPI-22, suggesting that EFCPI-(23–35) may be derived from EFCPI-22. The extra EFCPI-(23–35) contain more Chls c and Cars, promoting the absorption of blue/green light and consequently facilitating the survival of coccolithophores in the low-light environment of deep ocean. In addition, the increased Cars and the specific hFx may boost photoprotection in the high-light environment of the surface ocean. Structural differentiation indicates the evolutionary adaptation of PSI-EFCPI to the ecological adaptation of coccolithophores after calcification.

In summary, our in-depth structural analysis of *E. huxleyi* PSI-EFCPI uncovered the specific compositions and arrangements of proteins and pigments within the coccolithophore PSI-LHCI supercomplex, representing a giant PSI-LHCI supercomplex. Notably, we identified numerous specific pigment-binding sites within the peripheral EFCPIs, particularly at subunit interfaces, which together form a unique pigment network that enhances the absorption of blue-green light. These characteristics facilitate coccolithophore photosynthesis in deep ocean layers, where blue-green wavelengths dominate the light environment. Moreover, the photoprotective Fx-hFx cycle coupled with the Ddx-Dtx cycle was shown to reduce EET, enhance energy dissipation, and improve coccolithophore survival under high-light conditions in the surface ocean. Collectively, these structural features provide insights into the adaptive strategies for light capture and energy transfer used by coccolithophore PSI-EFCPI within the protective environment of coccoliths. These adaptations contribute to the remarkable resilience and widespread distribution of coccolithophores in oceans.

## MATERIALS AND METHODS

### PSI-EFCPI purification

*E. huxleyi* was purchased from the Center for Collections of Marine Bacteria and Phytoplankton (Xiamen University, Xiamen, China) and was cultured in F/2 medium at 22°C under continuous illumination at 40 μmol photons m^−2^ s^−1^ with bubbling of air. A previous study showed that *E. huxleyi* achieves optimal growth at a light intensity of ~300 μmol photons m^−2^ s^−1^. The growth rate of *E. huxleyi* decreased to 30% at 40 μmol photons m^−2^ s^−1^ (*66*), which represents a relatively low light intensity for *E. huxleyi*. It is assumed that low light conditions may facilitate the binding of peripheral LHCIs around the PSI core, whereas high light could induce the dissociation of LHCIs from diatom PSI-LHCI to prevent photodamage (*26*). Therefore, we chose 40 μmol photons m^−2^ s^−1^ to preculture the cells, with the aim of capturing more structural information of the large peripheral antenna. The cells in the logarithmic growth phase were harvested by centrifugation at 4°C and 7000*g* for 15 min; washed twice with 30 ml of MES1 buffer containing 25 mM MES-NaOH (pH 6.5), 10 mM MgCl_2_, and 1 M betaine; and resuspended in 10 ml of MES1 buffer. The cells were then disrupted using glass beads with diameters of 212 to 300 μm. The crushed cells were centrifuged at 3000*g* for 2 min, and the supernatants were collected and centrifuged at 21,000*g* for 20 min to collect thylakoid membranes. The crude thylakoid membranes were washed with MES2 buffer (25 mM MES-NaOH, pH 6.5, 1.0 M betaine, and 1 mM EDTA) and resuspended in MES3 buffer (25 mM MES-NaOH, pH 6.5, 1.0 M betaine, 10 mM NaCl, and 5.0 mM CaCl_2_) at 0.3 mg ml^−1^ Chl. Dodecyl-α-d-maltopyranoside (α-DDM; from Anatrace, US) was added at a final concentration of 3.6% (w/v) and incubated on ice for 20 min (shaking every 5 min). The mixture was centrifuged at 21,000*g* for 20 min, and the supernatant was loaded onto a 10 to 30% discontinuous sucrose gradient containing 0.02% α-DDM in MES3 buffer with a gradient interval of 2%. The PSI-LHCI band was collected after centrifugation at 230,000*g* for 20 hours (Beckman SW41 Rotor) and further purified by gel filtration chromatography (GE; Superose 6 Increase 10/300 GL) in MES4 buffer containing 25 mM MES, pH 6.5, 0.5 M betaine, 50 mM NaCl, 5 mM CaCl2, and 0.02% α-DDM. The PSI-EFCPI supercomplexes were concentrated using a 100-kDa cutoff filter (Amicon Ultra; Millipore). The purification process was conducted under dim light at 4°C.

### Characterization of PSI-EFCPI

Room-temperature absorption spectra were measured using a spectrophotometer (UV-Vis 1900, Shimadzu). Purified proteins were denatured and separated using 8 to 16% SDS–polyacrylamide gel electrophoresis. Protein bands were extracted from the gels. After reduction with dithiothreitol and alkylation with iodoacetamide, the protein bands were digested with trypsin. The peptide fragments were analyzed by liquid chromatography–tandem mass spectrometry using an Easy-nLC 1000 System coupled to a Q Exactive mass spectrometer (Thermo Fisher Scientific). Peptides were separated using a phase trap column (nanoViper C18, 100 μm by 2 cm, Thermo Fisher Scientific) connected to a C18-reversed phase analytical column (75 μm by 10 cm, 3-μm resin, Thermo Fisher Scientific). The obtained data were analyzed using MaxQuant 1.6.14 software, and the acquired spectra were searched against the selected databases to determine qualitative identification information for the target protein-peptide molecules.

The pigment composition of PSI-EFCPI was analyzed using HPLC, as described previously (*22*). The pigments were extracted using precooled 90% acetone overnight at 4°C in the dark and then injected into a C18 reversed-phase column (250 by 4.6 mm, 5-μm particle size, Waters, Ireland) at a flow rate of 1 ml/min. Elutes were detected at 445 nm, with a wavelength detection range of 300 to 800 nm. Eight pigments, including Chl c, Fx, hFx, Ddx, Dtx, Chl c_2_-MGDG, Chl a, and β-Car, were identified on the basis of the characteristic absorption peaks and elution profiles, which is consistent with previous reports (*33*, *67*).

### Sequence analysis of PSI-EFCPI

Transcriptome sequencing of *E. huxleyi* was performed by Huada using high-throughput sequencing. Cells were collected by centrifugation at 4°C and 7000*g* for 15 min. Total RNA was extracted from the cells, and cDNA libraries were prepared. Double-stranded cDNAs were synthesized using mRNA, random hexamer primers, ribonuclease H, and DNA polymerase I. cDNA was subjected to terminal repair, A-tailing, and adapter integration for subsequent hybridization. cDNA fragments ~150 base pairs in length were selected using the AMPure XP system (Beckman Coulter, Beverly, US). cDNA was first treated with USER Enzyme (NEB) and then amplified using polymerase chain reaction to obtain the final cDNA library. High-throughput sequencing was performed using paired-end reads on a Huada DNBSEQ platform. Sequences of the PSI core and EFCPIs were determined by retrieving homologous sequences from the transcriptome. Sequence comparisons were performed using CLC Sequence Viewer 8.0 and ESPript 3.0. The software Mega X was used to construct the phylogenetic tree, and the sequences for producing the phylogenetic tree were aligned using MUSCLE with default parameters (*68*).

### Cryo-EM data collection and processing

The holey-carbon grid (Quantifoil Au R2/1, 200 mesh) was glow discharged, and then 4 μl of samples (2 mg ml^−1^) was applied to the freshly prepared grid for blotting on a Vitrobot Mark IV (Thermo Fisher Scientific). The blotting time was 2 s at 8°C and 100% humidity. Data acquisition was performed with a 300-kV Titan Krios G3i microscope (Thermo Fisher Scientific) that featured a K3 BioQuantum direct electron detector (Gatan Inc.) with a nominal magnification of ×81,000 (corresponding to a pixel size of 0.53 Å). In addition, 8089 movie stacks were recorded over a range of defocus parameters from −1.2 to −2.2 μm. A 20-eV energy filter slit (GIF, Gatan) was used with a total dose of 50 Å^−2^.

Images were processed primarily using cryoSPARC version 3.3.1 (*69*). First, patch motion correction and binning by a factor of 2 with dose weighting were performed on all movie stacks (*70*). The contrast transfer function (CTF) parameters for each movie were estimated using Patch CTF Estimation before automatic particle picking (*71*). A total of 120,073 particles were selected for ab initio reconstruction and three-dimensional (3D) classification after particle extraction and two rounds of reference-free 2D classifications. Subsequently, 93,213 particles were selected for homogeneous refinement. To improve the resolution of the density map, after 3D nonhomogeneous refinement and sharpening, global (per-group) CTF refinement, local (per-particle) CTF refinement, particle subtraction, and local refinement with a soft mask of the peripheral LHCI regions were performed. The overall resolution of the map was 3.1 Å according to the gold-standard FSC 0.143.

### Model building and refinement

The 3.1-Å resolution cryo-EM map was used to construct the model of *E. huxleyi* PSI-EFCPI. First, the *I. galbana* PSI-iFCPI structure [Protein Data Bank (PDB): 8Z11] was fitted to the map using ChimeraX (*72*). The homologous sequences of PSI-iFCPI obtained from the transcriptome and National Center for Biotechnology Information sequences of *E. huxleyi* were used to correct the amino acid residues of PSI core L_EFP_ and EFCPI-(1–22) by Coot (*73*, *74*). For EFCPI-(23–35), iFCPI-22 was fitted to the map. Potential sequences were searched by blasting the transcriptome sequences against the sequences of iFCPI-22. Then, each potential sequence was manually fitted to the cryo-EM map to identify the sequences matching to the cryo-EM map. Because of the resolution limitation of the density map, all Chl c molecules were identified as Chl c_2_, and the potential binding site for Dtx was designated as Ddx. Chl a and c molecules were distinguished as previously described (*18*, *40*). Chls a and c were distinguished by the density maps corresponding to the phytol chain for Chl a and the planarity of C-18^1^, C-18, C-17, and C-17^1^ resulting from the C-18═C-17 double bond for Chl c. This approach is generally reliable for distinguishing Chls a and c in the inner EFCPIs with a higher resolution. In these map regions, Chl c303 is conserved in most of EFCPIs, and Chl c304/c310/c317 are conserved in Lhcq-type EFCPIs. Thus, the Chl 303 site in peripheral EFCPIs with a lower resolution and the Chl 304/310/317 sites in peripheral Lhcq-type EFCPIs with a lower resolution were assigned as Chl c. For the other Chl sites in peripheral EFCPIs with a lower resolution, the Chl molecules that could not be unambiguously identified were tentatively assigned as Chl a. As the precise structure of Chl c_2_-MGDG remains unclear, the potential binding site for Chl c_2_-MGDG was tentatively assigned as one Chl c_2_ and one MGDG positioned in close proximity. Fx and Ddx molecules were discriminated on the basis of the density of the Car head group (*40*). hFx was identified on the basis of its hexanoyloxy tail. khFx shares a similar structure to hFx and is assigned as hFx because of limited density. Compared with hFx, the esterified tail of GyrE is located close to the end without the ester group. Densities of GyrE were identified in the map, and GyrE molecules were assigned. Geometrical restraints of pigments were generated by the Grade web server, and all residues and cofactors were manually adjusted by Coot. The constructed model was refined using the Phenix real-space refinement (*75*) and then corrected and adjusted manually using Coot. These processes were iterated to improve the quality of the final atomic model. The geometry of the structural model was evaluated using Phenix (table S1).

### Femtosecond TA spectroscopy

Femtosecond time-resolved TA measurements were performed using a Femto-TA100 spectrometer (Time-Tech Spectra, Beijing, China). A Ti:sapphire regenerative amplifier (Spitfire Ace, Spectra-Physics, US) produced pulses of ~70 fs at a central wavelength of 800 nm with a repetition rate of 5 kHz. The laser output was split by a beam splitter into pump and probe paths; one portion was used to generate a white-light continuum probe, while the other drove a tunable optical parametric amplifier (TOPAS, Light Conversion Ltd.) to produce 445-nm pump pulses. The pump pulse energy was ∼4 nJ per pulse with a spot size of ~500 μm on the sample. The pump and probe beams were linearly polarized at a magic angle (~54.7°) relative to each other to minimize the orientation-dependent (anisotropic) contributions. The samples were contained in 1-mm-path-length fused silica cuvettes and maintained an optical density of ~0.3 at 680 nm. To prevent photodamage, the cuvette was continuously translated perpendicularly to the beam path.

### Time-resolved fluorescence spectroscopy

For time-resolved fluorescence measurements, a femtosecond amplifier (Spitfire ACE, Spectra-Physics) generating 70-fs pulses at a repetition rate of 5 kHz was used. Excitation pulses at 445 nm were produced using a tunable optical parametric amplifier (TOPAS, Light Conversion Ltd.) driven by a femtosecond amplifier. The energy of the excitation pulses was adjusted to 2 nJ per pulse, with a ∼200-μm-diameter spot on the sample. All the samples were placed in 1-mm-thick fused silica cuvettes with an optical density of ~0.3 at 680 nm. A high-quality 500-nm long-pass filter was used to block the scattered excitation light. The emitted fluorescence was detected by a streak camera (model 5200, XIOPM, China), which operated at scanning ranges of 1.4 and 5 ns, with time intervals of 0.7 and 2.4 ps, respectively.

### Simulation of the EET in PSI-EFCPI

The time constants of EET within PSI-EFCPI were calculated in the limit of Förster theory (*51*). The excitations of the system are assumed to be described in terms of single-pigment excitations. Generalized Förster theory was used to calculate the energy transfer between the pigment clusters of the individual subunits. The energy transfer rate between a donor pigment cluster and an acceptor pigment cluster was calculated using this theory (*52*). The detailed process is described in our recent studies (*20*, *22*).

The calculation was performed using Gaussian16 software (Gaussian Inc., Wallingford, CT) and custom Python scripts (https://doi.org/10.5281/zenodo.10791187). In our model, we have treated all Chl molecules as distinct entities for subsequent calculation. CAM-B3LYP/6-31G* was used, and the keyword Iop (9/40 = 4) was included in the calculation to provide more detailed configuration coefficients for subsequent calculations of electronic coupling. The energy for the first excited state of the pigment was calculated using time-dependent density functional theory (*76*). For Chl a or c pairs, the site energies were calculated using time-dependent density functional theory to evaluate the spectral terms. While this approach is effective for various conformations of Chl a or c, it is less reliable for calculating the relative excited energy difference between Chls a and c. Consequently, we used site energy data, which are 663.5 nm for Chl a and 633.5 nm for Chl c, to compute the spectral overlap between Chls a and c.

## References

[1] N. Nelson, W. Junge, Structure and energy transfer in photosystems of oxygenic photosynthesis. Annu. Rev. Biochem. 84, 659–683 (2015). 25747397 10.1146/annurev-biochem-092914-041942

[2] R. Croce, H. van Amerongen, Natural strategies for photosynthetic light harvesting. Nat. Chem. Biol. 10, 492–501 (2014). 24937067 10.1038/nchembio.1555

[3] M. Iwai, D. Patel-Tupper, K. K. Niyogi, Structural diversity in eukaryotic photosynthetic light harvesting. Annu. Rev. Plant Biol. 75, 119–152 (2024). 38360524 10.1146/annurev-arplant-070623-015519

[4] X. Qin, X. Pi, W. Wang, G. Han, L. Zhu, M. Liu, L. Cheng, J. R. Shen, T. Kuang, S. F. Sui, Structure of a green algal photosystem I in complex with a large number of light-harvesting complex I subunits. Nat. Plants 5, 263–272 (2019). 30850820 10.1038/s41477-019-0379-y

[5] X. Su, J. Ma, X. Pan, X. Zhao, W. Chang, Z. Liu, X. Zhang, M. Li, Antenna arrangement and energy transfer pathways of a green algal photosystem-I-LHCI supercomplex. Nat. Plants 5, 273–281 (2019). 30850819 10.1038/s41477-019-0380-5

[6] I. Caspy, T. Malavath, D. Klaiman, M. Fadeeva, Y. Shkolnisky, N. Nelson, Structure and energy transfer pathways of the *Dunaliella Salina* photosystem I supercomplex. Biochim. Biophys. Acta Bioenerg. 1861, 148253 (2020). 32569661 10.1016/j.bbabio.2020.148253

[7] A. Ishii, J. Shan, X. Sheng, E. Kim, A. Watanabe, M. Yokono, C. Noda, C. Song, K. Murata, Z. Liu, J. Minagawa, The photosystem I supercomplex from a primordial green alga *Ostreococcus tauri* harbors three light-harvesting complex trimers. eLife 12, e84488 (2023). 36951548 10.7554/eLife.84488 PMC10097422

[8] M. Suga, S. I. Ozawa, K. Yoshida-Motomura, F. Akita, N. Miyazaki, Y. Takahashi, Structure of the green algal photosystem I supercomplex with a decameric light-harvesting complex I. Nat. Plants 5, 626–636 (2019). 31182847 10.1038/s41477-019-0438-4

[9] X. Qin, M. Suga, T. Kuang, J. R. Shen, Photosynthesis., Structural basis for energy transfer pathways in the plant PSI-LHCI supercomplex. Science 348, 989–995 (2015). 26023133 10.1126/science.aab0214

[10] X. Pan, J. Ma, X. Su, P. Cao, W. Chang, Z. Liu, X. Zhang, M. Li, Structure of the maize photosystem I supercomplex with light-harvesting complexes I and II. Science 360, 1109–1113 (2018). 29880686 10.1126/science.aat1156

[11] S. Zhang, K. L. Tang, Q. J. Yan, X. Y. Li, L. L. Shen, W. D. Wang, Y. K. He, T. Y. Kuang, G. Y. Han, J. R. Shen, X. Zhang, Structural insights into a unique PSI-LHCI-LHCII-Lhcb9 supercomplex from moss. Nat. Plants 9, 832–846 (2023). 37095225 10.1038/s41477-023-01401-4

[12] H. Y. Sun, H. Shang, X. W. Pan, M. Li, Structural insights into the assembly and energy transfer of the Lhcb9-dependent photosystem I from moss. Nat. Plants 9, 1347–1358 (2023). 37474782 10.1038/s41477-023-01463-4

[13] Q. Yan, L. Zhao, W. Wang, X. Pi, G. Han, J. Wang, L. Cheng, Y. K. He, T. Kuang, X. Qin, S. F. Sui, J. R. Shen, Antenna arrangement and energy-transfer pathways of PSI-LHCI from the moss *Physcomitrella patens*. Cell Discov. 7, 10 (2021). 33589616 10.1038/s41421-021-00242-9 PMC7884438

[14] C. Gorski, R. Riddle, H. Toporik, Z. Da, Z. Dobson, D. Williams, Y. Mazor, The structure of the Physcomitrium patens photosystem I reveals a unique Lhca2 paralogue replacing Lhca4. Nat. Plants 8, 307–316 (2022). 35190662 10.1038/s41477-022-01099-w

[15] X. You, X. Zhang, J. Cheng, Y. Xiao, J. Ma, S. Sun, X. Zhang, H. W. Wang, S. F. Sui, *In situ* structure of the red algal phycobilisome-PSII-PSI-LHC megacomplex. Nature 616, 199–206 (2023). 36922595 10.1038/s41586-023-05831-0

[16] X. Pi, L. Tian, H. E. Dai, X. Qin, L. Cheng, T. Kuang, S. F. Sui, J. R. Shen, Unique organization of photosystem I-light-harvesting supercomplex revealed by cryo-EM from a red alga. Proc. Natl. Acad. Sci. U.S.A. 115, 4423–4428 (2018). 29632169 10.1073/pnas.1722482115 PMC5924924

[17] K. Kato, T. Hamaguchi, M. Kumazawa, Y. Nakajima, K. Ifuku, S. Hirooka, Y. Hirose, S. Y. Miyagishima, T. Suzuki, K. Kawakami, N. Dohmae, K. Yonekura, J. R. Shen, R. Nagao, The structure of PSI-LHCI from *Cyanidium caldarium* provides evolutionary insights into conservation and diversity of red-lineage LHCs. Proc. Natl. Acad. Sci. U.S.A. 121, e2319658121 (2024). 38442179 10.1073/pnas.2319658121 PMC10945839

[18] L. S. Zhao, P. Wang, K. Li, Q. B. Zhang, F. Y. He, C. Y. Li, H. N. Su, X. L. Chen, L. N. Liu, Y. Z. Zhang, Structural basis and evolution of the photosystem I-light-harvesting supercomplex of cryptophyte algae. Plant Cell 35, 2449–2463 (2023). 36943796 10.1093/plcell/koad087 PMC10291030

[19] S. Zhang, L. Si, X. Su, X. Zhao, X. An, M. Li, Growth phase-dependent reorganization of cryptophyte photosystem I antennae. Commun. Biol. 7, 560 (2024). 38734819 10.1038/s42003-024-06268-5 PMC11088674

[20] L. S. Zhao, N. Wang, K. Li, C. Y. Li, J. P. Guo, F. Y. He, G. M. Liu, X. L. Chen, J. Gao, L. N. Liu, Y. Z. Zhang, Architecture of symbiotic dinoflagellate photosystem I-light-harvesting supercomplex in Symbiodinium. Nat. Commun. 15, 2392 (2024). 38493166 10.1038/s41467-024-46791-x PMC10944487

[21] X. Li, Z. Li, F. Wang, S. Zhao, C. Xu, Z. Mao, J. Duan, Y. Feng, Y. Yang, L. Shen, G. Wang, Y. Yang, L. J. Yu, M. Sang, G. Han, X. Wang, T. Kuang, J. R. Shen, W. Wang, Structures and organizations of PSI-AcpPCI supercomplexes from red tidal and coral symbiotic photosynthetic dinoflagellates. Proc. Natl. Acad. Sci. U.S.A. 121, e2315476121 (2024). 38319970 10.1073/pnas.2315476121 PMC10873603

[22] F. Y. He, L. S. Zhao, X. X. Qu, K. Li, J. P. Guo, F. Zhao, N. Wang, B. Y. Qin, X. L. Chen, J. Gao, L. N. Liu, Y. Z. Zhang, Structural insights into the assembly and energy transfer of haptophyte photosystem I-light- harvesting supercomplex. Proc. Natl. Acad. Sci. U.S.A. 121, e2413678121 (2024). 39642204 10.1073/pnas.2413678121 PMC11648859

[23] R. Shao, Y. Zou, H. Shang, Y. Qiu, Z. Liang, X. Su, S. Zhang, M. Li, X. Pan, Architecture of photosystem I-light-harvesting complex from the eukaryotic filamentous yellow-green alga *Tribonema minus*. J. Integr. Plant Biol. 67, 3014–3031 (2025). 40778523 10.1111/jipb.70010

[24] C. Xu, X. Pi, Y. Huang, G. Han, X. Chen, X. Qin, G. Huang, S. Zhao, Y. Yang, T. Kuang, W. Wang, S. F. Sui, J. R. Shen, Structural basis for energy transfer in a huge diatom PSI-FCPI supercomplex. Nat. Commun. 11, 5081 (2020). 33033236 10.1038/s41467-020-18867-x PMC7545214

[25] K. Kato, Y. Nakajima, J. Xing, M. Kumazawa, H. Ogawa, J. R. Shen, K. Ifuku, R. Nagao, Structural basis for molecular assembly of fucoxanthin chlorophyll *a*/*c*-binding proteins in a diatom photosystem I supercomplex. eLife 13, RP99858 (2024). 39480899 10.7554/eLife.99858 PMC11527431

[26] Y. Feng, Z. Li, Y. Yang, L. Shen, X. Li, X. Liu, X. Zhang, J. Zhang, F. Ren, Y. Wang, C. Liu, G. Han, X. Wang, T. Kuang, J. R. Shen, W. Wang, Structures of PSI-FCPI from *Thalassiosira pseudonana* grown under high light provide evidence for convergent evolution and light-adaptive strategies in diatom FCPIs. J. Integr. Plant Biol. 67, 949–966 (2025). 39670505 10.1111/jipb.13816

[27] F. M. Monteiro, L. T. Bach, C. Brownlee, P. Bown, R. E. Rickaby, A. J. Poulton, T. Tyrrell, L. Beaufort, S. Dutkiewicz, S. Gibbs, M. A. Gutowska, R. Lee, U. Riebesell, J. Young, A. Ridgwell, Why marine phytoplankton calcify. Sci. Adv. 2, e1501822 (2016). 27453937 10.1126/sciadv.1501822 PMC4956192

[28] P. R. Bown, J. A. Lees, J. R. Young, “Calcareous nannoplankton evolution and diversity through time” in *Coccolithophores: From Molecular Processes to Global Impacts*, J. R. Young, H. R. Thierstein, Ed. (Springer Berlin Heidelberg, Berlin, 2004), pp. 481–508.

[29] L. K. Medlin, A. G. Sáez, J. R. Young, A molecular clock for coccolithophores and implications for selectivity of phytoplankton extinctions across the K/T boundary. Mar. Micropaleontol. 67, 69–86 (2008).

[30] A. J. Poulton, T. R. Adey, W. M. Balch, P. M. Holligan, Relating coccolithophore calcification rates to phytoplankton community dynamics: regional differences and implications for carbon export. Deep Res. Part II Top Stud. Oceanogr. 54, 538–557 (2007).

[31] Y. Mizukawa, Y. Miyashita, M. Satoh, Y. Shiraiwa, M. Iwasaka, Light intensity modulation by coccoliths of *Emiliania huxleyi* as a micro-photo-regulator. Sci. Rep. 5, 13577 (2015). 26323524 10.1038/srep13577 PMC4555034

[32] R. Quintero-Torres, J. L. Aragon, M. Torres, M. Estrada, L. Cros, Strong far-field coherent scattering of ultraviolet radiation by holococcolithophores. Phys. Rev. E. Stat. Nonlin. Soft Matter Phys. 74, 032901 (2006). 17025685 10.1103/PhysRevE.74.032901

[33] J. L. Garrido, C. Brunet, F. Rodriguez, Pigment variations in *Emiliania huxleyi* (CCMP370) as a response to changes in light intensity or quality. Environ. Microbiol. 18, 4412–4425 (2016). 27198623 10.1111/1462-2920.13373

[34] G. L. Wheeler, D. Sturm, G. Langer, Gephyrocapsa huxleyi (*Emiliania huxleyi*) as a model system for coccolithophore biology. J. Phycol. 59, 1123–1129 (2023). 37983837 10.1111/jpy.13404

[35] E. Paasche, A review of the coccolithophorid *Emiliania huxleyi* (Prymnesiophyceae), with particular reference to growth coccolith formation and calcification-photosynthesis interactions. Phycologia 40, 503–529 (2001).

[36] H. Staleva-Musto, R. West, M. Trathnigg, D. Bina, R. Litvin, T. Polivka, Carotenoid-chlorophyll energy transfer in the fucoxanthin-chlorophyll complex binding a fucoxanthin acyloxy derivative. Faraday Discuss. 216, 460–475 (2019). 31012452 10.1039/c8fd00193f

[37] L. Shen, F. Ren, Y. C. Wang, Z. Li, M. Zheng, X. Li, W. Fan, Y. Yang, M. Sang, C. Liu, G. Han, S. Qin, J. Fan, L. Tian, T. Kuang, J. R. Shen, W. Wang, Structure and function of a huge photosystem I-fucoxanthin chlorophyll supercomplex from a coccolithophore. Science 389, eadv2132 (2025). 40934304 10.1126/science.adv2132

[38] R. Pettersen, G. Johnsen, J. Berge, E. K. Hovland, Phytoplankton chemotaxonomy in waters around the Svalbard archipelago reveals high amounts of Chl b and presence of gyroxanthin-diester. Polar Biol. 34, 627–635 (2011).

[39] W. Stolte, G. W. Kraay, A. A. M. Noordeloos, R. Riegman, Genetic and physiological variation in pigment composition of *Emiliania Huxleyi* (Prymnesiophyceae) and the potential use of its pigment ratios as a quantitative physiological marker. J. Phycol. 36, 529–539 (2000). 29544012 10.1046/j.1529-8817.2000.99158.x

[40] R. Nagao, K. Kato, K. Ifuku, T. Suzuki, M. Kumazawa, I. Uchiyama, Y. Kashino, N. Dohmae, S. Akimoto, J. R. Shen, N. Miyazaki, F. Akita, Structural basis for assembly and function of a diatom photosystem I-light-harvesting supercomplex. Nat. Commun. 11, 2481 (2020). 32424145 10.1038/s41467-020-16324-3 PMC7235021

[41] M. Zapata, S. W. Jeffrey, S. W. Wright, F. Rodríguez, J. L. Garrido, L. Clementson, Photosynthetic pigments in 37 species (65 strains) of Haptophyta: Implications for oceanography and chemotaxonomy. Mar. Ecol. Prog. Ser. 270, 83–102 (2004).

[42] J. L. Garrido, J. Otero, M. A. Maestro, M. Zapata, The main nonpolar chlorophyll c from *Emiliania huxleyi* (prymnesiophyceae) is a chlorophyll c_2_-monogalactosyldiacylglyceride ester: a mass spectrometry study. J. Phycol. 36, 497–505 (2000). 29544015 10.1046/j.1529-8817.2000.99135.x

[43] J. K. Hoober, L. L. Eggink, A potential role of chlorophylls b and c in assembly of light-harvesting complexes. FEBS Lett. 489, 1–3 (2001). 11231002 10.1016/s0014-5793(00)02410-8

[44] R. Nagao, M. Yokono, S. Akimoto, T. Tomo, High excitation energy quenching in fucoxanthin chlorophyll a/c-binding protein complexes from the diatom *Chaetoceros gracilis*. J. Phys. Chem. B 117, 6888–6895 (2013). 23688343 10.1021/jp403923q

[45] R. Goss, B. Lepetit, Biodiversity of NPQ. J. Plant Physiol. 172, 13–32 (2015). 24854581 10.1016/j.jplph.2014.03.004

[46] T. Lacour, E. Robert, J. Lavaud, Sustained xanthophyll pigments-related photoprotective NPQ is involved in photoinhibition in the haptophyte *Tisochrysis lutea*. Sci. Rep. 13, 14694 (2023). 37679420 10.1038/s41598-023-40298-z PMC10484918

[47] R. G. West, M. Fuciman, H. Staleva-Musto, V. Sebelik, D. Bina, M. Durchan, V. Kuznetsova, T. Polivka, Equilibration dependence of fucoxanthin S_1_ and ICT signatures on polarity, proticity, and temperature by multipulse femtosecond absorption spectroscopy. J. Phys. Chem. B 122, 7264–7276 (2018). 29963865 10.1021/acs.jpcb.8b04217

[48] G. N. Harris, D. J. Scanlan, R. J. Geider, Acclimation of *Emiliania huxleyi* (Prymnesiophyceae) to photon flux density. J. Phycol. 41, 851–862 (2005).

[49] R. Nagao, M. Yokono, T. Tomo, S. Akimoto, Control mechanism of excitation energy transfer in a complex consisting of photosystem II and fucoxanthin chlorophyll-binding protein. J. Phys. Chem. Lett. 5, 2983–2987 (2014). 26278247 10.1021/jz501496p

[50] J. S. Connolly, A. F. Janzen, E. B. Samuel, Fluorescence lifetimes of chlorophyll a—Solvent, concentration and oxygen dependence. Photochem. Photobiol. 36, 559–563 (1982).

[51] M. Sener, J. Strumpfer, J. Hsin, D. Chandler, S. Scheuring, C. N. Hunter, K. Schulten, Forster energy transfer theory as reflected in the structures of photosynthetic light-harvesting systems. Chemphyschem 12, 518–531 (2011). 21344591 10.1002/cphc.201000944 PMC3098534

[52] J. Hsin, J. Struempfer, M. Sener, P. Qian, C. N. Hunter, K. Schulten, Energy transfer dynamics in an RC-LH1-PufX tubular photosynthetic membrane. New J. Phys. 12, 085005 (2010). 10.1088/1367-2630/12/8/085005 PMC2997751 21152381

[53] J. I. Kim, C. E. Moore, J. M. Archibald, D. Bhattacharya, G. Yi, H. S. Yoon, W. Shin, Evolutionary dynamics of cryptophyte plastid genomes. Genome Biol. Evol. 9, 1859–1872 (2017). 28854597 10.1093/gbe/evx123 PMC5534331

[54] V. Zimorski, C. Ku, W. F. Martin, S. B. Gould, Endosymbiotic theory for organelle origins. Curr. Opin. Microbiol. 22, 38–48 (2014). 25306530 10.1016/j.mib.2014.09.008

[55] S. B. Gould, U. G. Maier, W. F. Martin, Protein import and the origin of red complex plastids. Curr. Biol. 25, R515–R521 (2015). 26079086 10.1016/j.cub.2015.04.033

[56] J. Petersen, A. K. Ludewig, V. Michael, B. Bunk, M. Jarek, D. Baurain, H. Brinkmann, Chromera velia, endosymbioses and the rhodoplex hypothesis—Plastid evolution in cryptophytes, alveolates, stramenopiles, and haptophytes (CASH lineages). Genome Biol. Evol. 6, 666–684 (2014). 24572015 10.1093/gbe/evu043 PMC3971594

[57] J. W. Stiller, J. Schreiber, J. Yue, H. Guo, Q. Ding, J. Huang, The evolution of photosynthesis in chromist algae through serial endosymbioses. Nat. Commun. 5, 5764 (2014). 25493338 10.1038/ncomms6764 PMC4284659

[58] M. Kumazawa, K. Ifuku, Unraveling the evolutionary trajectory of LHCI in red-lineage algae: Conservation, diversification, and neolocalization. iScience 27, 110897 (2024). 39386759 10.1016/j.isci.2024.110897 PMC11462038

[59] Y. Z. Zhang, K. Li, B. Y. Qin, J. P. Guo, Q. B. Zhang, D. L. Zhao, X. L. Chen, J. Gao, L. N. Liu, L. S. Zhao, Structure of cryptophyte photosystem II-light-harvesting antennae supercomplex. Nat. Commun. 15, 4999 (2024). 38866834 10.1038/s41467-024-49453-0 PMC11169493

[60] Z. Y. Mao, X. Y. Li, Z. H. Li, L. L. Shen, X. Y. Li, Y. Y. Yang, W. D. Wang, T. Y. Kuang, J. R. Shen, G. Y. Han, Structure and distinct supramolecular organization of a PSII-ACPII dimer from a cryptophyte alga *Chroomonas placoidea*. Nat. Commun. 15, 4535 (2024). 38806516 10.1038/s41467-024-48878-x PMC11133340

[61] A. Bodyl, K. Moszczynski, Did the peridinin plastid evolve through tertiary endosymbiosis? A hypothesis. Eur. J. Phycol. 41, 435–448 (2006).

[62] H. S. Yoon, J. D. Hackett, F. M. Van Dolah, T. Nosenko, L. Lidie, D. Bhattacharya, Tertiary endosymbiosis driven genome evolution in dinoflagellate algae. Mol. Biol. Evol. 22, 1299–1308 (2005). 15746017 10.1093/molbev/msi118

[63] T. Sevcíková, A. Horák, V. Klimes, V. Zbránková, E. Demir-Hilton, S. Sudek, J. Jenkins, J. Schmutz, P. Pribyl, J. Fousek, C. Vlcek, B. F. Lang, M. Oborník, A. Z. Worden, M. Eliás, Updating algal evolutionary relationships through plastid genome sequencing: Did alveolate plastids emerge through endosymbiosis of an ochrophyte? Sci. Rep. 5, 10134 (2015). 26017773 10.1038/srep10134 PMC4603697

[64] D. W. Rice, J. D. Palmer, An exceptional horizontal gene transfer in plastids: Gene replacement by a distant bacterial paralog and evidence that haptophyte and cryptophyte plastids are sisters. BMC Biol. 4, 31 (2006). 16956407 10.1186/1741-7007-4-31 PMC1570145

[65] A. Bodyl, J. W. Stiller, P. Mackiewicz, Chromalveolate plastids: Direct descent or multiple endosymbioses? Trends Ecol. Evol. 24, 119–121 (2009). 19200617 10.1016/j.tree.2008.11.003

[66] E. Paasche, Reduced coccolith calcite production under light-limited growth: A comparative study of three clones of *Emiliania huxleyi* (Prymnesiophyceae). Phycologia 38, 508–516 (1999).

[67] N. Leonardos, G. N. Harris, Comparative effects of light on pigments of two strains of *Emiliania huxleyi* (Haptophyta). J. Phycol. 42, 1217–1224 (2006).

[68] S. Kumar, G. Stecher, M. Li, C. Knyaz, K. Tamura, MEGA X: Molecular evolutionary genetics analysis across computing platforms. Mol. Biol. Evol. 35, 1547–1549 (2018). 29722887 10.1093/molbev/msy096 PMC5967553

[69] A. Punjani, J. L. Rubinstein, D. J. Fleet, M. A. Brubaker, cryoSPARC: Algorithms for rapid unsupervised cryo-EM structure determination. Nat. Methods 14, 290–296 (2017). 28165473 10.1038/nmeth.4169

[70] S. Q. Zheng, E. Palovcak, J. P. Armache, K. A. Verba, Y. Cheng, D. A. Agard, MotionCor2: Anisotropic correction of beam-induced motion for improved cryo-electron microscopy. Nat. Methods 14, 331–332 (2017). 28250466 10.1038/nmeth.4193 PMC5494038

[71] A. Rohou, N. Grigorieff, CTFFIND4: Fast and accurate defocus estimation from electron micrographs. J. Struct. Biol. 192, 216–221 (2015). 26278980 10.1016/j.jsb.2015.08.008 PMC6760662

[72] E. F. Pettersen, T. D. Goddard, C. C. Huang, G. S. Couch, D. M. Greenblatt, E. C. Meng, T. E. Ferrin, UCSF Chimera—A visualization system for exploratory research and analysis. J. Comput. Chem. 25, 1605–1612 (2004). 15264254 10.1002/jcc.20084

[73] A. Casanal, B. Lohkamp, P. Emsley, Current developments in Coot for macromolecular model building of electron cryo-microscopy and crystallographic data. Protein Sci. 29, 1069–1078 (2020). 31730249 10.1002/pro.3791 PMC7096722

[74] P. Emsley, B. Lohkamp, W. G. Scott, K. Cowtan, Features and development of Coot. Acta Crystallogr. D. Biol. Crystallogr. 66, 486–501 (2010). 20383002 10.1107/S0907444910007493 PMC2852313

[75] P. D. Adams, P. V. Afonine, G. Bunkoczi, V. B. Chen, I. W. Davis, N. Echols, J. J. Headd, L. W. Hung, G. J. Kapral, R. W. Grosse-Kunstleve, A. J. McCoy, N. W. Moriarty, R. Oeffner, R. J. Read, D. C. Richardson, J. S. Richardson, T. C. Terwilliger, P. H. Zwart, PHENIX: A comprehensive Python-based system for macromolecular structure solution. Acta Crystallogr. D. Biol. Crystallogr. 66, 213–221 (2010). 20124702 10.1107/S0907444909052925 PMC2815670

[76] E. Runge, E. K. U. Gross, Density-functional theory for time-dependent systems. Phys. Rev. Lett. 52, 997–1000 (1984).

