# Supplementary material for: Architecture and energy transfer of coccolithophore photosystem I with a huge light-harvesting antenna system
Source: Sci Adv. 2025 Dec 19;11(51):eaea4965. doi: 10.1126/sciadv.aea4965 (PMC12716418; doi:10.1126/sciadv.aea4965)
Supplement: Supplementary file 1 — Figs. S1 to S19 Tables S1 to S4 Legend for movie S1 [file sciadv.aea4965_sm.pdf]

Science Advances
AAAS

Supplementary Materials for

Architecture and energy transfer of coccolithophore photosystem I with a huge light-harvesting antenna system

Xiao-Meng Sun *et al.*

Corresponding author: Yu-Xiang Weng, yxweng@iphy.ac.cn; Jun Gao, gaojun@mail.hzau.edu.cn; Lu-Ning Liu, luning.liu@liverpool.ac.uk; Yu-Zhong Zhang, zhangyz@sdu.edu.cn; Long-Sheng Zhao, zhaols@sdu.edu.cn

Sci. Adv. **11**, eaea4965 (2025)
DOI: 10.1126/sciadv.aea4965

The PDF file includes:

Figs. S1 to S19
Tables S1 to S4
Legend for movie S1

Other Supplementary Material for this manuscript includes the following:

Movie S1

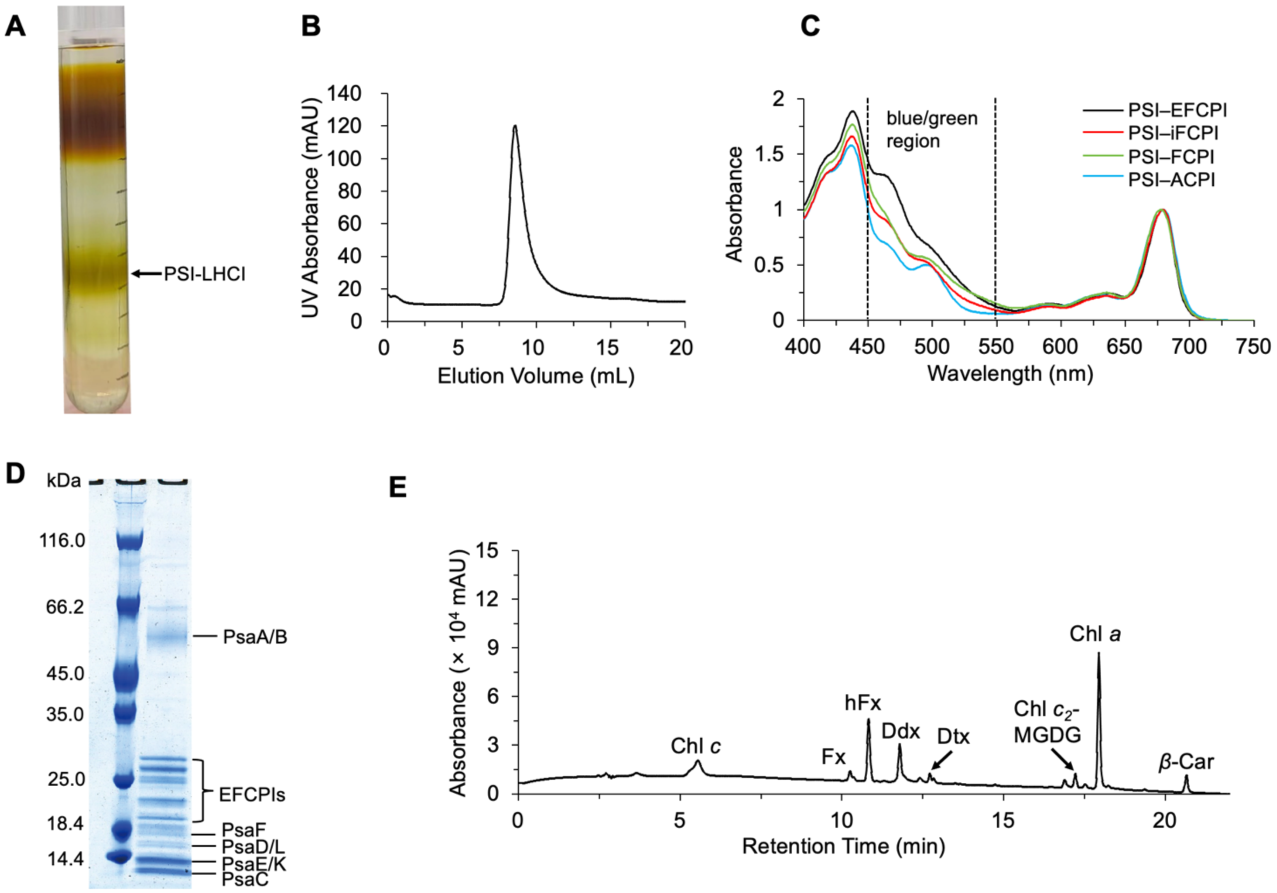

Fig. S1. Preparation and characterization of PSI–EFCPI supercomplex.

(**A**) Isolation of the PSI–EFCPI supercomplexes by ultracentrifugation using sucrose density gradient.
(**B**) Purification of the PSI–EFCPI supercomplexes by size-exclusion chromatography.
(**C**) Room-temperature absorption spectra of the PSI–EFCPI supercomplex, cryptophyte (*Chroomonas placoidea*) PSI–ACPI, diatom (*Chaetoceros gracilis*) PSI–FCPI and haptophyte (*Isochrysis galbana*) PSI–iFCPI supercomplexes. The blue and green light region was highlighted.
(**D**) SDS-PAGE analysis of the PSI–EFCPI supercomplex. The protein composition of the bands was indicated based on the mass spectrometry analysis.
(**E**) Pigment analysis of PSI–EFCPI by high performance liquid chromatography (HPLC), recorded at 445 nm. Based on the characteristic absorption spectrum of each peak fraction, eight major pigment peaks were identified as chlorophyll *c* (Chl *c*), fucoxanthin (Fx), 19'-hexanoyloxyfucoxanthin (hFx), diadinoxanthin (Ddx), diatoxanthin (Dtx), chlorophyll c_2-monogalactosyldiacylglycerol (Chl c_2-MGDG), chlorophyll *a* (Chl *a*) and β-carotene (β-Car) respectively.
These experiments were performed with more than three biological replicates, and the same results were obtained reproducibly.

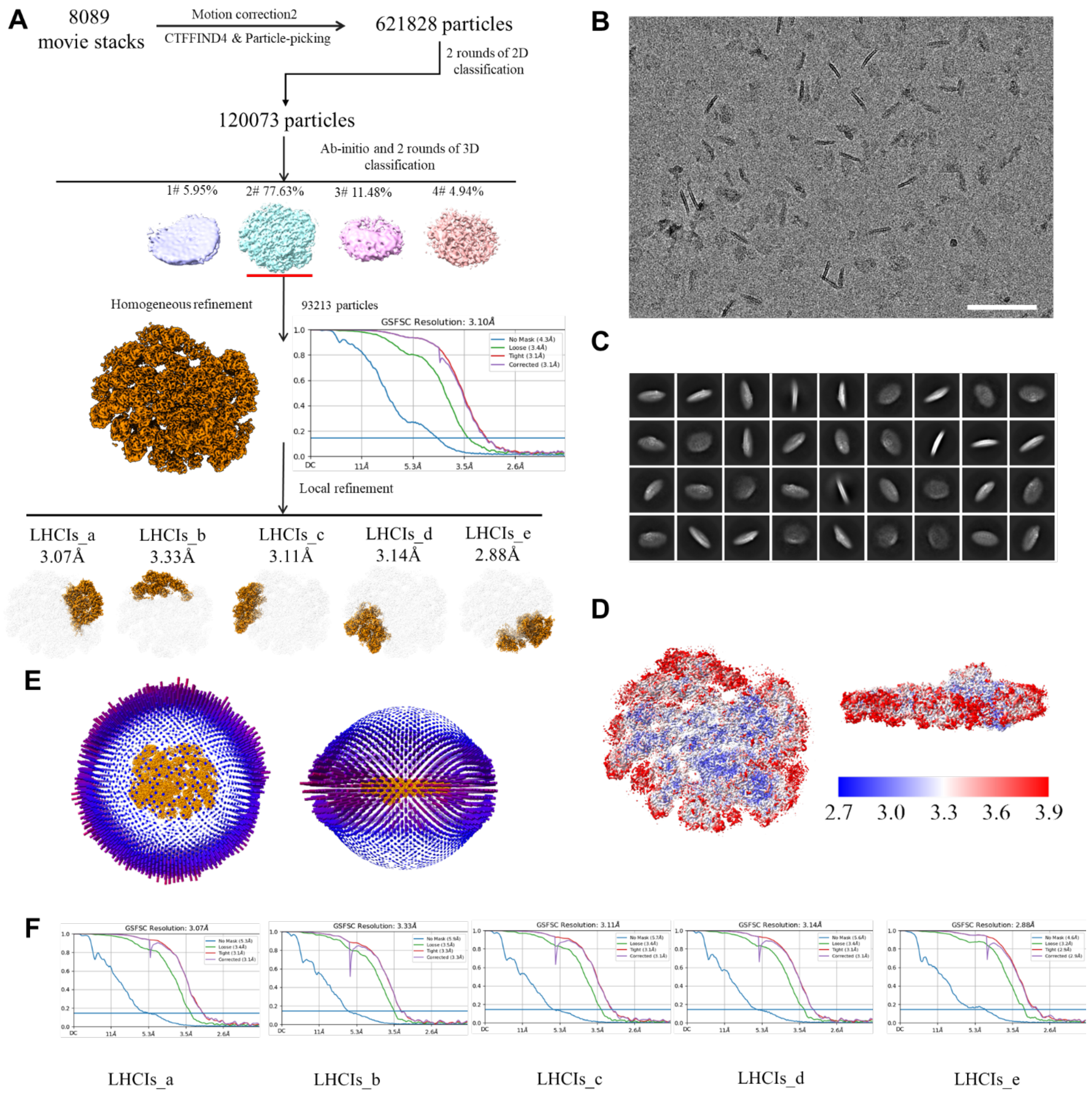

Fig. S2. Cryo-EM data collection and processing for the PSI–EFCPI supercomplex.

(**A**) Schematic flowchart for the cryo-EM data collection and processing.

(**B**) A typical cryo-EM micrograph of the PSI–EFCPI supercomplex. Scale bar, 100 nm.

(**C**) 2D classes of the PSI–EFCPI supercomplex. The box size is 530 Å.

(**D**) Local resolution distributions of the cryo-EM map estimated by ResMap.

(**E**) Angular distribution of particles used for reconstruction of the final density map.

(**F**) The gold standard Fourier shell correlation (FSC) curves for estimation of the resolution of the overall density map and local density map with criterion of 0.143.

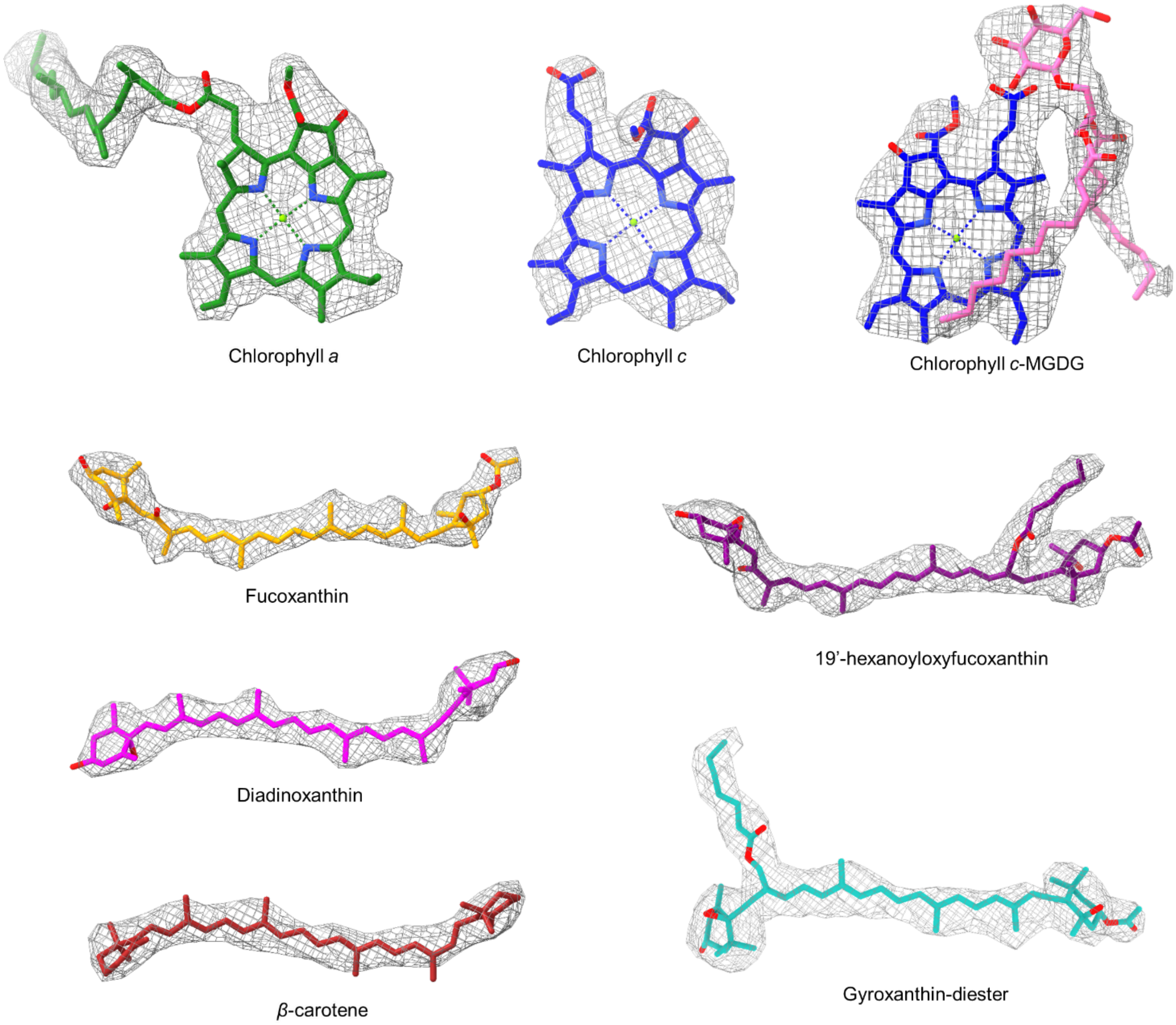

Fig. S3. Cryo-EM density maps and structures of pigment molecules in coccolithophore PSI–EFCPI supercomplex.

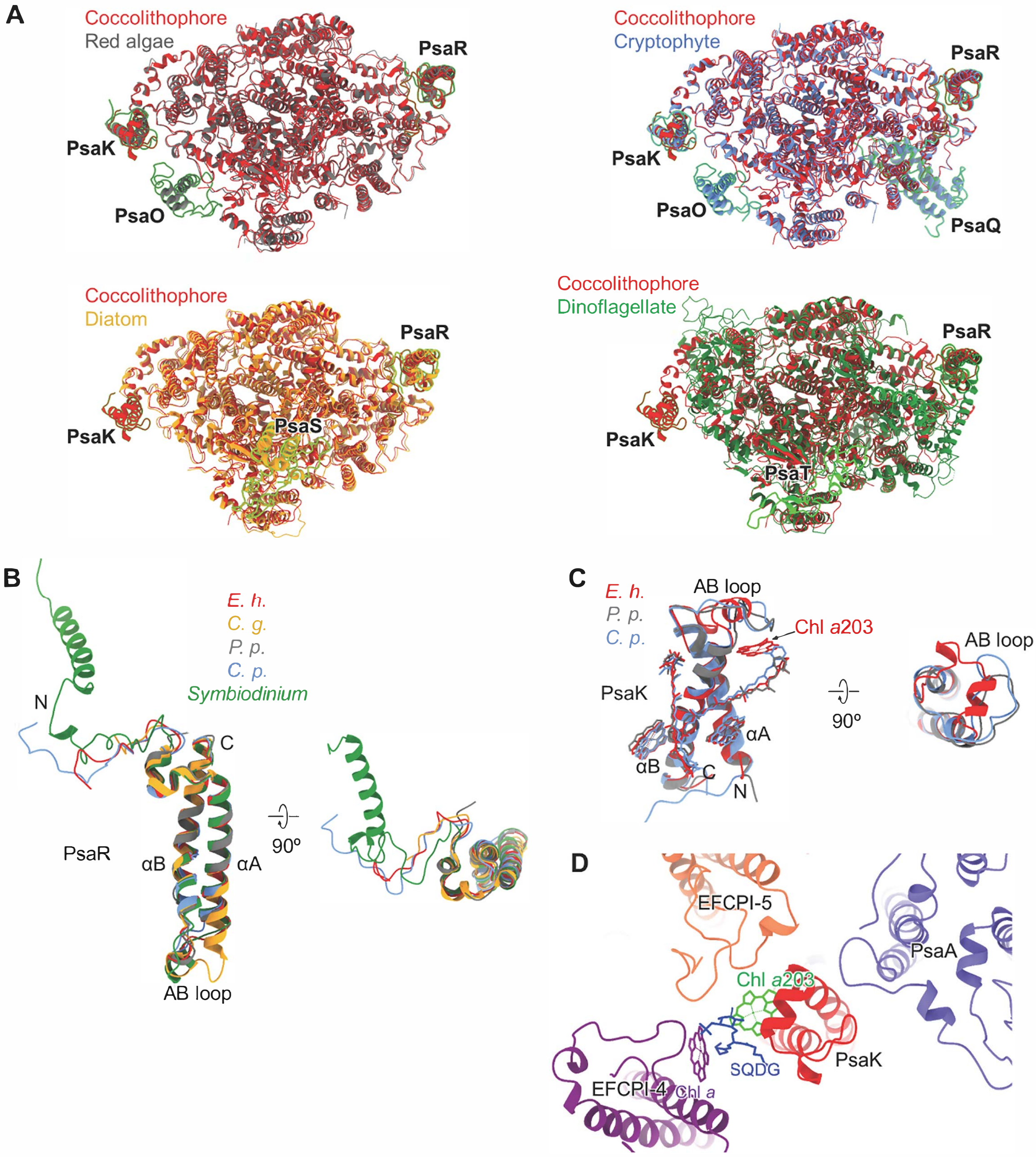
A
Coccolithophore
Red algae
PsaR
PsaK
PsaO
Coccolithophore
Cryptophyte
PsaR
PsaK
PsaO
PsaQ
Coccolithophore
Diatom
PsaR
PsaK
PsaS
Coccolithophore
Dinoflagellate
PsaR
PsaK
PsaT
B
E. h.
C. g.
P. p.
C. p.
Symbiodinium
N
C
PsaR
αB
αA
90º
AB loop
C
E. h.
P. p.
C. p.
AB loop
Chl a203
PsaK
αA
αB
C
N
90º
AB loop
D
EFCPI-5
PsaA
Chl a203
PsaK
SQDG
EFCPI-4
Chl a

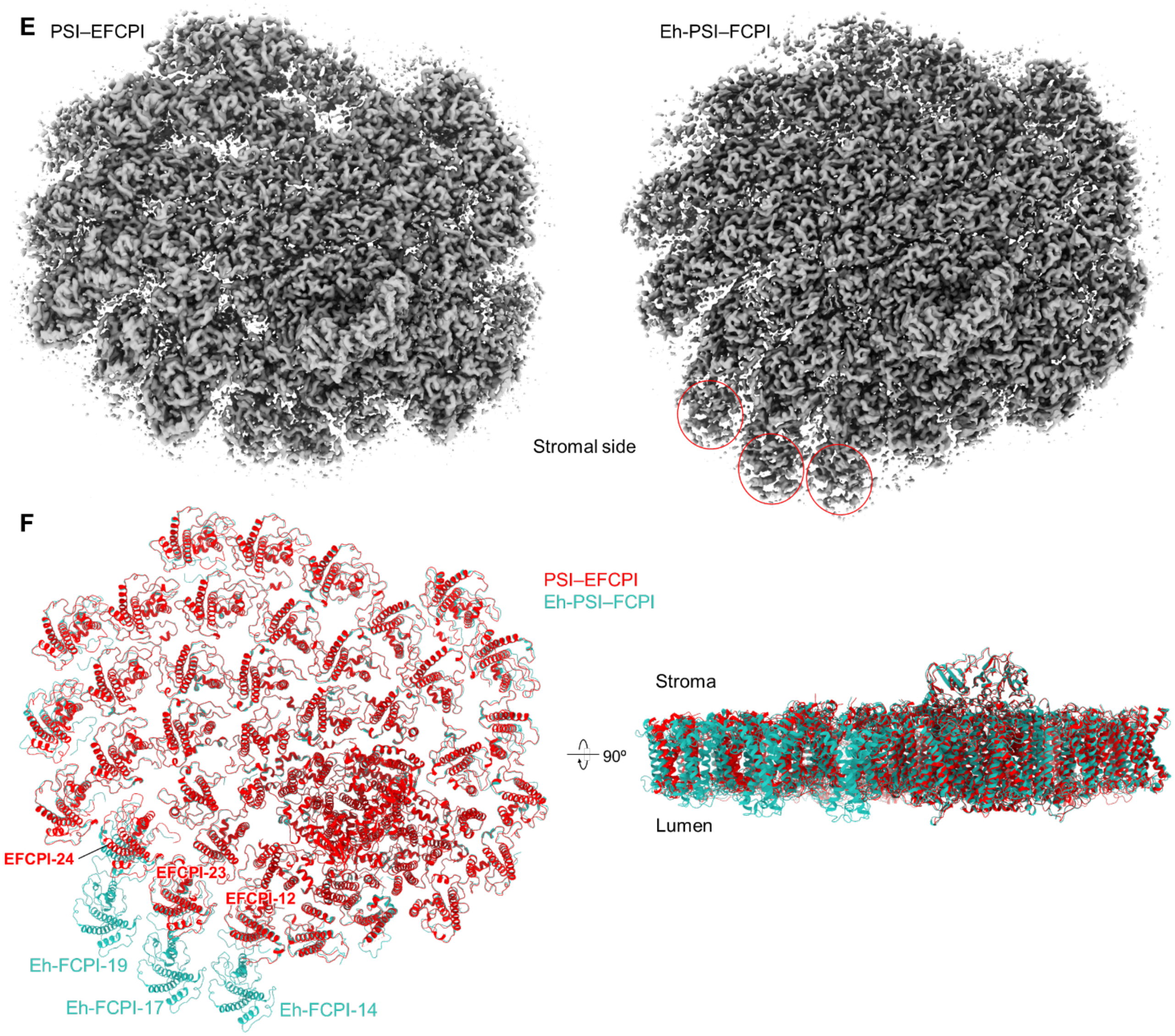

Fig. S4. Comparison of the coccolithophore PSI core with those of red alga, cryptophyte, diatom, dinoflagellate.

(**A**) Superposition of the coccolithophore PSI core (red) with red algal PSI core (gray, PDB: 7Y5E), cryptophyte PSI core (blue, PDB: 7Y7B), diatom PSI core (orange, PDB: 6LY5), and dinoflagellate PSI core (green, PDB: 8JJR). PsaK, PsaO, PsaQ, PsaR, PsaS, and PsaT are indicated.

(**B**) Comparison of the structure of PsaR in coccolithophore *E. huxleyi* (*E. h.*) PsaR (red), diatom *Chaetoceros gracilis* (*C. g.*) PsaR (orange), red algae *Porphyridium purpureum* (*P. p.*) PsaR (grey), cryptophyte *Chroomonas placoidea* (*C. p.*) PsaR (blue), and dinoflagellate *Symbiodinium* PsaR (green). The N-terminal loops, C-terminal loops, and AB loops between αA and αB are indicated.

(**C**) Comparison of the structure of PsaK in coccolithophore *E. huxleyi* (*E. h.*) PsaK (red), red algae *Porphyridium purpureum* (*P. p.*) PsaK (grey), and cryptophyte *Chroomonas placoidea* (*C. p.*) PsaK (blue). The N-terminal loops, C-terminal loops, and AB loops between αA and αB are indicated.

(**D**) The locations of Chl *a*203 and SQDG in PsaK viewed from the stromal side and its interactions with iFCPI-4/5.

(**E**) Density maps of reported Eh-PSI–FCPI from *E. huxleyi* and our PSI–EFCPI from *E. huxleyi*. Red circles indicate the extra antennas.

(**F**) Superposition of the reported Eh-PSI–FCPI (sea green, PDB: 9JJ8) and our PSI–EFCPI (red).

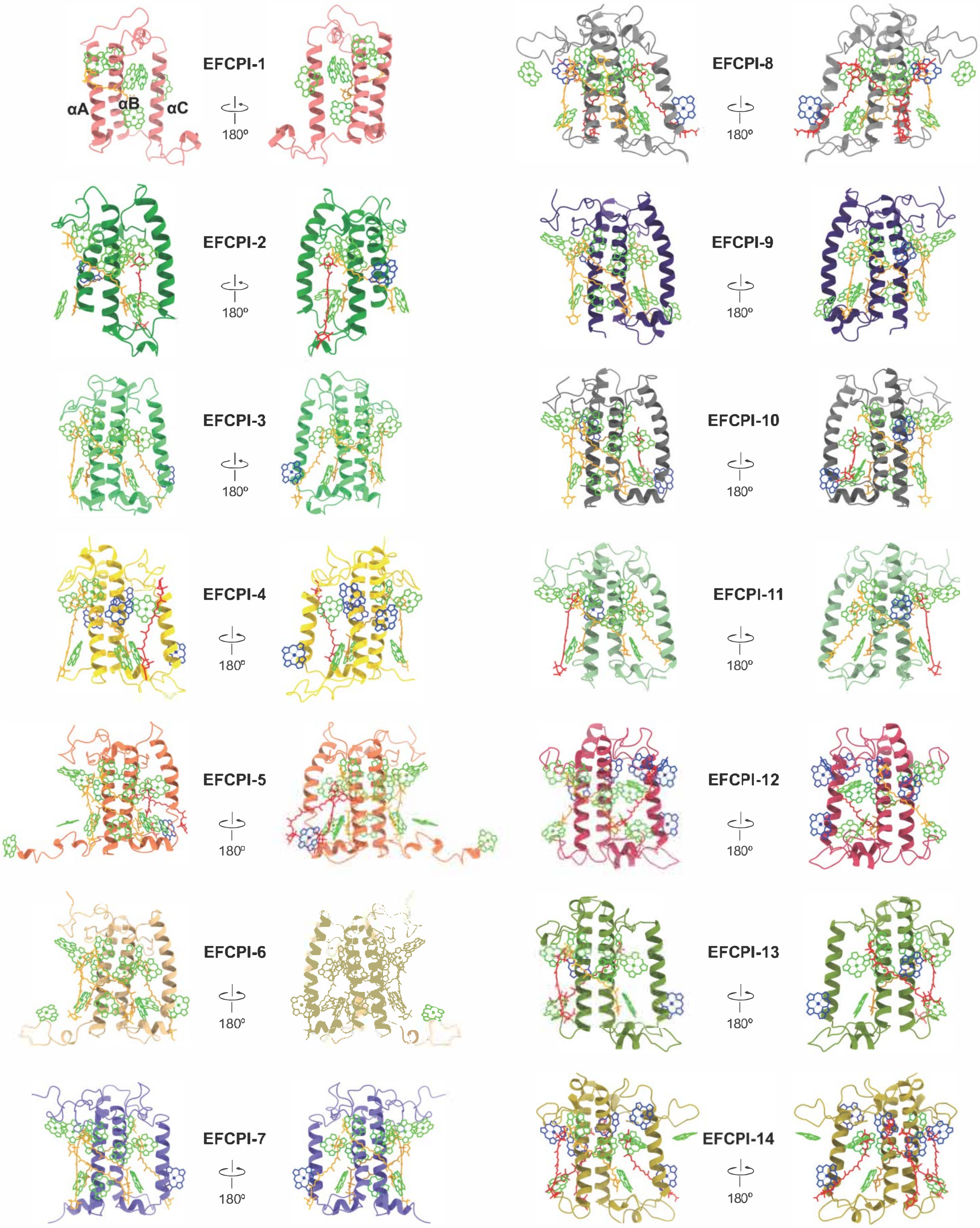

αA
αB
αC
EFCPI-1
180º
EFCPI-2
180º
EFCPI-3
180º
EFCPI-4
180º
EFCPI-5
180º
EFCPI-6
180º
EFCPI-7
180º
EFCPI-8
180º
EFCPI-9
180º
EFCPI-10
180º
EFCPI-11
180º
EFCPI-12
180º
EFCPI-13
180º
EFCPI-14
180º

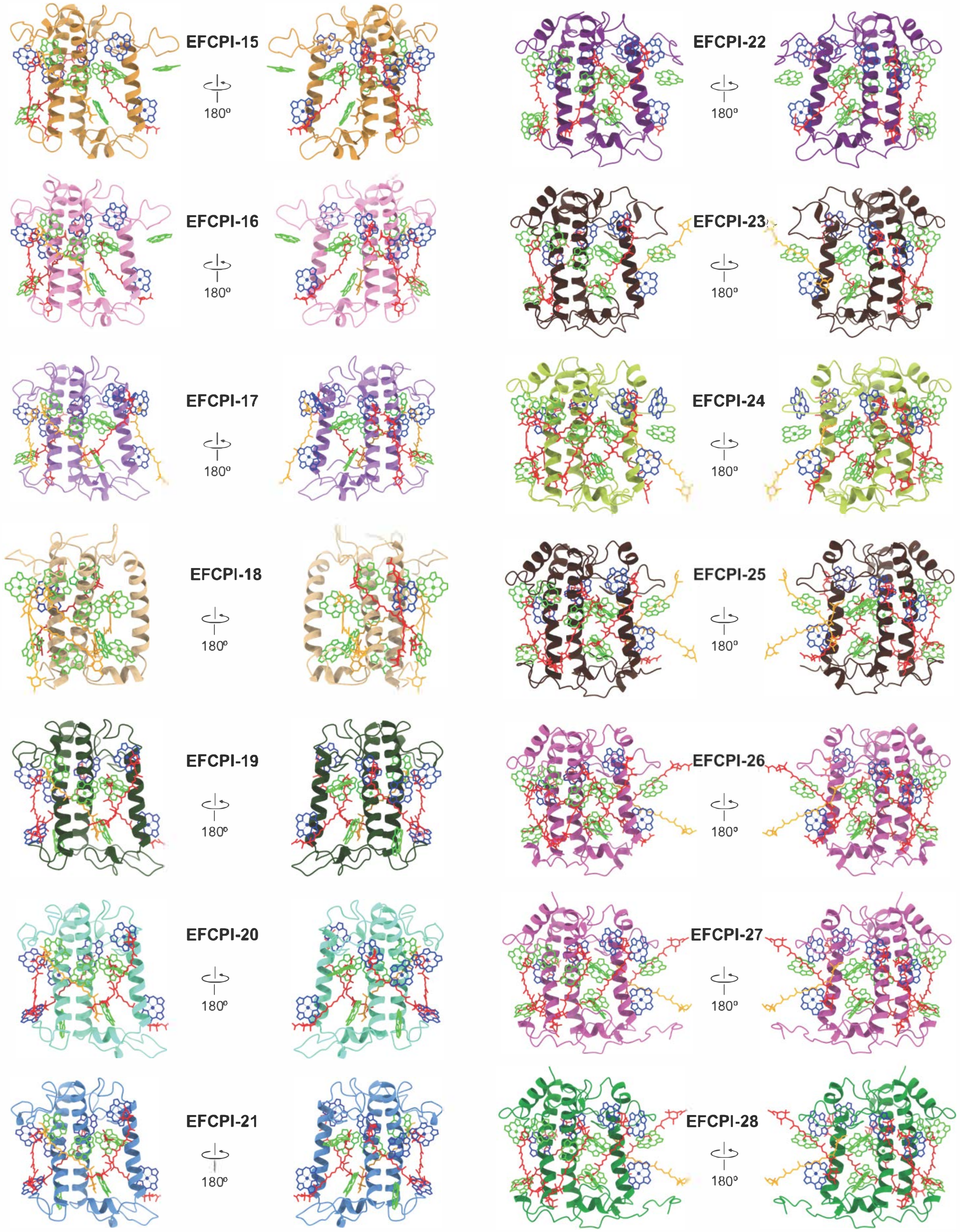
EFCPI-15
180º
EFCPI-16
180º
EFCPI-17
180º
EFCPI-18
180º
EFCPI-19
180º
EFCPI-20
180º
EFCPI-21
180º
EFCPI-22
180º
EFCPI-23
180º
EFCPI-24
180º
EFCPI-25
180º
EFCPI-26
180º
EFCPI-27
180º
EFCPI-28
180º

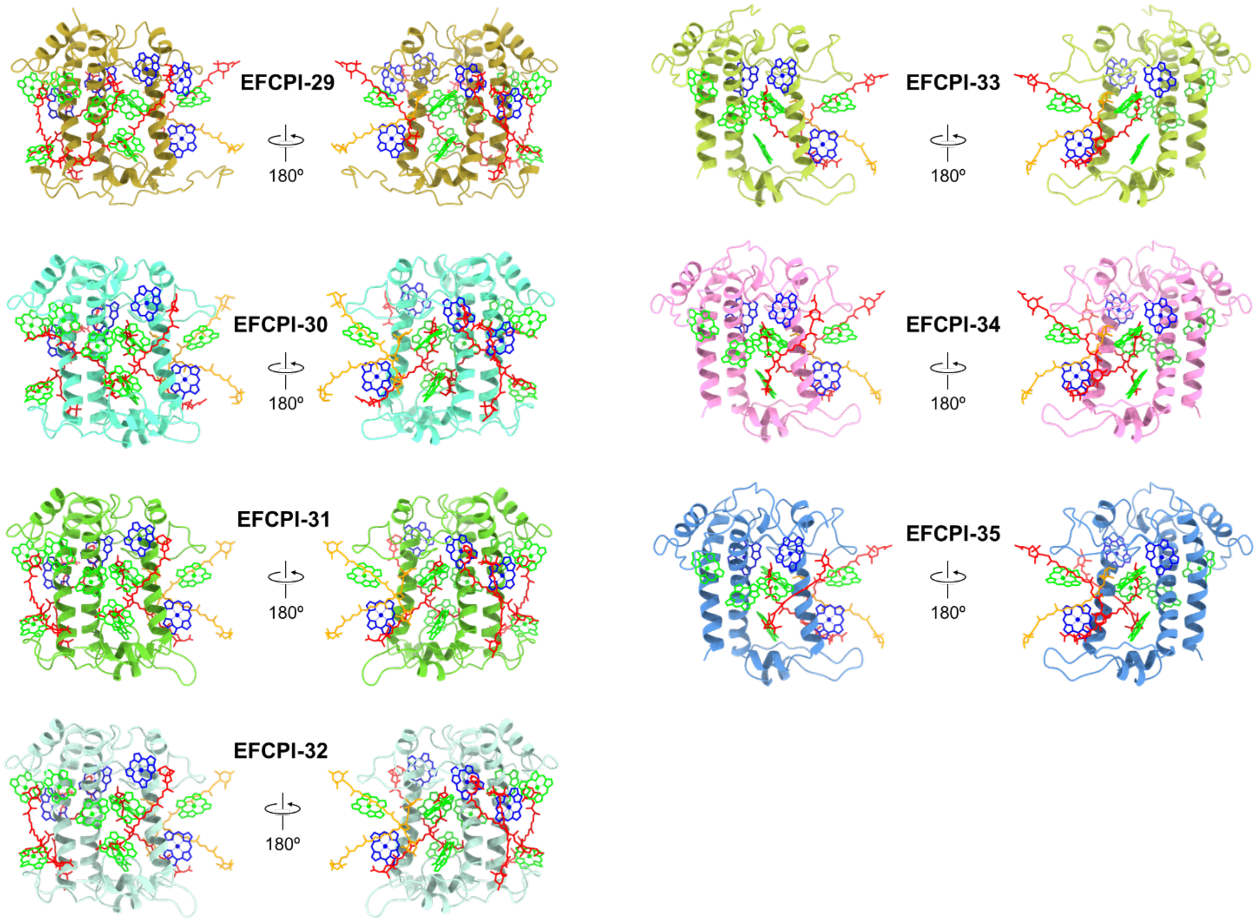

Fig. S5. Structures of individual 35 EFCPI subunits. Chl *a*, Chl *c*, diadinoxanthin, and fucoxanthin are colored in lime, blue, orange, and red, respectively. The phytol chains of Chls are omitted.

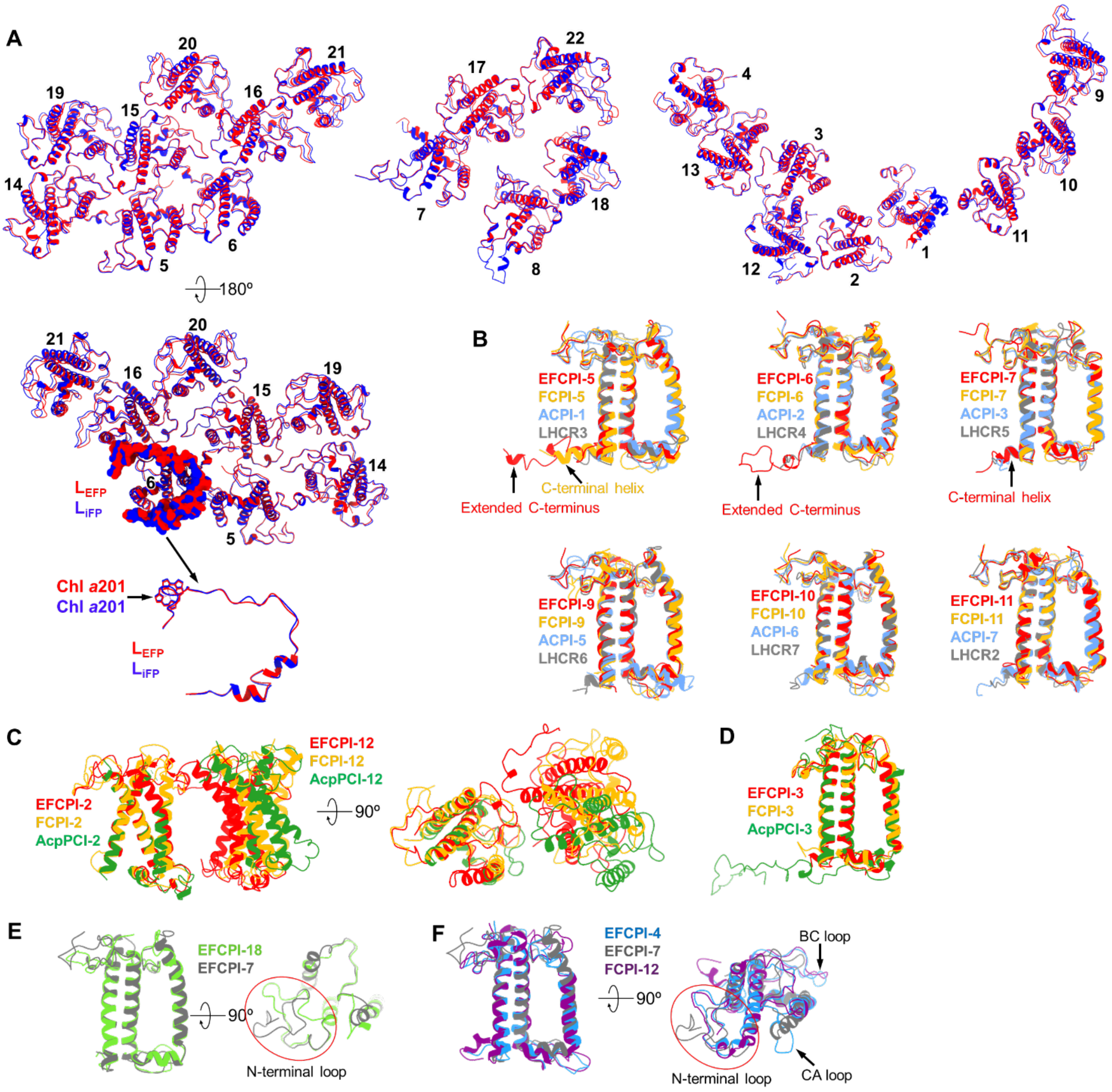

Fig. S6. Structural comparison of the EFCPIs with LHCIs from other red-lineage algae.

(**A**) Superposition of EFCPI-(1-22) with the corresponding iFCPIs in coccolith-lack haptophyte (PDB: 8Z11). The location and structure of L_{EFP} and comparison with L_{iFP} in coccolith-lack haptophyte are indicated.

(**B**) Comparison of Lhcr-type EFCPIs with the corresponding LHCRs from red algae (PDB: 7Y5E), ACPIs from cryptophyte (PDB: 7Y7B), and FCPIs from diatom (PDB: 6LY5). C-termini of EFCPI-5/6/7 and FCPI-5 are indicated by arrows.

(**C**) Superposition of EFCPI-2 with the corresponding FCPI-2 from diatom and AcpPCI-2 from dinoflagellate (PDB: 8JJR), exhibiting the arrangement of FCPI-12 and AcpPCI-12 relative to EFCPI-2.

(**D**) Structural comparison of EFCPI-3 and the corresponding FCPI-3 and AcpPCI-3.

(**E**) Structural comparison of EFCPI-18 with EFCPI-7.

(**F**) Structural comparison of EFCPI-4 with EFCPI-7 and FCPI-12. N-terminal loops are highlighted by red circles. The BC loop and CA loop are indicated by arrows.

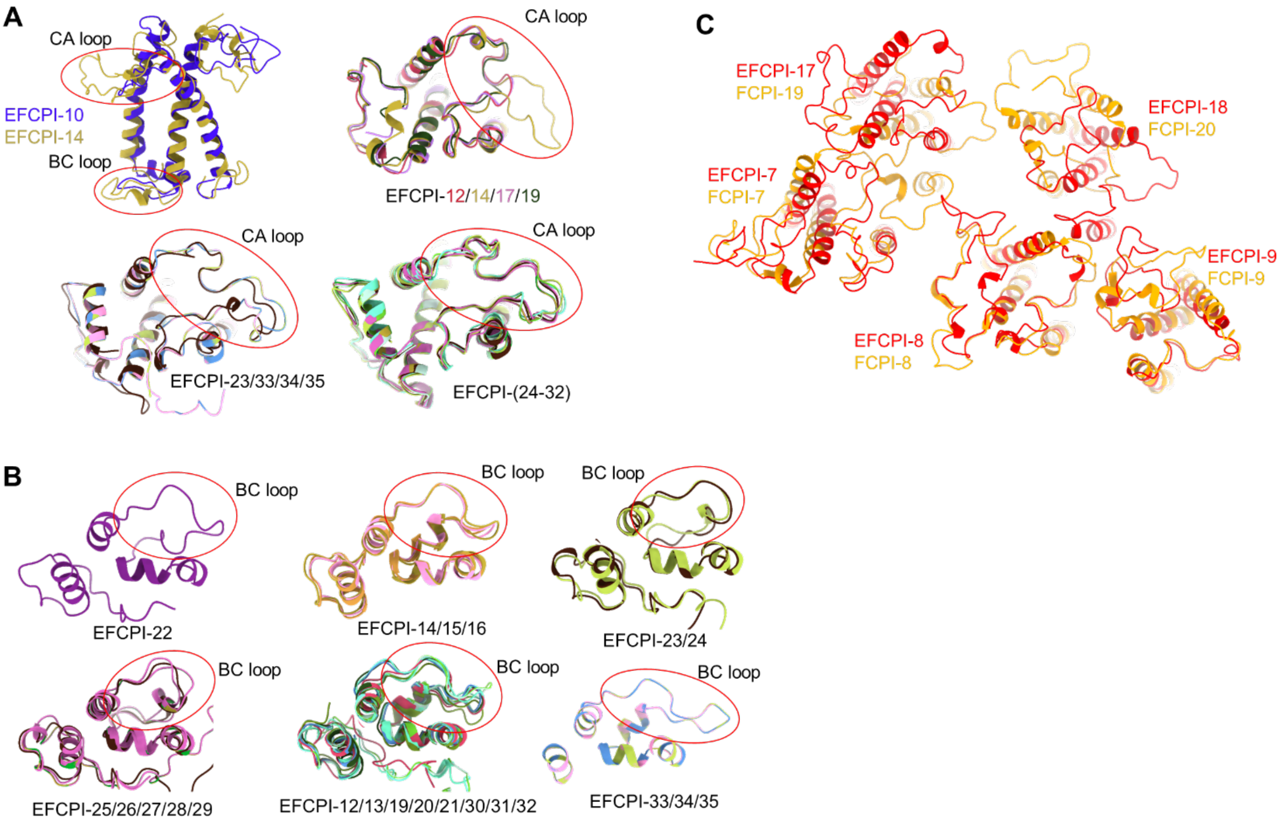

Fig. S7. Structural comparison of Lhcq-type EFCPIs.

(**A**) Superposition of Lhcq-type EFCPIs and comparisons with Lhcr-type EFCPI-10, showing the structural differences among their CA loops indicated by red circles.

(**B**) Superposition of Lhcq-type EFCPIs, showing the structural differences among their BC loops indicated by red circles.

(**C**) Superposition of EFCPI-8 with the corresponding FCPI-8 from diatom, exhibiting the arrangement of adjacent FCPIs relative to EFCPIs.

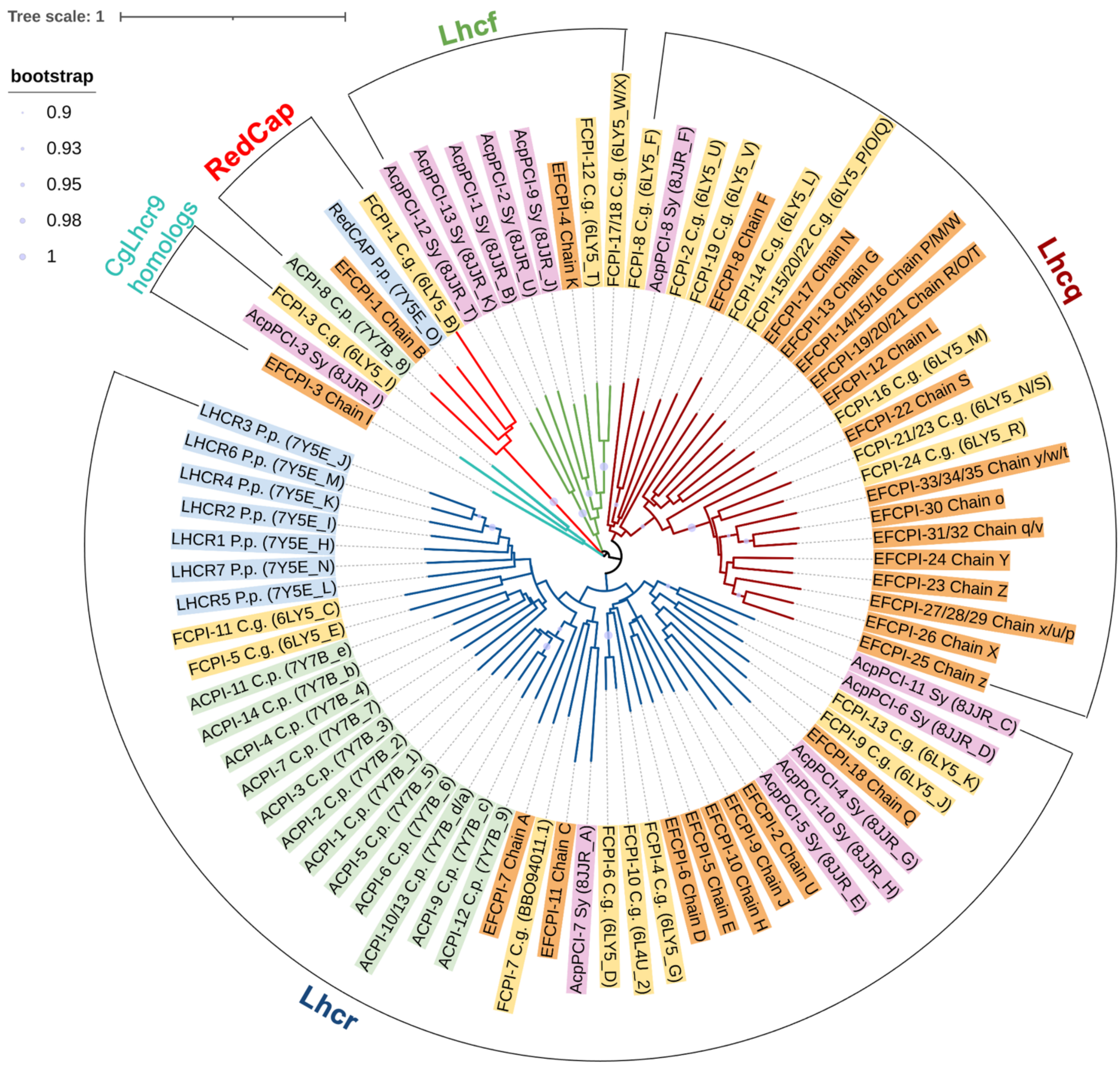

Fig. S8. Phylogenetic tree of LHCIs in haptophyte *E. huxleyi* (*E.h.*) (orange), red alga *P. purpureum* (*P.p.*) (blue), cryptophytic *C. placoidea* (*C.p.*) (green), diatom *C. gracilis* (*C.g.*) (yellow), and dinoflagellate *Symbiodiniums* (*Sy*) (pink). The Neighbor-Joining tree was based on amino acid sequences of LHCIs. The tree was built using 452 amino acid residues, and a bootstrap test (1000 replicates) was conducted. Chain ID of each EFCPI is labeled. The PDB ID and Chain ID of ACPIs, FCPIs and AcpPCIs are indicated in brackets in the form of “PDB ID_Chain ID”.

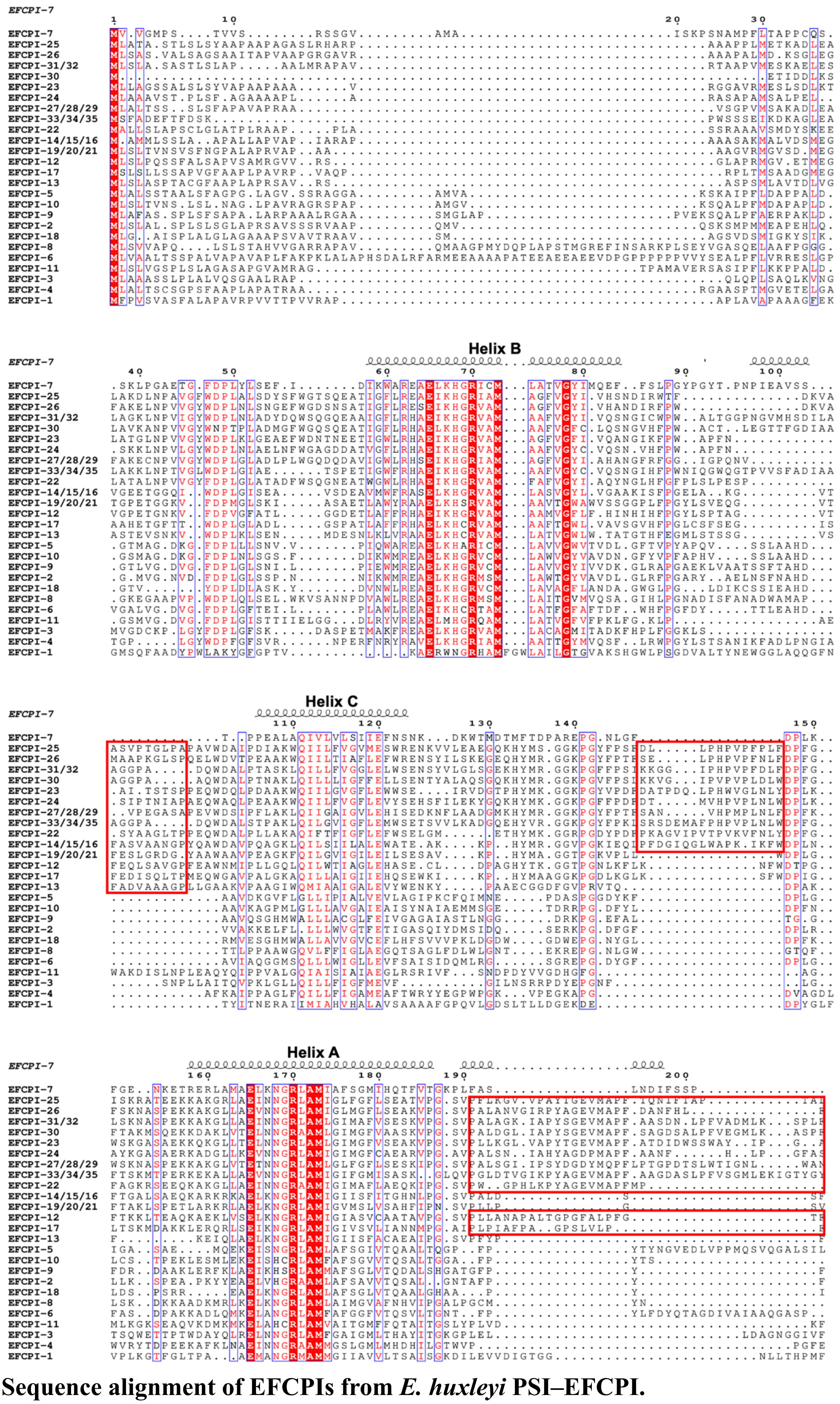

Fig. S9. Sequence alignment of EFCPIs from *E. huxleyi* PSI–EFCPI.

The secondary structure is shown above the sequences. Fully conserved residues are shaded in red, and similar amino acids are highlighted by blue frames. The extended domains of BC loop, CA loop and C-terminus are highlighted by red boxes.

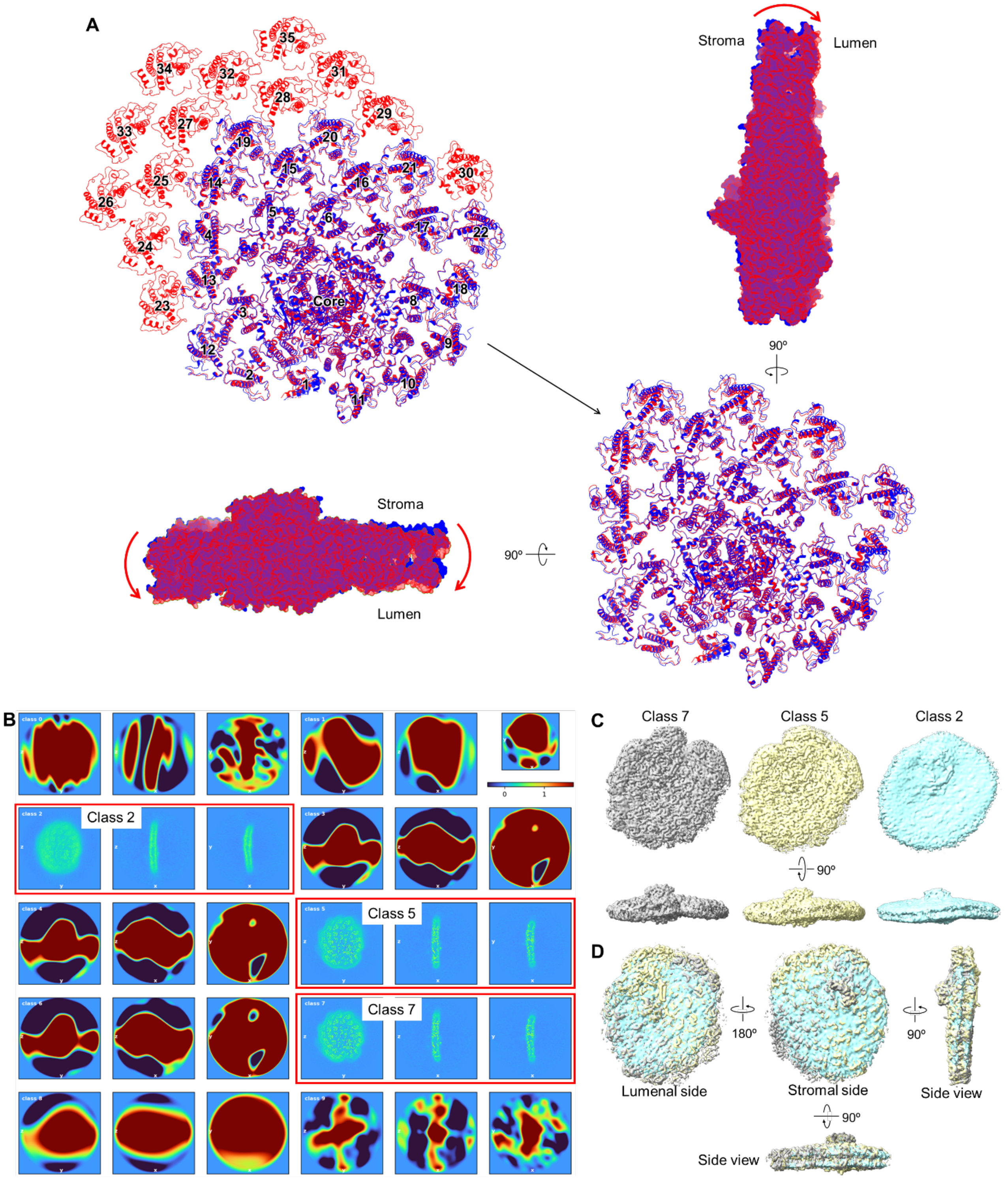

Fig. S10. Structural comparison of the coccolithophore PSI–EFCPI (red) with coccolith-lack haptophyte PSI–iFCPI (blue, PDB: 8Z11), and 3D classification of PSI–EFCPI supercomplex and comparation of the three meaningful classes.

(**A**) EFCPI-1 to 22 share similar arrangement pattern with those of iFCPI-1 to 22, whereas shift occurs on most of the EFCPIs compared with iFCPIs, and the arrangement of antennas in PSI–EFCPI bent towards the lumen.

(**B**) 3D classification (without alignment) of particles into 10 classes by default in CryoSPARC. Three meaningful classes containing the largest number of particles were highlighted.

(**C**) Conformations of the three meaningful classes.

(**D**) Superimposition of the three classes, showing nearly identical tilt of the antenna system.

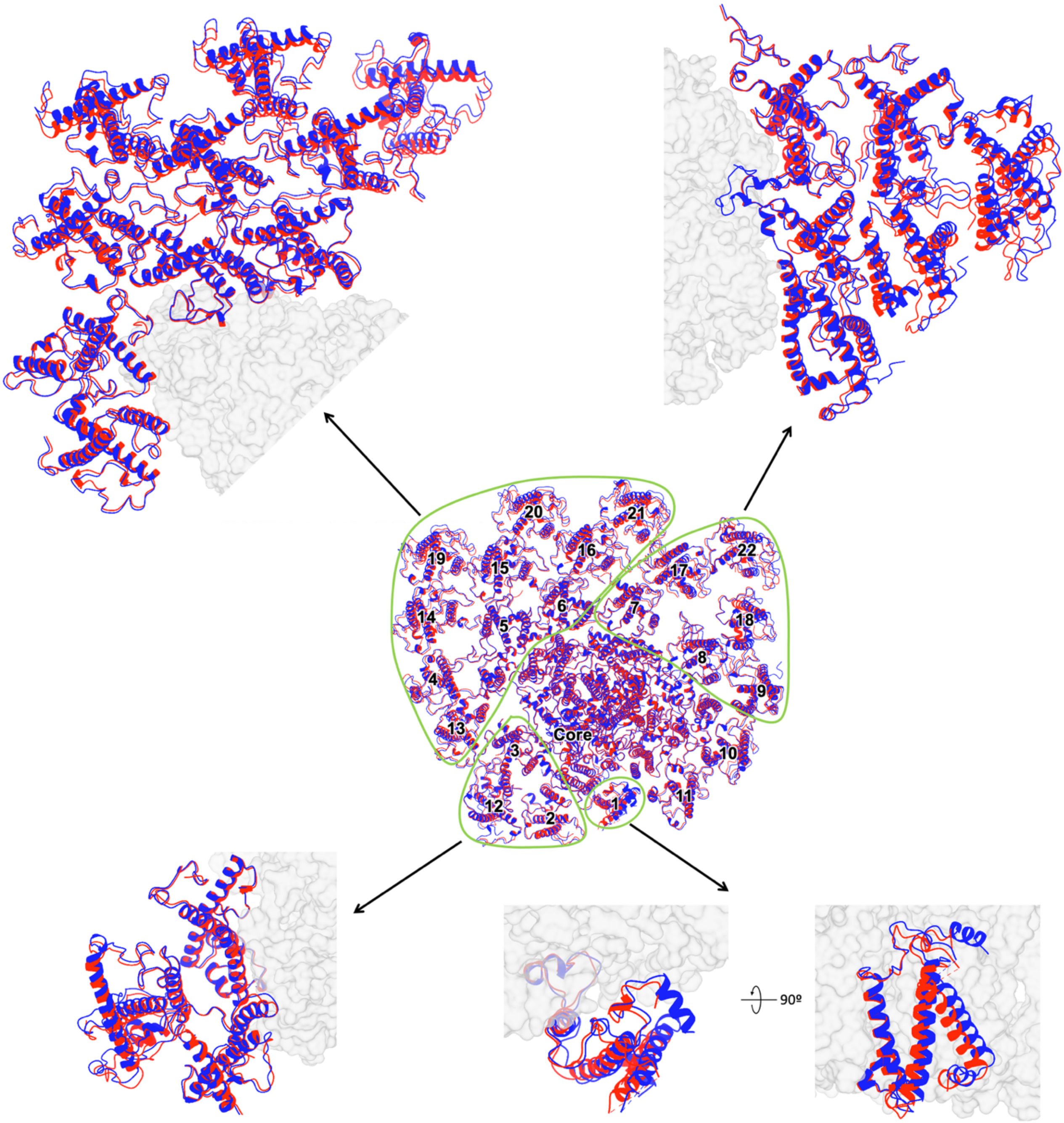

Fig. S11. Comparison of the arrangement of the coccolithophore EFCPI-(1-22) (red) with the corresponding iFCPIs from coccolith-lacking haptophyte (blue, PDB: 8Z11).

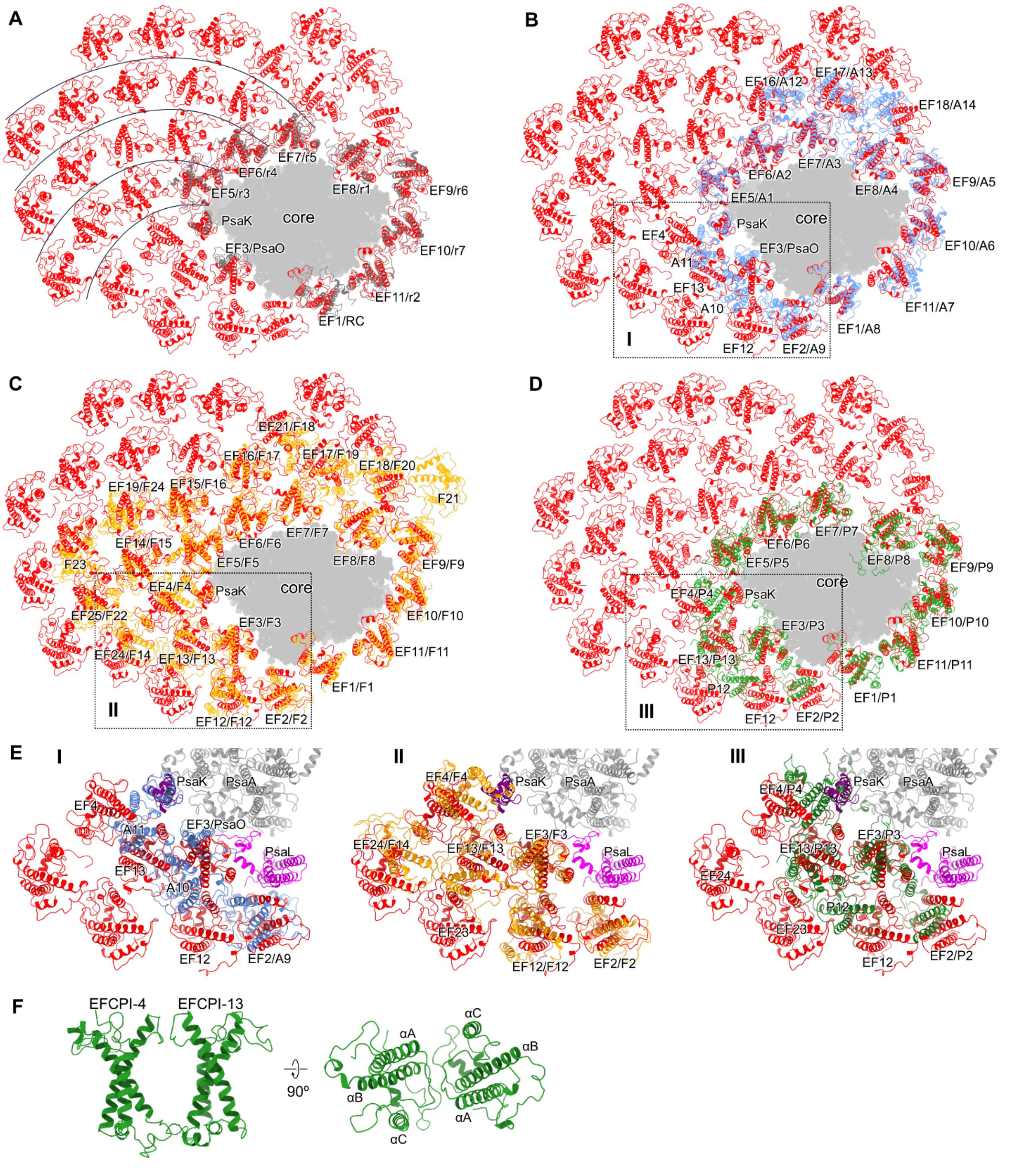

Fig. S12. Comparison of the arrangements of EFCPIs in coccolithophore PSI–EFCPI with those in red algae PSI–LHCR, cryptophyte PSI–ACPI, diatom PSI–PCPI, dinoflagellate *Symbiodinium* PSI–AcpPCI.
(**A**) Superposition of the PSI–EFCPI (red) with red algal PSI–LHCR (grey, PDB: 7Y5E). PsaK and PsaO of red algae are labeled. Letters of EF and r1-r7 indicate EFCPI and Lhcr1-7, respectively. RC indicates RedCAP.
(**B**) Superposition of the PSI–EFCPI (red) with cryptophyte PSI–ACPI (blue, PDB: 7Y7B). PsaK and PsaO of cryptophyte are labeled. Letters of EF and A1-A14 indicate EFCPI and ACPI1-14, respectively.
(**C**) Superposition of the PSI–EFCPI structure (red) with diatom PSI–FCPI structure (orange, PDB: 6LY5). Letters of EF and F1-F24 indicate EFCPI and FCPI1-24, respectively.
(**D**) Superposition of the PSI–EFCPI structure (red) with dinoflagellate PSI–AcpPCI structure (green, PDB: 8JJR). Letters of EF and P1-P13 indicate EFCPI and AcpPCI1-24, respectively.
(**E**) Enlarged view of the boxed areas in (**B**), (**C**), and (**D**) to show more details.
(**F**) Symmetric distribution of EFCPI-4 and EFCPI-13.

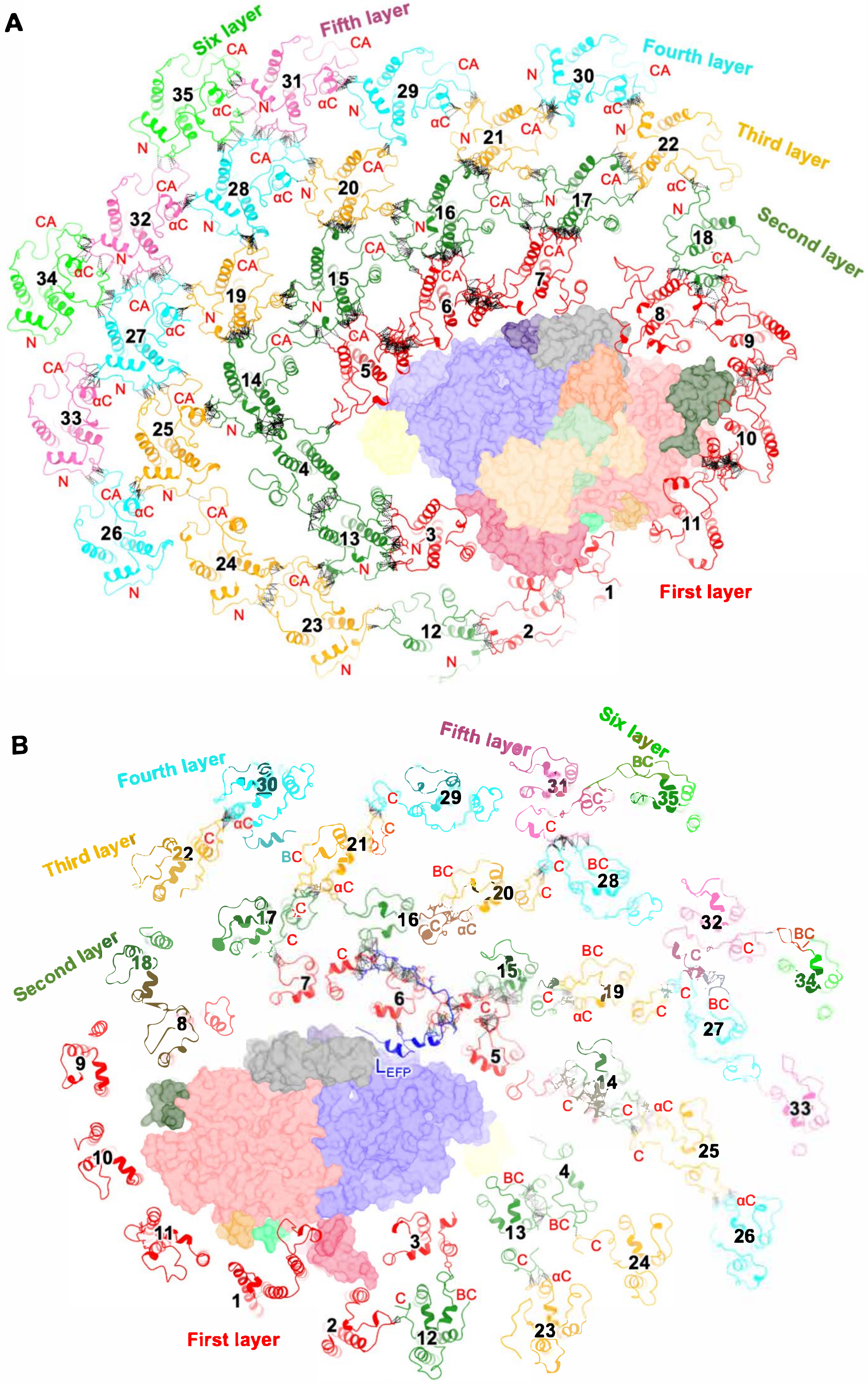
A
Six layer
Fifth layer
Fourth layer
Third layer
Second layer
First layer
B
Fourth layer
Fifth layer
Six layer
Third layer
Second layer
First layer
LEFP

c

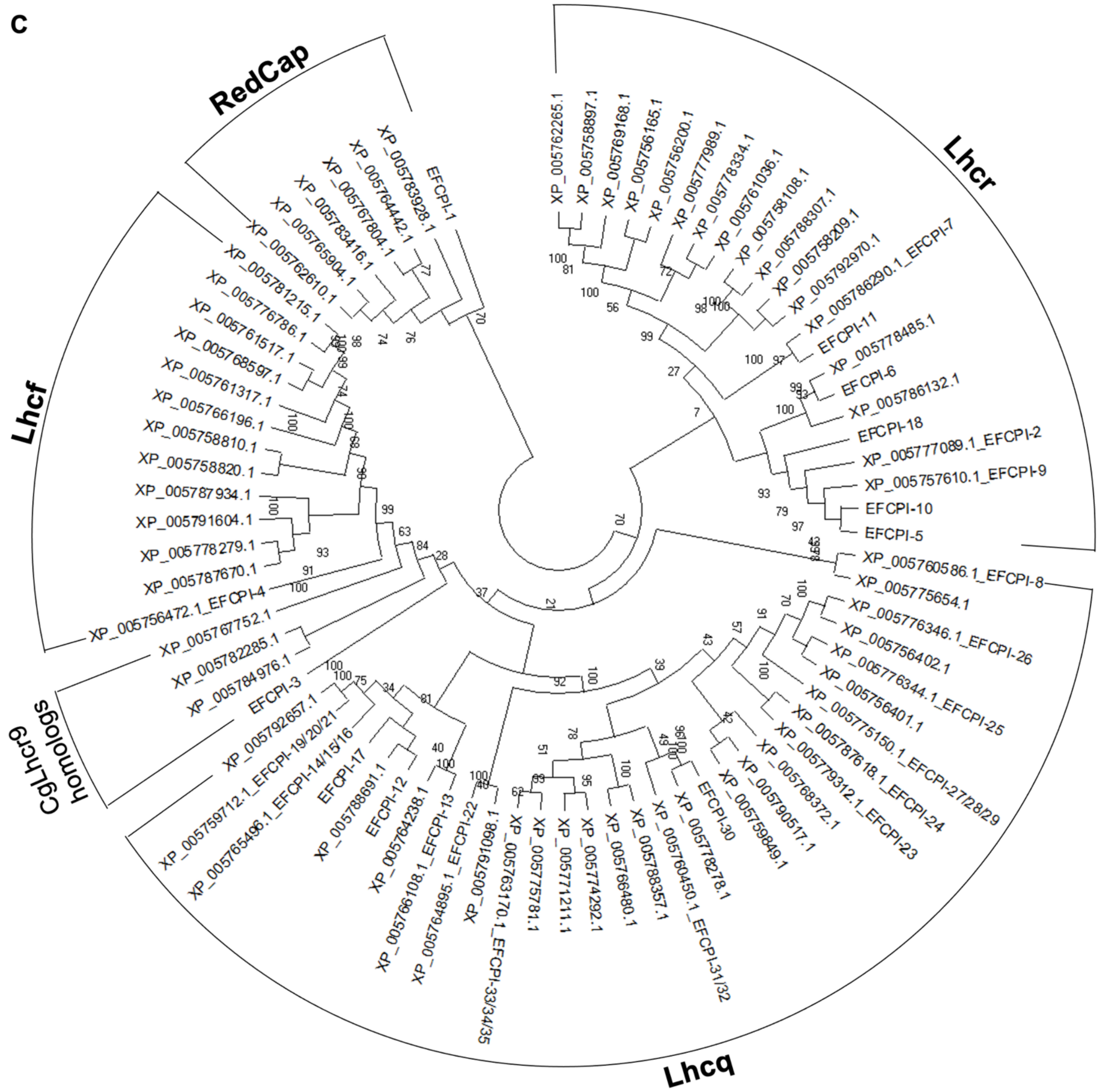

Fig. S13. Interactions between the EFCPIs in coccolithophore PSI–EFCPI and the phylogenetic tree of LHCs from haptophyte *E. huxleyi*.

(**A**) Interactions between EFCPIs at the stromal side mediated by N-terminal loop (N), CA loop (CA), and helix C (αC), indicating by black lines.

(**B**) Interactions between EFCPIs at the lumenal side mediated by C-terminal loop (C), BC loop (BC), and helix C (αC), indicating by black lines.

(**C**) Phylogenetic tree of EFCPIs and LHCs from *E. huxleyi*. Sequences of LHCs from *E. huxleyi* were obtained from National Center for Biotechnology Information. The Neighbor-Joining tree was based on amino acid sequences of EFCPIs and LHCs. The tree was built using 463 amino acid residues, and a bootstrap test (1000 replicates) was conducted.

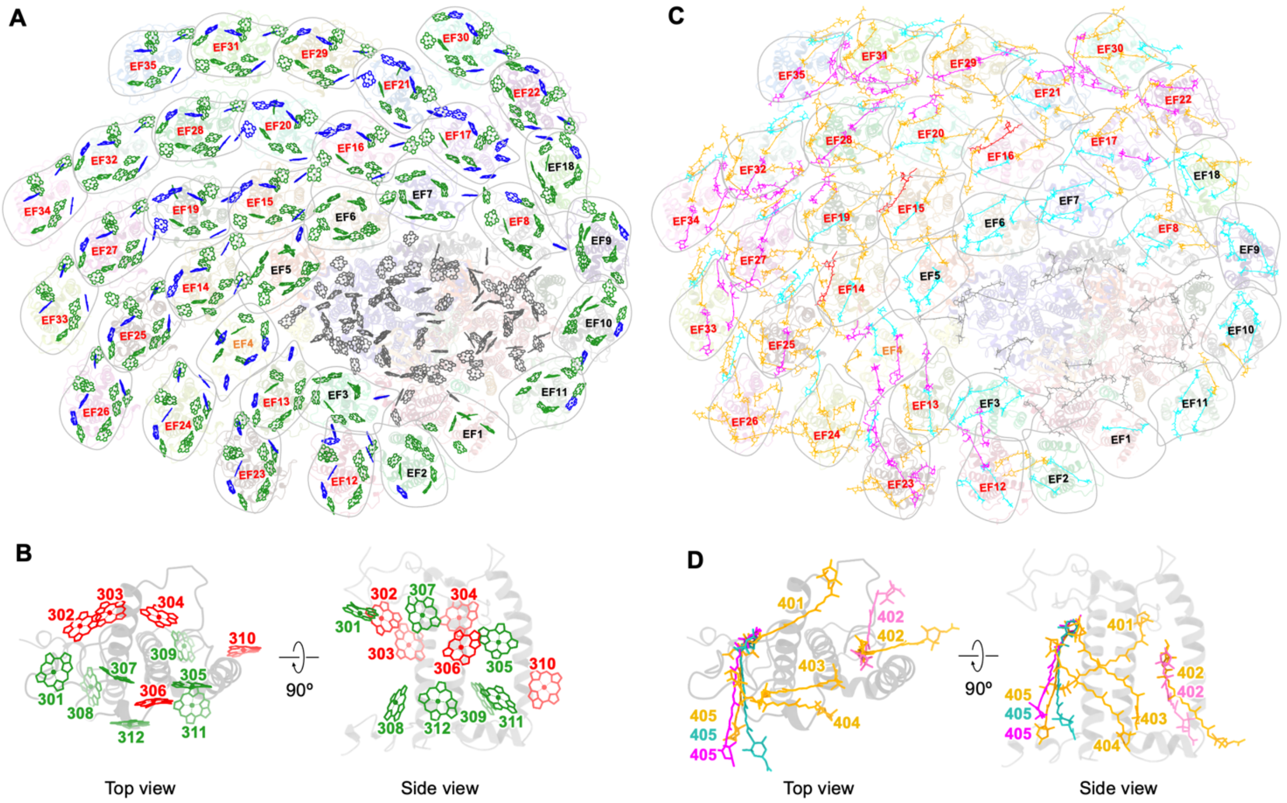

Fig. S14. Arrangement of the pigments in coccolithophore PSI–EFCPI.

(**A**) Arrangement of all Chls in PSI–EFCPI. The core Chls are colored in gray. Green and blue Chls are Chls *a* and Chls *c*, respectively. EF1-EF35 represent EFCPI-1 to iFCPI-35 (black: Lhcr-type EFCPIs; orange: Lhcf-type EFCPIs; red: Lhcq-type EFCPIs).

(**B**) Numbering of the 12 conserved Chl sites in EFCPIs with side view and top view, respectively. Green Chls indicate Chl *a*. Blue Chls indicate Chl *c*. Red Chls are mix sites for Chl *a* and Chl *c*.

(**C**) Arrangement of all Cars in the PSI–EFCPI. The core Cars are colored in gray. Orange, purple, cyan, and red Cars are Fx, hFx, Ddx, and GyrE, respectively. EF1-EF35 represent EFCPI-1 to iFCPI-35 (black: Lhcr-type EFCPIs; orange: Lhcf-type EFCPIs; red: Lhcq-type EFCPIs).

(**D**) Numbering of the 5 conserved Car sites in EFCPIs with side view and top view, respectively.

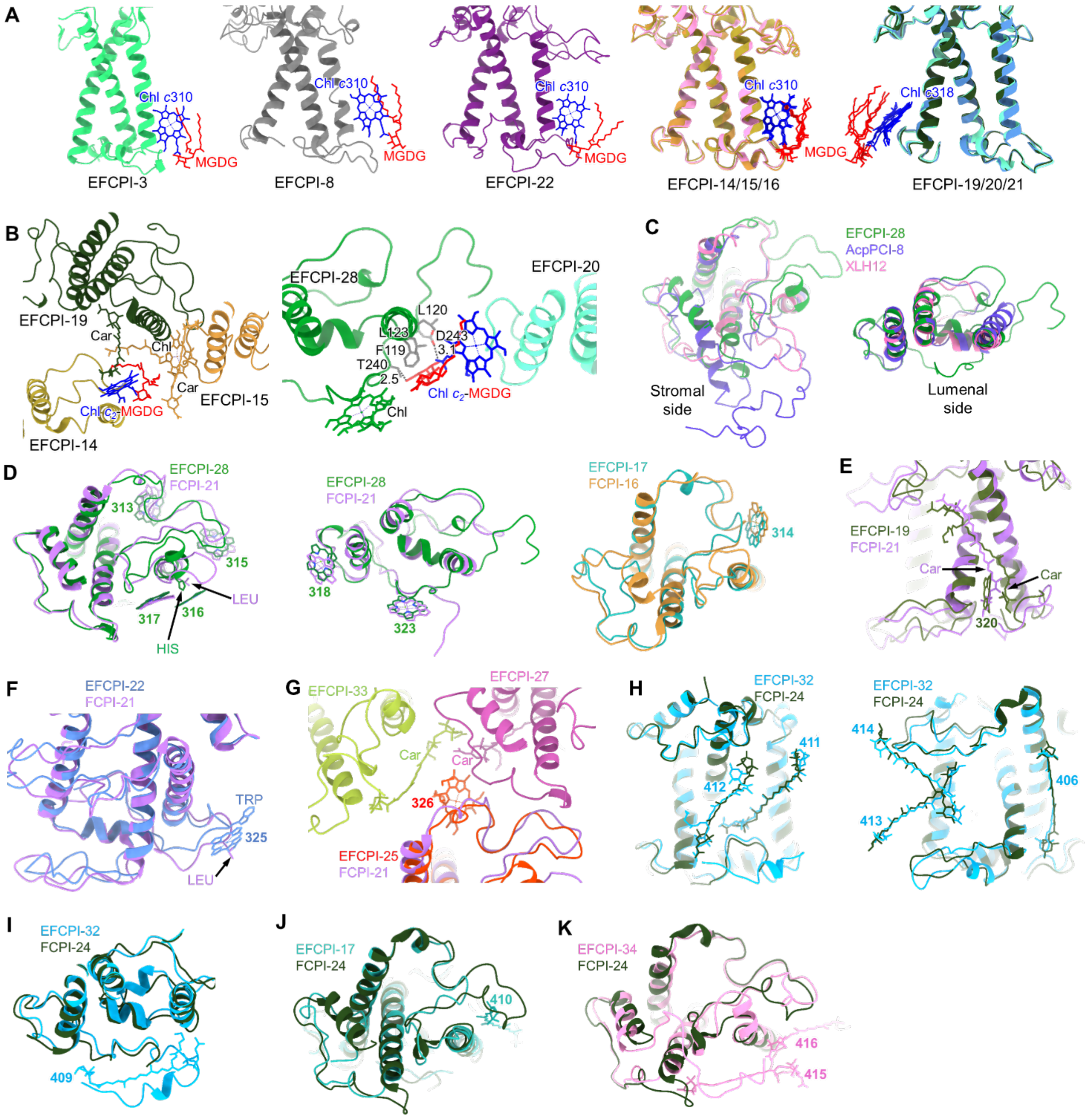

Fig. S15. Structures and locations of Chl c_2-MGDG molecules in EFCPIs and comparation the structure features for the non-conserved pigment-binding sites in Lhcq-type EFCPIs with those in Lhcq-type LHCIs from diatom, dinoflagellate, and xanthophyceae.

(**A**) The structures and locations of Chl c_2-MGDG molecules in EFCPIs.

(**B**) The interactions of Chl c_2-MGDG with adjacent EFCPIs.

(**C**) Structure comparation of the Lhcq-type EFCPI, dinoflagellate Lhcq-type AcpPCI (PDB: 8JJR) and xanthophyceae Lhcq-type XLH (PDB: 9M4F).

(**D-G**) Comparation of the structure features for the non-conserved Chl-binding sites in Lhcq-type EFCPIs and in diatom Lhcq-type FCPIs (PDB: 6LY5).

(**H-K**) Comparation of the structure features for the non-conserved Car-binding sites in Lhcq-type EFCPIs and in diatom Lhcq-type FCPIs (PDB: 6LY5).

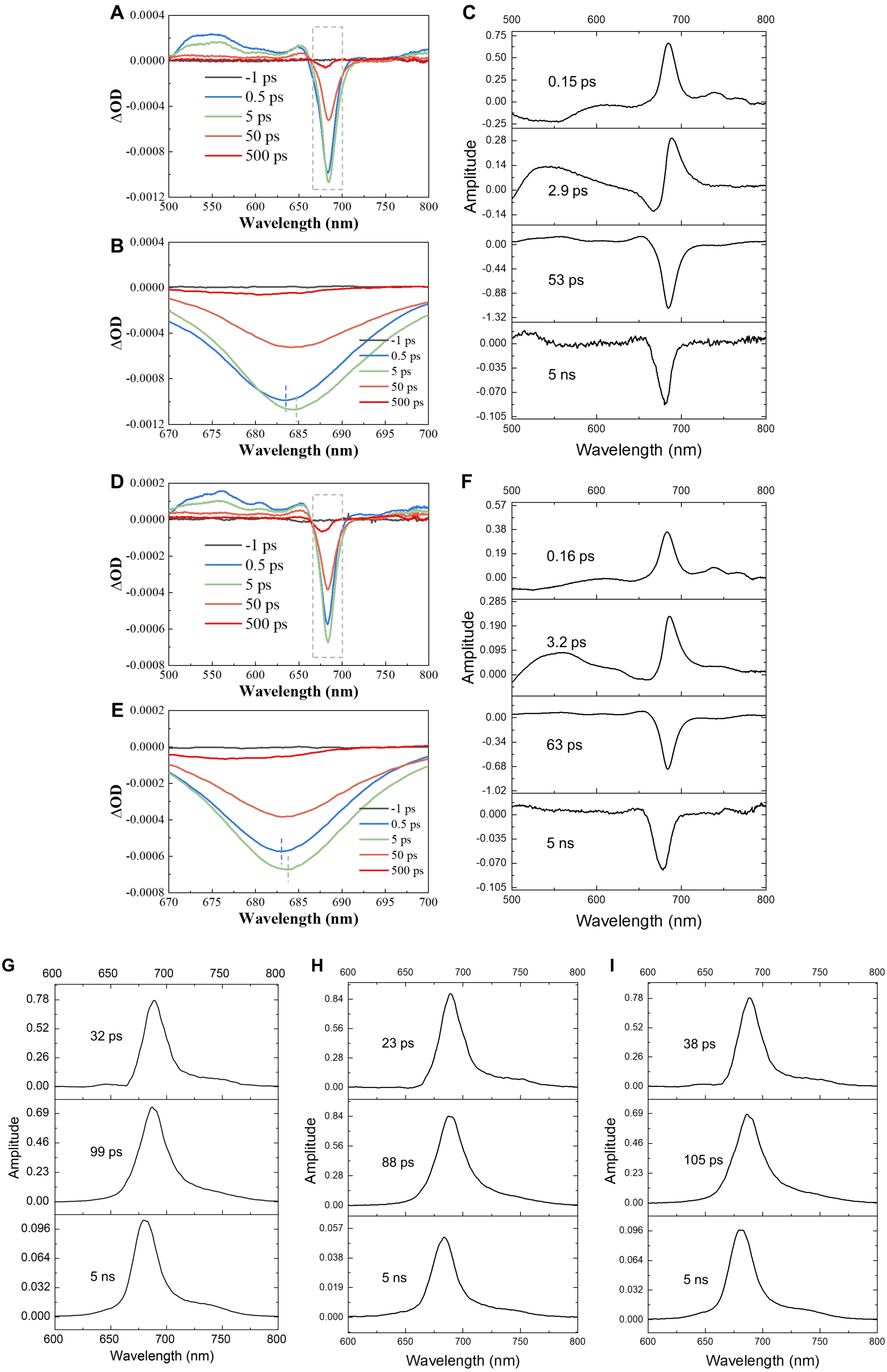
A
B
C
D
E
F
G
H
I
ΔOD
Wavelength (nm)
Amplitude
-1 ps
0.5 ps
5 ps
50 ps
500 ps
0.15 ps
2.9 ps
53 ps
5 ns
0.16 ps
3.2 ps
63 ps
32 ps
99 ps
23 ps
88 ps
38 ps
105 ps

Fig. S16. Ultrafast absorption and fluorescence spectroscopic analysis of PSI–LHCI from cryptophyte and haptophyte, compared with coccolithophore PSI–EFCPI (Fig. 4).

(**A**) Femtosecond time-resolved transient absorption spectra of PSI–ACPI from cryptophyte *Chroomonas placoidea*. The excitation wavelength is set at 445. Five time-resolved spectra of -1 ps, 0.5 ps, 5 ps, 50 ps, and 500 ps are shown. The boxed area is shown in (**B**).

(**B**) Zoom-in view of the boxed area in (**A**). The peak of 0.5 ps and 5 ps spectra are indicated.

(**C**) Transient absorption decay-associated spectra of cryptophyte PSI–ACPI.

(**D**) Femtosecond time-resolved transient absorption spectra of PSI–ACPI from coccolith-lacking haptophyte *Isochrysis galbana*. The excitation wavelength is set at 445. Five time-resolved spectra of -1 ps, 0.5 ps, 5 ps, 50 ps, and 500 ps are shown. The boxed area is shown in (**E**).

(**E**) Zoom-in view of the boxed area in (**D**). The peak of 0.5 ps and 5 ps spectra are indicated.

(**F**) Transient absorption decay-associated spectra of coccolith-lacking haptophyte PSI–iFCPI.

(**G**) Fluorescence decay-associated spectra of coccolithophore PSI–EFCPI.

(**H**) Fluorescence decay-associated spectra of cryptophyte PSI–EFCPI.

(**I**) Fluorescence decay-associated spectra of coccolith-lacking haptophyte PSI–EFCPI.

The excitation wavelength for time-resolved fluorescence spectra is set at 445. Fluorescence decay-associated spectra of coccolithophore PSI–EFCPI exhibits three components. The first component displays peaks at 642 nm and 688 nm, which may originate from Chl *c* and Chl *a* in EFCPIs, respectively, and lower positive signals above 700 nm, suggesting EET from EFCPIs to the PSI core. As energy quenching of LHCs occurs at hundreds of picoseconds, the positive peak in the second component may be mainly attributed to energy quenching through interactions among the pigments of EFCPIs. The first components of PSI–EFCPI and coccolith-lacking haptophyte PSI–LHCI exhibited increased time constants compared to that of the cryptophyte PSI–LHCI, indicating that energy transfer from antennas to PSI core will take longer in haptophyte PSI–LHCI. This is likely due to the large number of LHCIs in PSI–EFCPI.

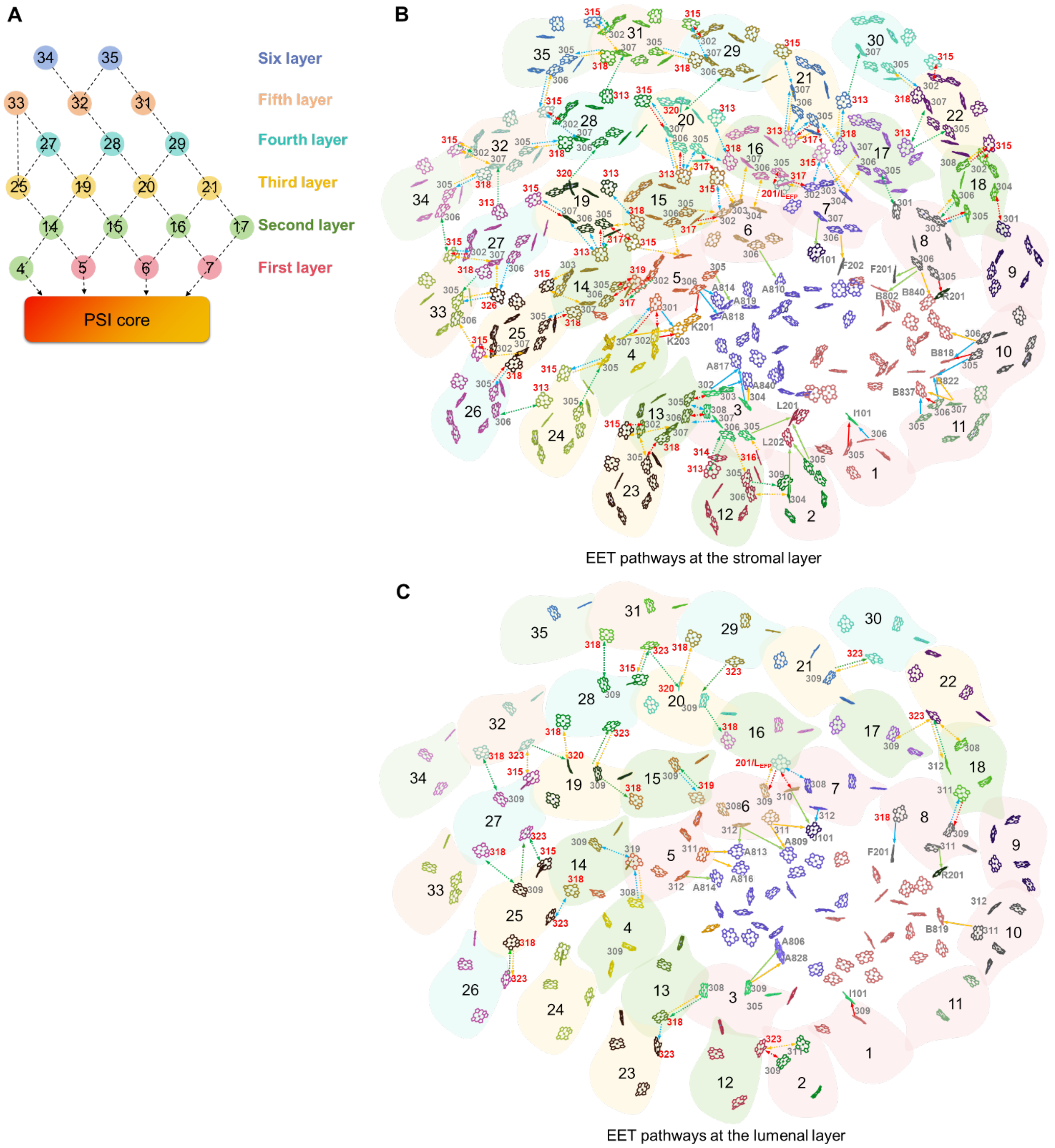

Fig. S17. The detailed EET pathways key Chls mediating the EET pathways in PSI–EFCPI based on Förster theory.

(**A**) The EET routes (dashed lines) used for calculating the average time constant of the EET routes from the EFCPIs in fifth and sixth layers to the PSI core.

(**B**) (**C**) Detailed EET pathways at the stromal layer (**B**) and lumenal layer (**C**) from the EFCPIs to PSI core (solid arrows) and between the EFCPIs (dashed arrows) viewed from the stromal side. Red arrows: faster than 2 ps; blue arrows: between 2 and 5 ps; orange arrows: between 5 and 10 ps; green arrows: between 10 and 20 ps. The conserved Chls and specific Chls are colored grey and red, respectively.

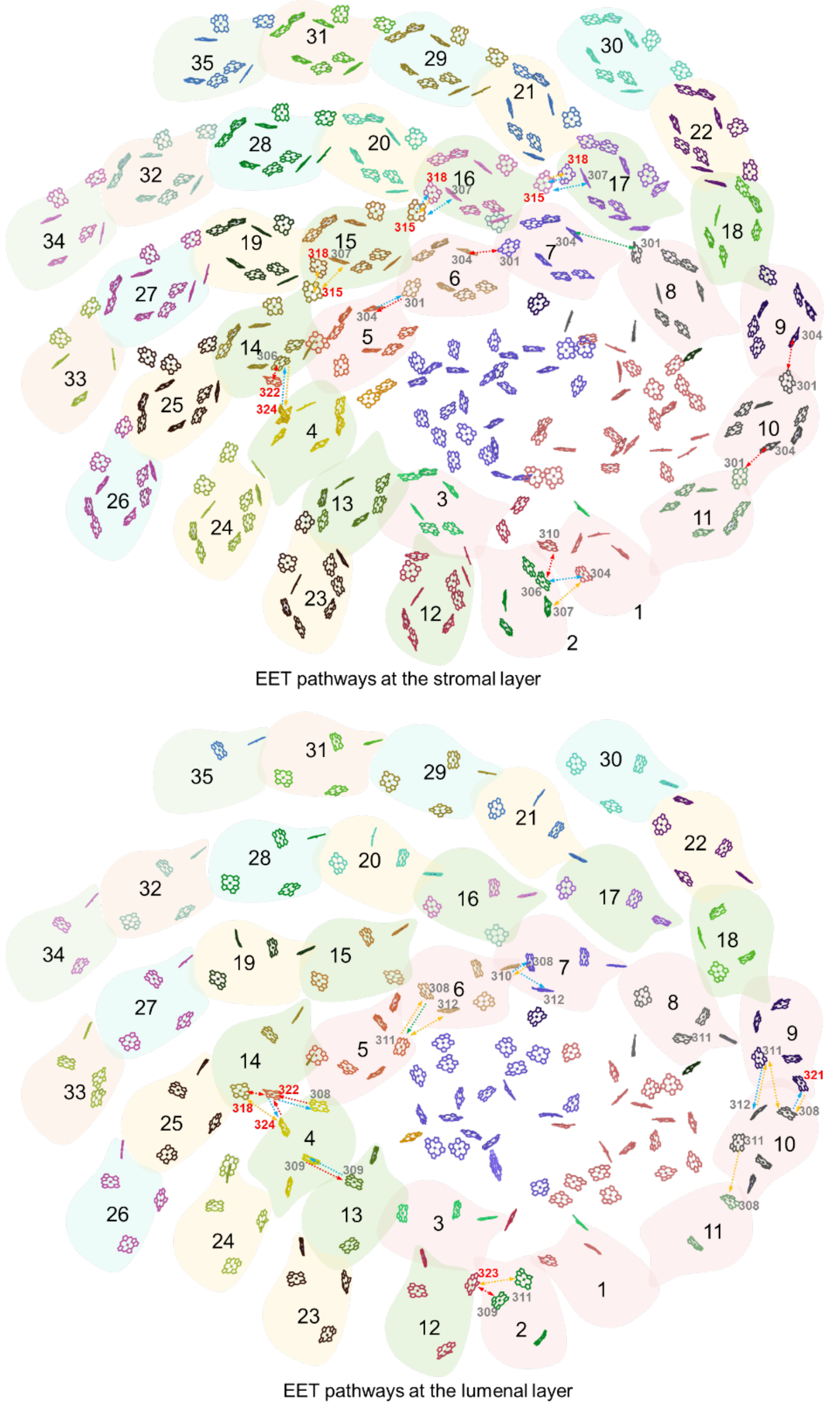

Fig. S18. The key Chls mediating the EET pathways between adjacent EFCPIs within the first and second layers based on Förster theory. Detailed EET pathways at the stromal layer and lumenal layer between the EFCPIs (dashed arrows) viewed from the stromal side. Red arrows: faster than 2 ps; blue arrows: between 2 and 5 ps; orange arrows: between 5 and 10 ps; green arrows: between 10 and 20 ps. The conserved Chls and specific Chls are colored grey and red, respectively. In the first layer, stromal EET between adjacent EFCPIs is mainly mediated by Chl 301$_{\text{EFCPI-(n)}}$ and Chl 304$_{\text{EFCPI-(n+1)}}$, similar to other red-lineage PSI–LHCIs. Because of the specific orientation of EFCPI-1 and EFCPI-2, EET between them is mediated by Chl 304/310$_{\text{EFCPI-1}}$ and Chl 306/307$_{\text{EFCPI-2}}$. On the lumenal side, Chl 308/310/311/312 mediate EET between adjacent EFCPIs. In the second layer, the stromal EET among EFCPI-14/15/16/17 is mediated by Chl 315$_{\text{EFCPI-(n)}}$ and Chl 307/318$_{\text{EFCPI-(n+1)}}$. EET between EFCPI-4 and EFCPI-13 is mediated by Chl 309 on the lumenal side.

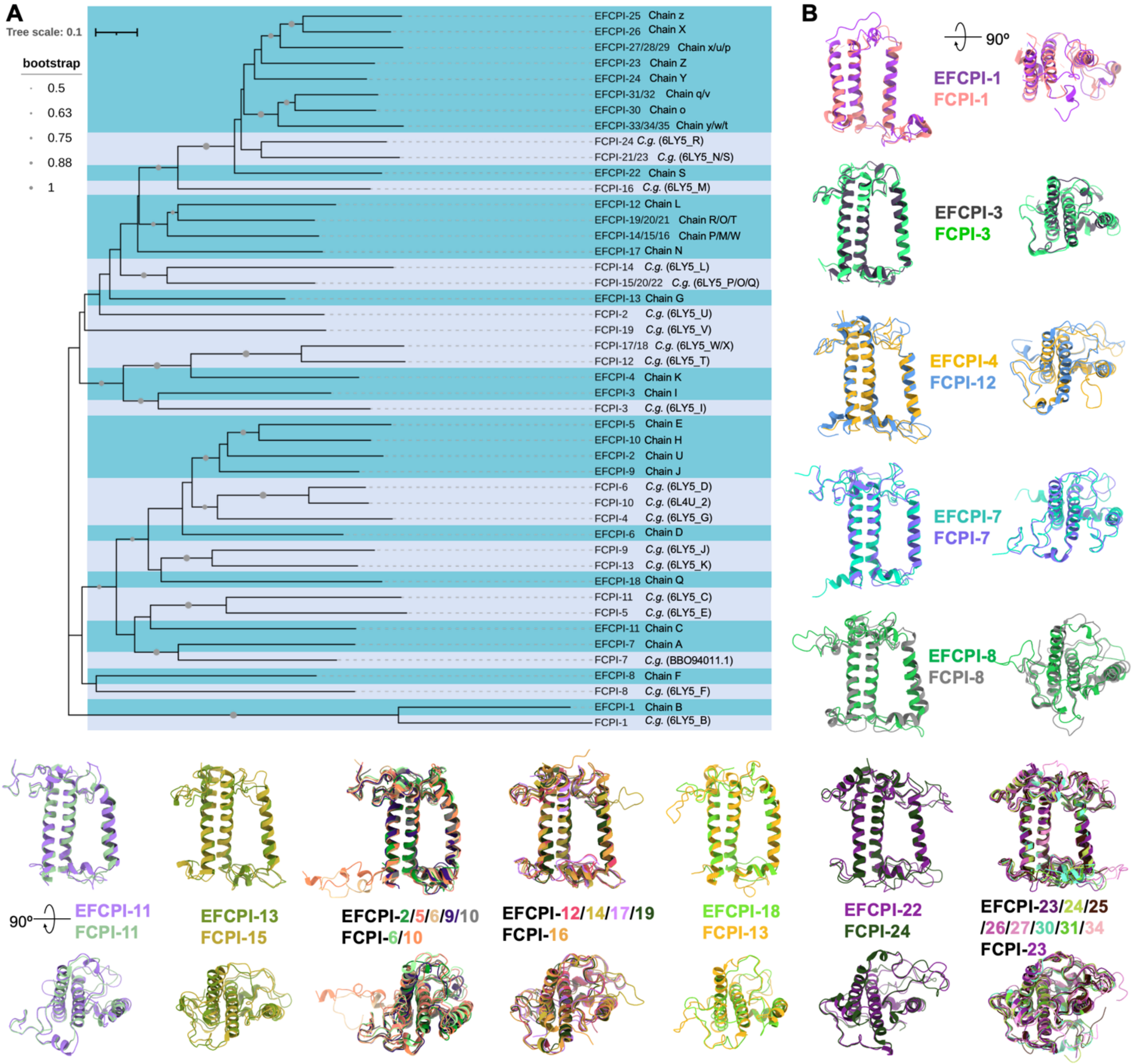

Fig. S19. Phylogenetic analysis and structural comparison of EFCPIs in coccolithophore *Emiliania huxleyi* (*E.h.*) (sea blue) and FCPIs in diatom *C. gracilis* (*C.g.*) (blue).

(**A**) The neighbor-joining tree was based on amino acid sequences of LHCIs. The tree was built with the Poisson model using 380 amino acid residues, and a bootstrap test (1,000 replicates) was conducted. Chain ID of each EFCPI is indicated. The PDB ID and Chain ID of FCPIs are indicated in brackets in the form of "PDB ID_Chain ID".

(**B**) Comparisons of EFCPIs and FCPIs in same clade.

Table S1. Cryo-EM data collection, refinement, and validation statistics.

	PSI–EFCPI (EMDB-64087; PDB-9UEN)
**Data Collection and Processing**	
Voltage (kV)	300
Electron exposure (e- /Å2)	50
Defocus range (um)	-1.2~-2.2
Pixel size (Å)	0.53
Symmetry imposed	C1
Initial particle images (no.)	621,828
Final particle images (no.)	93,213
Map resolution (Å)	3.10
FSC threshold	0.143
**Refinement**	
Initial model used (PDB code)	8Z11
Model resolution (Å)	3.10
FSC threshold	0.143
Map sharpening *B* factor ($Å^2$)	-61.2
Model composition	
Non-hydrogen atoms	110087
Protein residues	9159
Ligands	790
B factors ($Å^2$)	
Protein	60.05
Ligand	60.28
R.m.s. deviations	
Bond lengths (Å)	0.010
Bond angles (º)	1.368
Validation	
MolProbity score	1.61
Clashscore	5.95
Poor rotamers (%)	0.21
Ramachandran plot	
Favored (%)	95.89
Allowed (%)	3.91
Disallowed (%)	0.20

Table S2. Cofactors in each subunit of the coccolithophore PSI–EFCPI supercomplex.

Subunit	Traced residues	Chls	Cars	Lipids	Others
PsaA	741 (12-752)	44 a	4 β-Car	2 PG, 1 MGDG, 1 SQDG	1 PQN, 1 SF4
PsaB	731 (3-733)	42 a	7 β-Car	1 DGDG	1 PQN
PsaC	80 (2-81)				2 SF4
PsaD	140 (2-141)				
PsaE	63 (37-99)				
PsaF	161 (24-184)	2 a	1 β-Car		
PsaI	31 (3-33)	1 a	1 β-Car		
PsaJ	40 (1-40)	1 a	1 β-Car, 1 Ddx		
PsaK	70 (42-111)	3 a	1 β-Car, 1 Ddx	1 SQDG	
PsaL	143 (2-144)	3 a	3 β-Car		
PsaM	30 (1-30)		1 β-Car		
PsaR	91 (43-133)	1 a	1 β-Car, 1 hFx		
EFCPI-1	148 (47-159, 172-206)	6 a	1 Ddx		
EFCPI-2	144 (45-188)	7 a, 1 c	2 Ddx, 1 Fx		
EFCPI-3	155 (34-188)	8 a	4 Ddx		1 Chl c_2-MGDG
EFCPI-4	171 (32-202)	6 a, 4 c	2 Ddx, 1 Fx, 3 hFx		
EFCPI-5	187 (31-217)	14 a, 1 c	4 Ddx, 2 Fx	1 MGDG	
EFCPI-6	188 (68-255)	13 a	5 Ddx	4 MGDG	
EFCPI-7	183 (19-201)	10 a, 1 c	5 Ddx	1 MGDG	
EFCPI-8	188 (51-238)	9 a, 1 c	3 Ddx, 3 Fx	1 PG, 1 SQDG	1 Chl c_2-MGDG
EFCPI-9	165 (36-200)	11 a, 1 c	5 Ddx		
EFCPI-10	167 (30-196)	10 a, 2 c	4 Ddx, 1 Fx		
EFCPI-11	178 (31-208)	8 a, 1 c	3 Ddx, 1 Fx		
EFCPI-12	189 (40-228)	8 a, 6 c	2 Ddx, 1 Fx, 2 hFx	1 MGDG	
EFCPI-13	168 (30-197)	7 a, 2 c	1 Ddx, 2 Fx, 1 hFx		
EFCPI-14	193 (32-224)	7 a, 4 c	1 Ddx, 4 Fx, 1 GyrE	1 SQDG	1 Chl c_2-MGDG
EFCPI-15	193 (32-224)	7 a, 4 c	1 Ddx, 4 Fx, 1 GyrE	1 SQDG	1 Chl c_2-MGDG
EFCPI-16	193 (32-224)	7 a, 4 c	1 Ddx, 4 Fx, 1 GyrE	1 SQDG	1 Chl c_2-MGDG
EFCPI-17	194 (32-225)	6 a, 7 c	3 Ddx, 3 Fx, 1 hFx	1 MGDG	
EFCPI-18	156 (31-186)	9 a, 1 c	4 Ddx, 2 Fx		
EFCPI-19	179 (32-210)	6 a, 5 c	1 Ddx, 4 Fx, 1 hFx		1 Chl c_2-MGDG
EFCPI-20	179 (32-210)	6 a, 5 c	1 Ddx, 4 Fx, 1 hFx		1 Chl c_2-MGDG
EFCPI-21	179 (32-210)	6 a, 5 c	1 Ddx, 4 Fx, 1 hFx	1 MGDG	1 Chl c_2-MGDG
EFCPI-22	210 (34-243)	10 a, 4 c	5 Fx, 2 hFx	1 PG	1 Chl c_2-MGDG
EFCPI-23	219 (32-250)	9 a, 4 c	1 Ddx, 4 Fx, 4 hFx		
EFCPI-24	213 (30-242)	9 a, 5 c	1 Ddx, 7 Fx		
EFCPI-25	222 (36-257)	10 a, 4 c	2 Ddx, 6 Fx, 1 hFx		
EFCPI-26	211 (35-245)	9 a, 4 c	1 Ddx, 8 Fx		
EFCPI-27	228 (27-254)	9 a, 5 c	1 Ddx, 6 Fx, 2 hFx		
EFCPI-28	228 (27-254)	9 a, 5 c	1 Ddx, 6 Fx, 2 hFx		
EFCPI-29	228 (27-254)	9 a, 5 c	1 Ddx, 6 Fx, 2 hFx		
EFCPI-30	236 (37-272)	9 a, 4 c	2 Ddx, 5 Fx, 3 hFx		
EFCPI-31	238 (31-268)	9 a, 4 c	2 Ddx, 5 Fx, 3 hFx		
EFCPI-32	238 (31-268)	9 a, 4 c	2 Ddx, 5 Fx, 3 hFx		
EFCPI-33	201 (13-213)	6 a, 3 c	1 Ddx, 2 Fx, 3 hFx		
EFCPI-34	211 (3-213)	6 a, 3 c	1 Ddx, 3 Fx, 3 hFx		
EFCPI-35	211 (3-213)	6 a, 3 c	1 Ddx, 3 Fx, 3 hFx		
L_{EFP}	47 (53-99)	1 a			
PSI–EFCPI	6274	387 a, 112 c	20 *β*-Car, 73 Ddx, 109 Fx, 42 hFx, 3 GyrE	4 PG, ,6 SQDG 10 MGDG, 1 DGDG	2 PQN, 3 SF4, 9 Chl c_2-MGDG

chlorophyll *a* (Chl *a*), chlorophyll *c* (Chl *c*), *β*-carotene (*β*-Car), fucoxanthin (Fx), 19′-hexanoyloxyfucoxanthin (hFx), Gyroxanthin-diester (GyrE), diadinoxanthin (Ddx), PG, phosphatidylglycerol; MGDG, monogalactosyldiacylglycerol; SQDG, sulfoquinovosyldiacylglycerol; DGDG, digalactosyldiacylglycerol; PQN, phylloquinone; SF4, sulphur–iron cluster.

Table S3. Binding sites of pigments in the 35 EFCPI subunits. Chl *c* is colored in red in contrast to Chl *a* in black.

Site	EF1	EF2	EF3	EF4	EF5	EF6	EF7	EF8	EF9	EF 10	EF 11	EF 12	EF 13	EF 14	EF 15	EF 16	EF 17
301					Chl *a*	Chl *a*	Chl *a*	Chl *a*	Chl *a*	Chl *a*	Chl *a*						
302			Chl *a*	Chl *a*	Chl *a*	Chl *a*	Chl *a*	Chl *c*	Chl *a*	Chl *a*	Chl *a*	Chl *a*	Chl *a*	Chl *c*	Chl *c*	Chl *c*	Chl *c*
303		Chl *a*	Chl *a*	Chl *c*	Chl *a*	Chl *a*	Chl *a*	Chl *a*	Chl *c*	Chl *c*	Chl *c*	Chl *c*	Chl *c*	Chl *c*	Chl *c*	Chl *c*	Chl *c*
304	Chl *a*	Chl *a*	Chl *a*	Chl *c*	Chl *a*	Chl *a*	Chl *a*	Chl *a*	Chl *a*	Chl *a*	Chl *a*	Chl *c*	Chl *a*	Chl *c*	Chl *c*	Chl *c*	Chl *c*
305	Chl *a*	Chl *a*	Chl *a*	Chl *a*	Chl *a*	Chl *a*	Chl *a*	Chl *a*	Chl *a*	Chl *a*	Chl *a*	Chl *a*	Chl *a*	Chl *a*	Chl *a*	Chl *a*	Chl *a*
306	Chl *a*	Chl *c*	Chl *a*	Chl *c*	Chl *a*	Chl *a*	Chl *a*	Chl *a*	Chl *a*	Chl *a*	Chl *a*	Chl *a*	Chl *a*	Chl *a*	Chl *a*	Chl *a*	Chl *a*
307	Chl *a*	Chl *a*	Chl *a*	Chl *a*	Chl *a*	Chl *a*	Chl *a*	Chl *a*	Chl *a*	Chl *a*	Chl *a*	Chl *a*	Chl *a*	Chl *a*	Chl *a*	Chl *a*	Chl *a*
308		Chl *a*	Chl *a*	Chl *a*	Chl *a*	Chl *a*	Chl *a*		Chl *a*	Chl *a*	Chl *a*						
309	Chl *a*	Chl *a*	Chl *a*	Chl *a*	Chl *a*	Chl *a*	Chl *a*	Chl *a*	Chl *a*	Chl *a*	Chl *a*	Chl *a*	Chl *a*	Chl *a*	Chl *a*	Chl *a*	Chl *a*
310	Chl *a*		**Chl c_2-*MGDG***	Chl *c*		Chl *a*	Chl *c*	**Chl c_2-*MGDG***				Chl *c*	Chl *c*	**Chl c_2-*MGDG***	**Chl c_2-*MGDG***	**Chl c_2-*MGDG***	Chl *c*
311		Chl *a*			Chl *a*	Chl *a*		Chl *a*	Chl *a*	Chl *a*							
312					Chl *a*	Chl *a*	Chl *a*		Chl *a*	Chl *a*							
313												Chl *a*		Chl *a*	Chl *a*	Chl *a*	Chl *a*
314												Chl *c*					Chl *c*
315														Chl *a*	Chl *a*	Chl *a*	
316												Chl *c*					Chl *c*
317												Chl *c*		Chl *c*	Chl *c*	Chl *c*	Chl *c*
318								Chl *a*				Chl *a*	Chl *a*	Chl *a*	Chl *a*	Chl *a*	Chl *a*
319					Chl *a*	Chl *a*											
320																	
321					Chl *c*			Chl *c*	Chl *c*								
322					Chl *a*												
323												Chl *a*					
324				Chl *a*													
325																	
326																	
401		DD6	Ddx	hFx	Ddx	Ddx	Ddx	Fx	Ddx	Ddx	Ddx	Ddx	Fx	GyrE	GyrE	GyrE	Fx
402		Fx	Ddx	hFx	Fx	Ddx	Ddx	Fx	Ddx	Fx	Ddx	hFx	hFx	Fx	Fx	Fx	hFx
403	Ddx	Ddx	Ddx	Ddx	Ddx	Ddx	Ddx	Ddx	Ddx	Ddx	Ddx	Ddx	Ddx	Ddx	Ddx	Ddx	Ddx
404					Ddx	Ddx	Ddx		Ddx	Ddx							
405			Ddx	Ddx	Ddx	Ddx	Ddx	Ddx	Ddx	Ddx	Fx		Fx	Fx	Fx	Fx	Ddx
406								Fx						Fx	Fx	Fx	Fx
407				hFx													
408					Fx												
409																	
410												hFx					Ddx
411				Fx								Fx					Fx
412								Ddx									
413																	
414																	
415																	
416																	

Site	EF 18	EF 19	EF 20	EF 21	EF 22	EF 23	EF 24	EF 25	EF 26	EF 27	EF 28	EF 29	EF 30	EF 31	EF 32	EF 33	EF 34	EF 35
301																		
302	Chl *a*	Chl *a*	Chl *a*	Chl *a*	Chl *a*	Chl *a*	Chl *a*	Chl *a*	Chl *a*	Chl *a*	Chl *a*	Chl *a*	Chl *a*	Chl *a*	Chl *a*			
303	Chl *c*	Chl *c*	Chl *c*	Chl *c*	Chl *c*	Chl *c*	Chl *c*	Chl *c*	Chl *c*	Chl *c*	Chl *c*	Chl *c*	Chl *c*	Chl *c*	Chl *c*			
304	Chl *a*	Chl *c*	Chl *c*	Chl *c*	Chl *c*	Chl *c*	Chl *c*	Chl *c*	Chl *c*	Chl *c*	Chl *c*	Chl *c*	Chl *c*	Chl *c*	Chl *c*	Chl *c*	Chl *c*	Chl *c*
305	Chl *a*	Chl *a*	Chl *a*	Chl *a*	Chl *a*	Chl *a*	Chl *a*	Chl *a*	Chl *a*	Chl *a*	Chl *a*	Chl *a*	Chl *a*	Chl *a*	Chl *a*	Chl *a*	Chl *a*	Chl *a*
306	Chl *a*	Chl *a*	Chl *a*	Chl *a*	Chl *a*	Chl *a*	Chl *a*	Chl *a*	Chl *a*	Chl *a*	Chl *a*	Chl *a*	Chl *a*	Chl *a*	Chl *a*	Chl *a*	Chl *a*	Chl *a*
307	Chl *a*	Chl *a*	Chl *a*	Chl *a*	Chl *a*	Chl *a*	Chl *a*	Chl *a*	Chl *a*	Chl *a*	Chl *a*	Chl *a*	Chl *a*	Chl *a*	Chl *a*	Chl *a*	Chl *a*	Chl *a*
308	Chl *a*																	
309	Chl *a*	Chl *a*	Chl *a*	Chl *a*	Chl *a*	Chl *a*	Chl *a*	Chl *a*	Chl *a*	Chl *a*	Chl *a*	Chl *a*	Chl *a*	Chl *a*	Chl *a*	Chl *a*	Chl *a*	Chl *a*
310		Chl *c*	Chl *c*	Chl *c*	**Chl c_2-MGDG**	Chl *c*	Chl *c*	Chl *c*	Chl *c*	Chl *c*	Chl *c*	Chl *c*	Chl *c*	Chl *c*	Chl *c*	Chl *c*	Chl *c*	Chl *c*
311	Chl *a*																	
312	Chl *a*																	
313		Chl *a*	Chl *a*	Chl *a*	Chl *a*	Chl *a*	Chl *a*	Chl *a*	Chl *a*	Chl *a*	Chl *a*	Chl *a*	Chl *a*	Chl *a*	Chl *a*	Chl *a*	Chl *a*	Chl *a*
314																		
315					Chl *a*	Chl *a*	Chl *a*	Chl *a*	Chl *a*	Chl *a*	Chl *a*	Chl *a*	Chl *a*	Chl *a*	Chl *a*	Chl *a*	Chl *a*	Chl *a*
316					Chl *c*		Chl *c*			Chl *c*	Chl *c*	Chl *c*						
317		Chl *c*	Chl *c*	Chl *c*	Chl *c*	Chl *c*	Chl *c*	Chl *c*	Chl *c*	Chl *c*	Chl *c*	Chl *c*	Chl *c*	Chl *c*	Chl *c*	Chl *c*	Chl *c*	Chl *c*
318		**Chl c_2-MGDG**	**Chl c_2-MGDG**	**Chl c_2-MGDG**	Chl *a*	Chl *a*	Chl *a*	Chl *a*	Chl *a*	Chl *a*	Chl *a*	Chl *a*	Chl *a*	Chl *a*	Chl *a*	Chl *a*	Chl *a*	Chl *a*
319																		
320		Chl *a*	Chl *a*	Chl *a*														
321																		
322																		
323					Chl *a*	Chl *a*	Chl *a*	Chl *a*	Chl *a*	Chl *a*	Chl *a*	Chl *a*	Chl *a*	Chl *a*	Chl *a*			
324																		
325					Chl *a*													
326								Chl *a*										
401	Fx	Fx	Fx	Fx	hFx	Fx	Fx	Fx	Fx	Fx	Fx	Fx	Fx	Fx	Fx			
402	Ddx	Fx	Fx	Fx	Fx	hFx	Fx	Fx	Fx	hFx	hFx	hFx	Fx	Fx	Fx	Fx	Fx	Fx
403	Ddx	Ddx	Ddx	Ddx	hFx	hFx	Fx	hFx	Fx	hFx	hFx	hFx	Fx	hFx	hFx	hFx	hFx	hFx
404	Ddx																	
405	Ddx	Fx	Fx	Fx	Fx	Fx	Fx	Fx	Fx	Fx	Fx	Fx	hFx	Fx	Fx			
406	Fx	hFx	hFx	hFx	Fx	Fx	Fx	Fx	Fx	Fx	Fx	Fx	Fx	Fx	Fx			
407																		
408																		
409													hFx	hFx	hFx			
410																		
411		Fx	Fx	Fx	Fx	Fx	Fx	Fx	Fx	Fx	Fx	Fx	Fx	Fx	Fx		Fx	Fx
412					Fx	hFx	Fx	Fx	Fx	Fx	Fx	Fx	hFx	hFx	hFx			
413						hFx	DD6	DD6	DD6	DD6	DD6	DD6	DD6	DD6	DD6	DD6	DD6	DD6
414						DD6		DD6	Fx	Fx	Fx	Fx	DD6	DD6	DD6	Fx	Fx	Fx
415																hFx	hFx	hFx
416																hFx	hFx	hFx

Table S4. Calculated EET time constant from EFCPIs in fifth and sixth layers to the PSI core based on the generalized Förster theory. Numbers of EET route represent EFCPI-1 to EFCPI-35. Core represent PSI core.

**EET route**	**EET time constant for each step (ps)**						**Total time (ps)**
35-31-29-21-17-7-core	9.5	2.8	22	18.1	11.1	10.3	73.8
35-31-29-21-16-7-core	9.5	2.8	22	3.7	10.8	10.3	59.1
35-31-29-21-16-6-core	9.5	2.8	22	3.7	19.4	16.9	74.3
35-31-29-20-16-7-core	9.5	2.8	21.1	7.2	10.8	10.3	61.7
35-31-29-20-16-6-core	9.5	2.8	21.1	7.2	19.4	16.9	76.9
35-31-29-20-15-6-core	9.5	2.8	21.1	1.7	4.1	16.9	56.1
35-31-29-20-15-5-core	9.5	2.8	21.1	1.7	18.1	13.1	66.3
35-32-28-20-16-7-core	19.2	2.6	12.7	7.2	10.8	10.3	62.8
35-32-28-20-16-6-core	19.2	2.6	12.7	7.2	19.4	16.9	78
35-32-28-20-15-6-core	19.2	2.6	12.7	1.7	4.1	16.9	57.2
35-32-28-20-15-5-core	19.2	2.6	12.7	1.7	18.1	13.1	67.4
35-32-28-19-15-6-core	19.2	2.6	21	7.8	4.1	16.9	71.6
35-32-28-19-15-5-core	19.2	2.6	21	7.8	18.1	13.1	81.8
35-32-28-19-14-5-core	19.2	2.6	21	3.1	0.3	13.1	59.3
35-32-28-19-14-4-core	19.2	2.6	21	3.1	13.7	0.6	60.2
34-32-28-20-16-7-core	12.6	2.6	12.7	7.2	10.8	10.3	56.2
34-32-28-20-16-6-core	12.6	2.6	12.7	7.2	19.4	16.9	71.4
34-32-28-20-15-6-core	12.6	2.6	12.7	1.7	4.1	16.9	50.6
34-32-28-20-15-5-core	12.6	2.6	12.7	1.7	18.1	13.1	60.8
34-32-28-19-15-6-core	12.6	2.6	21	7.8	4.1	16.9	65
34-32-28-19-15-5-core	12.6	2.6	21	7.8	18.1	13.1	75.2
34-32-28-19-14-5-core	12.6	2.6	21	3.1	0.3	13.1	52.7
34-32-28-19-14-4-core	12.6	2.6	21	3.1	13.7	0.6	53.6
33-27-19-15-6-core	13.1	20.2	7.8	4.1	16.9		62.1
33-27-19-15-5-core	13.1	20.2	7.8	18.1	13.1		72.3
33-27-19-14-5-core	13.1	20.2	3.1	0.3	13.1		49.8
33-27-19-14-4-core	13.1	20.2	3.1	13.7	0.6		50.7
33-27-25-14-5-core	13.1	8.7	23.8	0.3	13.1		59
33-27-25-14-4-core	13.1	8.7	23.8	13.7	0.6		59.9
33-25-14-5-core	8.2	23.8	0.3	13.1			45.4
33-25-14-4-core	8.2	23.8	13.7	0.6			46.3
Average time (ps)							62.5

Movie S1. PCA-based 3D Variability Analysis (3DVA) of coccolithophore PSI–EFCPI by CryoSPARC.
